# Evolution of a Major Drug Metabolizing Enzyme Defect in the Domestic Cat and Other Felidae: Phylogenetic Timing and the Role of Hypercarnivory

**DOI:** 10.1371/journal.pone.0018046

**Published:** 2011-03-28

**Authors:** Binu Shrestha, J. Michael Reed, Philip T. Starks, Gretchen E. Kaufman, Jared V. Goldstone, Melody E. Roelke, Stephen J. O'Brien, Klaus-Peter Koepfli, Laurence G. Frank, Michael H. Court

**Affiliations:** 1 Comparative and Molecular Pharmacogenomics Laboratory, Department of Molecular Physiology and Pharmacology, Tufts University School of Medicine, Boston, Massachusetts, United States of America; 2 Department of Biology, Tufts University, Medford, Massachusetts, United States of America; 3 Department of Environmental and Population Health, Tufts Cummings School of Veterinary Medicine, North Grafton, Massachusetts, United States of America; 4 Biology Department, Woods Hole Oceanographic Institution, Woods Hole, Massachusetts, United States of America; 5 Laboratory of Genomic Diversity, SAIC-Frederick Incorporated, National Cancer Institute at Frederick, Frederick, Maryland, United States of America; 6 Laboratory of Genomic Diversity, National Cancer Institute at Frederick, Frederick, Maryland, United States of America; 7 Living with Lions Project (Kenya), Museum of Vertebrate Zoology, University of California, Berkeley, California, United States of America; Dr. Margarete Fischer-Bosch Institute of Clinical Pharmacology, Germany

## Abstract

The domestic cat (*Felis catus*) shows remarkable sensitivity to the adverse effects of phenolic drugs, including acetaminophen and aspirin, as well as structurally-related toxicants found in the diet and environment. This idiosyncrasy results from pseudogenization of the gene encoding UDP-glucuronosyltransferase (UGT) 1A6, the major species-conserved phenol detoxification enzyme. Here, we established the phylogenetic timing of disruptive *UGT1A6* mutations and explored the hypothesis that gene inactivation in cats was enabled by minimal exposure to plant-derived toxicants. Fixation of the *UGT1A6* pseudogene was estimated to have occurred between 35 and 11 million years ago with all extant Felidae having dysfunctional *UGT1A6*. Out of 22 additional taxa sampled, representative of most Carnivora families, only brown hyena (*Parahyaena brunnea*) and northern elephant seal (*Mirounga angustirostris*) showed inactivating *UGT1A6* mutations. A comprehensive literature review of the natural diet of the sampled taxa indicated that all species with defective *UGT1A6* were hypercarnivores (>70% dietary animal matter). Furthermore those species with *UGT1A6* defects showed evidence for reduced amino acid constraint (increased *dN/dS* ratios approaching the neutral selection value of 1.0) as compared with species with intact *UGT1A6.* In contrast, there was no evidence for reduced amino acid constraint for these same species within *UGT1A1,* the gene encoding the enzyme responsible for detoxification of endogenously generated bilirubin. Our results provide the first evidence suggesting that diet may have played a permissive role in the devolution of a mammalian drug metabolizing enzyme. Further work is needed to establish whether these preliminary findings can be generalized to all Carnivora.

## Introduction

Between- and within- species differences in the capacity to metabolize and eliminate drugs and other xenobiotics from the body are typically substantial, complicating the effective use of drugs, as well as minimizing the ability to predict the adverse consequences of environmental pollutants. Slow metabolic clearance leads to enhanced adverse drug effects and the bioaccumulation of pollutants, while fast metabolic clearance minimizes beneficial drug effects. One extreme of the species difference is the so-called ‘species defect’ of drug metabolism - a drug metabolic pathway that is common to most species, but essentially absent in one (or perhaps only a few) species [1]. Perhaps the best known example of a species defect of drug metabolism is the inability of domestic cats to metabolize drugs and structurally related phenolic compounds by glucuronidation [2], [3], [4], [5], [6], [7]. Glucuronidation is catalyzed by the UDP-glucuronosyltransferases (UGTs), a superfamily of conjugative enzymes predominantly found in the liver that transfer glucuronic acid to a drug (or other chemical compound) yielding a nontoxic, more water soluble, and readily excreted glucuronide metabolite [8]. Slow glucuronidation of acetaminophen [7] and acetylsalicylic acid (aspirin) [6] account for the slow clearance and exquisite sensitivity of cats to the adverse effects of these drugs compared with dogs and most other mammalian species.

In previous work, we determined that the main enzyme responsible for detoxification of these phenolic drugs (UGT1A6) is not expressed in cat liver [4], [5], [9]. Furthermore, we showed that the gene encoding UGT1A6 in cats and at least one other species in the Felidae family (i.e. margay; *Felis weidii*) contains multiple inactivating mutations, consistent with *UGT1A6* being a pseudogene in these species [4]. However, as yet it is not known whether this represents a single *UGT1A6* pseudogenization event affecting one particular lineage, or whether multiple independent *UGT1A6* inactivations have occurred either within or beyond the Felidae. In a classical series of radiotracer experiments conducted nearly 40 years ago, glucuronidation of orally administered [^14C^]phenol was found to be deficient in several other families of Carnivora including Viverridae (African civet, forest genet), Hyaenidae (spotted hyena), in addition to all Felidae species examined (African lion, caracal, and domestic cat) [3], [10], [11], [12]. These findings suggested either a more ancient origin of *UGT1A6* loss predating Felidae divergence, or perhaps more recent multiple *UGT1A6* inactivations.

‘Drug’ metabolizing enzymes did not evolve to deal with synthetic human-made drugs, but rather evolved to detoxify environmental chemicals and endogenous metabolites. Some drug metabolizing enzymes may have evolved in animals in large part to detoxify various chemicals found in plants used for food, thereby enabling a broader selection of foods and a survival advantage for the animals that consumed them [4], [13], [14]. A corollary to this is that animals with a diet consisting primarily of animal matter would have little need for such enzymes, and the genes encoding these enzymes would become dysfunctional through either neutral evolution or selection to conserve energy associated with enzyme synthesis (‘use it or lose it’). The Felidae, including the domestic cat, are representative of such a group of highly specialized carnivores (identified as ‘hypercarnivores’) within the mammalian order Carnivora [15]. Consequently, pseudogenization of the *UGT1A6* gene may reflect the loss of selection pressure as an ancestral felid species transitioned from a generalized (plant and animal) to a more specialized (animal only) diet [15]. Given the wide diversity in diets of the extant Carnivora - ranging from hypercarnivores to the more generalist ‘mesocarnivores’ (e.g. dogs and bears) to the mainly plant-eating ‘hypocarnivores’ (e.g. giant panda and red panda) - the order Carnivora provides a unique opportunity to explore the relationship between diet and evolution of the drug metabolizing enzymes.

The main purpose of the present study was to accurately establish the extent and phylogenetic timing of the Felidae *UGT1A6* pseudogenization. We also explored whether this was a unique event, or may have been recapitulated in other Carnivora, as a consequence of relaxation of purifying selection of the *UGT1A6* gene in those species with a highly carnivorous diet.

## Results

### *UGT1A6* pseudogenization occurred prior to *Felidae* divergence

*UGT1A6* exon 1 sequences were determined for representative taxa of eight established lineages within the Felidae [16] to ascertain the extent of species affected and approximate timing of pseudogenization. *UGT1A1* exon 1 sequences were also evaluated in parallel as a positive control since it encodes the essential detoxifying enzyme for the endogenous substrate bilirubin, at least in humans [17], and was expected to be well conserved between species. Sampling focused on the exon 1 sequence since both *UGT1A1* and *UGT1A6* are encoded by the same gene locus (*UGT1A*) through alternate splicing of unique exons 1 (substrate binding domain) to shared exons 2 to 5 (UDP-glucuronic acid binding domain) [8].

**Figure 1:**
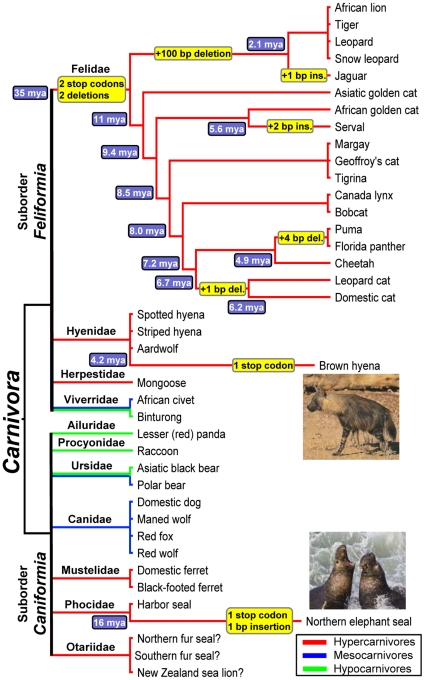
Carnivora phylogeny, *UGT1A6* mutations, and diet. Shown is a simplified phylogeny of the Carnivora species evaluated in this study, indicating the deduced timing of disruptive *UGT1A6* mutations (highlighted) found within the Felidae, Hyaenidae and Phocidae lineages. Shown at specific nodes are divergence times (in MYA) defining the upper and/or lower boundaries of each mutation based on published estimates [16], [18], [19] (details provided in Table 1). The inferred diets of each species based on the system proposed by Van Valkenburgh [27], [35] are denoted by the branch line color. Species were classified as hypercarnivores (>70% animal matter in diet), mesocarnivores (50-70% animal matter in diet), or hypocarnivores (<50% animal matter in diet) based on evidence given in Tables S2 and S3. Disruptive *UGT1A6* mutations are only found in species classified as hypercarnivores. Note that for unknown reasons *UGT1A6* could not be amplified by PCR in any of the Otariidae evaluated (indicated by “?”), while *UGT1A1* was readily amplified and sequenced in those same species.

*UGT1A1* and *UGT1A6* exon 1 sequences were successfully characterized for all Felidae species evaluated (Table S1). Analysis of the *UGT1A1* exon 1 sequences (Fig. S1) showed complete reading frames in all species that matched well with known *UGT1A1* sequences. In contrast, all of the felid *UGT1A6* exon 1 sequences (Fig. S2) showed multiple mutations located within the coding region that either alter the reading frame, or directly result in premature stop codons. As shown in Table 1 and Fig. 1, out of the 9 unique mutations that were identified, four were shared by all of the felid species evaluated, including two stop codons (M1 and M2) and two frame shift deletions (M3 and M4). A one bp frameshift deletion (M5) was also found in both domestic cat and leopard cat lineages, while a large 100 bp frameshift deletion (M6) was found in all evaluated species in the Panthera lineage. The remaining mutations (frameshift insertion/deletions) were associated with individual species within the puma (M7), caracal (M8), and Panthera (M9) lineages.

**Table 1:** Mutations disrupting the reading frame of the UGT1A6 gene found in 18 different species of Felidae.

			UGT1A6 mutations (ID, type, location^1^, estimated fixation time^2^ and presence/absence in each species)								
			M6	M1	M7	M2	M3	M4	M8	M9	M5
			100 bp del.	Stop codon	4 bp del.	Stop codon	1 bp del.	10 bp del.	2 bp ins.	1 bp ins.	1 bp del.
			bp 9-108	bp 274-276	bp 361-364	bp 379-381	bp 398-399	bp 660-669	bp 691-692	bp 768-769	bp 827
Common name	Species	Lineage^2^	3.7–10.8 MYA	>10.8 MYA	<4.9 MYA	>10.8 MYA	>10.8 MYA	>10.8 MYA	<5.6 MYA	<2.1 MYA	6.2–6.7 MYA
Domestic cat	*Felis catus*	Domestic cat	-	+	-	+	+	+	-	-	+
Leopard cat	*Prionallurus bengalensis*	Leopard cat	-	+	-	+	+	+	-	-	+
Puma	*Puma concolor*	Puma	-	+	+	+	+	+	-	-	-
Florida panther	*Puma concolor coryi*	Puma	-	+	+	+	+	+	-	-	-
Cheetah	*Acinonyx jubatus*	Puma	-	+	-	+	+	+	-	-	-
Canada lynx	*Lynx canadensis*	Lynx	-	+	-	+	+	+	-	-	-
Bobcat	*Lynx rufus*	Lynx	-	+	-	+	+	+	-	-	-
Geoffroy's cat	*Leopardus geoffroyi*	Ocelot	-	+	-	+	+	+	-	-	-
Margay	*Leopardus wiedii*	Ocelot	-	+	-	+	+	+	-	-	-
Tigrina	*Leopardus tigrinus*	Ocelot	-	+	-	+	+	+	-	-	-
African golden cat	*Caracal aurata*	Caracal	-	+	-	+	+	+	-	-	-
Serval	*Caracal serval*	Caracal	-	+	-	+	+	+	+	-	-
Asian golden cat	*Pardofelis temminckii*	Bay cat	-	+	-	+	+	+	-	-	-
Jaguar	*Panthera onca*	Panthera	+	+	-	+	+	+	-	+	-
Lion	*Panthera leo*	Panthera	+	+	-	+	+	+	-	-	-
Leopard	*Panthera pardus*	Panthera	+	+	-	+	+	+	-	-	-
Tiger	*Panthera tigris*	Panthera	+	+	-	+	+	+	-	-	-
Snow leopard	*Panthera uncia*	Panthera	+	+	-	+	+	+	-	-	-

(+) and (-) denotes presence or absence of inactivating coding sequence mutation in that species UGT1A6 sequence.

MYA - millions of years ago. bp - base pairs.

1 Nucleotide position relative to adenine (+1) of start codon ATG of human UGT1A6 exon1 (GenBank accession no.- M84130).

2 Felidae lineages and divergence dates used to estimate mutation fixation timing (in MYA) were derived from Table 1 in Johnson et al, 2005.

### *UGT1A6* gene disruptions are found in other Carnivora species

**Figure 2:**
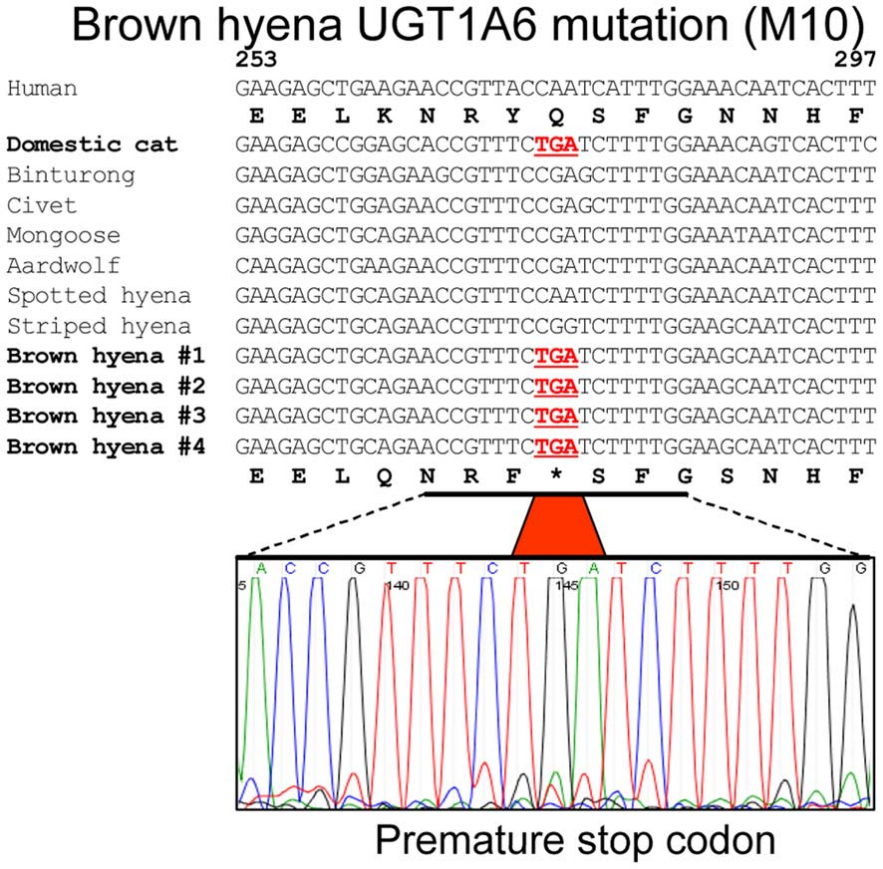
Premature stop codon found in the brown hyena (*Parahyaena brunnea*) *UGT1A6* coding sequence. Shown are the *UGT1A6* exon 1 nucleotide sequences (bp 253-297) of four brown hyenas aligned with *UGT1A6* sequences from other species of Feliformia. A premature stop codon TGA (M10: bp274-276) was found in all brown hyenas evaluated at exactly the same position as the premature stop codon TGA (M1) found in the domestic cat and all other felid species evaluated. Also shown are a representative DNA sequence chromatogram, and the translated brown hyena and human UGT1A6 amino acid sequences.

Since all felid species evaluated showed multiple *UGT1A6* mutations, the search was expanded beyond Felidae to include all 4 species within Hyaenidae, as well as representative taxa from other families within the suborder Feliformia including binturong and African civet (both Viverridae), and mongoose (Herpestidae). As shown in Fig. 2 and Fig. S2, intact *UGT1A6* coding sequences were found for all species except brown hyena which showed a premature stop codon (M10) at the same codon position and identical in nucleotide sequence (i.e. ‘TGA’) to the nonsense codon mutation (M1) first described in domestic cat and shared by all felids. Sequencing of DNA samples obtained from four different brown hyenas yielded identical results (Fig. 2).

**Figure 3:**
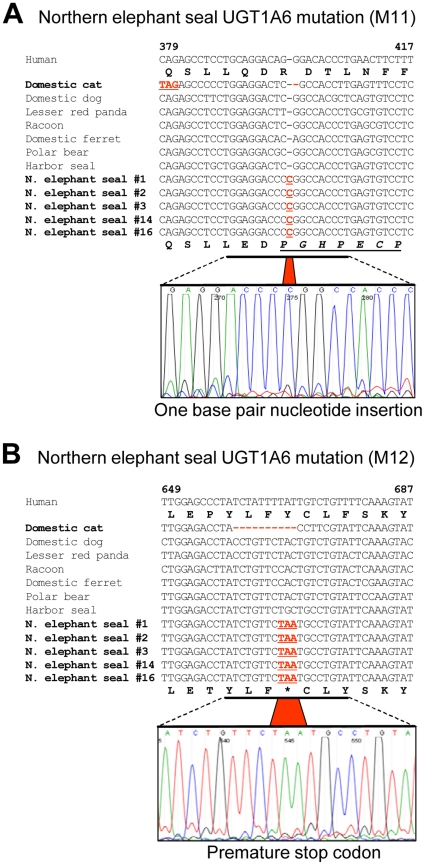
Frameshift mutation and premature stop codon found in the northern elephant seal (*Mirounga angustirostris*) *UGT1A6* coding sequence. Shown in panel (A) are the *UGT1A6* exon 1 nucleotide sequences (bp 379-417) of 5 different northern elephant seals aligned with *UGT1A6* sequences from other species of Caniformia, domestic cat, and human. A 1 bp insertion (M11: bp 398-399) was found in all northern elephant seals evaluated resulting in a reading frame shift relative to other species. Shown in panel (B) is the premature stop codon TAA (M12: bp 667-669) found in all northern elephant seals. Also shown in panels (A) and (B) are representative DNA chromatograms, and the translated northern elephant seal and human UGT1A6 amino acid sequences.

Since the M10 mutation in brown hyena may have arisen independently of the mutations found in Felidae, we expanded our search for the presence of similarly disruptive mutations to include taxa representative of most other Carnivora families (with the exception of Eupleridae, Mephitidae, and Odobenidae). *UGT1A6* and *UGT1A1* exon 1 sequences could be determined for most species evaluated (Table S1) except for *UGT1A6* in southern fur seal, northern fur seal, and New Zealand sea lion (all in the family Otariidae) and *UGT1A1* in the red panda. Northern elephant seal was the only species other than brown hyena and all of the Felidae that showed disruptive coding sequence mutations in the *UGT1A6* gene. Two separate mutations were identified including a 1 bp insertion (M11: bp 398–399) resulting in a frame shift with associated premature stop codons (Fig. 3A), and an in-frame stop codon (M12: bp 667–669) (Fig. 3B). Interestingly, these mutations were co-localized with 2 of the 4 founding felid mutations, including M3 (1 bp deletion at position 399) and M4 (10 bp deletion at position 660–669). Sequencing of DNA samples collected from five northern elephant seals derived from two different populations showed identical results (Fig. 3). No disruptive mutations were detected in any of the *UGT1A1* sequences evaluated.

### Phylogenetic timing of the *UGT1A6* mutations

Using the divergence times established by Johnson *et al* [16] and Koepfli *et al* [18] with relaxed molecular clock analyses combined with fossil calibration, it was possible to determine approximate timings for fixation of each of the felid *UGT1A6* mutations (Fig. 1 with details in Table 1). Fixation of the four shared mutations (M1-4) occurred between 10.8 (CI: 8.4–14.5) and 36.5 (CI: 28.9–46.5) million years ago (MYA) representing estimated dates for divergence of the extant felid lineages, and for divergence of the Felidae family from all other feliformia families, respectively. The remaining *UGT1A6* mutations within felids arose more recently within certain lineages, with the most recent occurring in the jaguar less than 2.1 (CI: 1.2–3.5) MYA.

Since neither the aardwolf, striped hyena nor spotted hyena showed disruptive *UGT1A6* mutations, the brown hyena M10 mutation most likely arose following the divergence of brown hyena from other hyaenid species approximately 4.2 (CI: 2.6–6.4) MYA [18]. Similarly, the northern elephant seal M11 and M12 mutations likely arose following divergence of ancestors of the northern elephant seal and harbor seal approximately 16 (CI: 14.2–17.8) MYA [19].

### Felidae and Phocidae show reduced *UGT1A6* amino acid sequence constraint

We next evaluated differences in the strength of *UGT1A6* and *UGT1A1* amino acid sequence fixation (as reflected by the *dN/dS* ratio) between the various lineages of Carnivora. As shown in Table 2, the average *dN/dS* ratio (an estimate using sequence data from all species) was substantially less (*P* < 0.05; likelihood ratio test) than the expected neutral evolution value of 1.0 for both *UGT1A6* (0.39) and *UGT1A1* (0.38), consistent with purifying selection acting on both of these genes. However, when the Felidae lineage was considered separately, the *UGT1A6 dN/dS* ratio (0.68) was significantly higher (*P* = 0.011; likelihood ratio test) as compared with the average *UGT1A6 dN/dS* ratio (0.39). This result is consistent with reduced amino acid constraint in the felid *UGT1A6* gene. In contrast the felid *UGT1A1 dN/dS* ratio (0.45) was not significantly different (*P* = 0.56) from the average *UGT1A1* value (0.38). A similar trend was also observed for the phocids in that the *UGT1A6 dN/dS* ratio (1.17) was more than 2-fold higher (*P* = 0.009) compared with the average *UGT1A6* dN/dS value (0.39), while the phocid *UGT1A1 dN/dS* ratio (0.71) was not significantly different (*P* = 0.56) from the average *UGT1A1* value (0.38). None of the remaining carnivoran lineages showed *UGT1A1* or *UGT1A6 dN/dS* ratios that were significantly different from the average *dN/dS* ratio for the respective gene. Although a somewhat higher *dN/dS* ratio was obtained for ursid *UGT1A1* (1.34), the difference from the average UGT1A1 *dN/dS* ratio did not achieve statistical significance (*P* = 0.06). All of the non-Carnivora species with available sequence data had *dN/dS* values for *UGT1A6* (0.36 to 0.44) and *UGT1A1* (0.33 to 0.47) that were indistinguishable from the average ratios for each gene (*P* > 0.05).

**Table 2:** Nonsynonymous to synonymous nucleotide substitution frequency ratios (dN/dS) determined for Carnivora and non-Carnivora UGT genes using a maximum likelihood approach.

		UGT1A6			UGT1A1		
Order (sub-order)	Family	dN/dS	*P* value^1^	N taxa (seq.)^2^	dN/dS	*P* value^1^	N taxa (seq.)^2^
	All species (average value)	0.3926	Null model	49 (50)	0.3811	Null model	47 (38)
Carnivora (Feliformia)	Felidae	0.6785	0.011	18 (16)	0.4476	NS	18 (15)
	Hyenidae	0.508	NS	4 (4)	0.5258	NS	4 (3)
	Herpestidae	0.2533	NS	1 (1)	0.2171	NS	1 (1)
	Viverridae	0.4815	NS	2 (2)	0.3556	NS	2 (2)
Carnivora (Caniformia)	Ursidae	0.2148	NS	2 (2)	0.9952	NS	2 (1)
	Procyonidae	0.4983	NS	1 (1)	0.2866	NS	1 (1)
	Ailuridae	0.2102	NS	2 (1)	-	-	0 (0)
	Mustelidae	0.3215	NS	2 (2)	0.2832	NS	2 (2)
	Otariidae	-	-	0 (0)	>999	NS	3 (1)
	Phocidae	1.1708	0.009	2 (2)	0.7129	NS	2 (2)
	Canidae	0.1826	NS	4 (4)	0.1211	NS	4 (3)
Non-Carnivora	Cattle, sheep, pig, horse	0.3945	NS	4 (6)	0.4166	NS	1 (1)
	Mouse, rat, rabbit	0.3659	NS	3 (4)	0.4112	NS	2 (2)
	Primates	0.3969	NS	5 (5)	0.4127	NS	5 (4)

1 *P* value for likelihood ratio test comparing log-likelihood values obtained from a branch model in which dN/dS values were estimated for the lineage of interest (alternative model) and an equivalent model (null model) in which the lineage dN/dS value was fixed to the value originally obtained for all species (average value). *P*<0.05 was considered significant with one degree of freedom.

2 Number of sampled taxa and unique translated amino acid sequences (seq.) used in each analysis. Differences between the numbers of taxa and sequences within each group arise from the presence of multiple UGT1A6 genes in mouse (2) and horse (3), as well as exclusion of any sequences found to be identical to any other sequence after cropping (see Table S1 and Table S6 for details).

### All species with reduced *UGT1A6* amino acid sequence constraint are hypercarnivores

Diet is proposed to profoundly influence the evolution of the drug metabolizing enzymes [13], [14]. Consequently low dietary content of plant-derived phenolic intoxicants may have been one factor that enabled pseudogenization of an otherwise broadly conserved mammalian gene as *UGT1A6*. Out of the 40 species of Carnivora evaluated here, 30 species (including all Felidae, Hyaenidae, Herpestidae, Mustelidae, Otariidae, and Phocidae) could be classified as hypercarnivores, 6 species (all Canidae, polar bear and African civet) were classified as mesocarnivores, while 4 species (raccoon, red panda, Asiatic black bear and binturong) were classified as hypocarnivores (Fig. 1 and Table S2). Additional support for this classification was gained from an analysis of the protein contents of commercial diets required to maintain optimum health of captive Carnivora (Table S3). Ferrets and all Felidae (hypercarnivores) required the highest protein content (35–38% w/w), while polar bear and all Canidae species (mesocarnivores) required an intermediate protein content (28.5–30.5% w/w). Furthermore, bears (other than polar bear) and raccoon (hypocarnivores) required the lowest protein content (25% w/w).

With respect to *UGT1A6* amino acid sequence constraint, the two lineages that showed significant relaxation of *UGT1A6* constraint (Felidae and Phocidae) consisted solely of species classified as hypercarnivores. The Hyaenidae, which also consisted solely of hypercarnivores, also showed some evidence for reduced *UGT1A6* amino acid constraint (*dN/dS* ratio of 0.51 versus an average *dN/dS* value of 0.39), although the difference did not achieve statistical significance (*P* > 0.05). However, the remaining hypercarnivore lineages evaluated (Mustelidae and Herpestidae) showed no evidence for altered *UGT1A6* amino acid constraint relative to other species (Table 2). There was no clear trend for altered *UGT1A6* constraint in the remaining lineages consisting of either mesocarnivores (Canidae), hypocarnivores (Ailuridae and Procyonidae), or both mesocarnivores and hypocarnivores (Ursidae and Viverridae) In contrast to *UGT1A6*, none of the lineages examined (including Phocidae and Felidae) showed altered *UGT1A1* amino acid constraint (Table 2).

## Discussion

To the best of our knowledge, this is the first study to identify the phylogenetic origin of a major drug metabolism deficiency during the evolution of a mammalian species. Although deficiency of another major drug metabolizing enzyme activity (N-acetyltransferase) was demonstrated to result from the absence of detectable *NAT* genes in multiple species of Canidae [20], the mechanism for the loss of gene function is unknown, as is the timing of the loss with respect to canid evolution.

Our results indicate that complete loss of *UGT1A6* mediated glucuronosyltransferase activity occurred via pseudogene fixation following divergence of the Felidae from all other feliform families approximately 37 MYA, and prior to the initial divergence of the extant felid lineages 11 MYA. More precise timing could be gained from an analysis of *UGT1A6* in the Asiatic linsang (genus *Prionodon*), which were originally thought to be viverids, but based on recent molecular genetic analysis are now considered a sister group to the felids, diverging from them approximately 33 MYA [21].

Interestingly, brown hyena *UGT1A6* possessed a single disruptive mutation (M10) that was identical in nucleotide sequence and location to one of the mutations (M1) found in all felids. While it is possible that both these mutations may have arisen as a single event within a common feliform ancestral species, it is more likely that M10 arose independently and more recently than M1 as a homoplastic mutational event within a hyper-mutable site (‘DNA hotspot’) in the *UGT1A6* coding region. The ancestral codon sequence at this location may have been a ‘CGA’ arginine codon, as is found in another hyaenid species, the aardwolf, as well as in several other feliform species (Fig. 2), which includes a CpG dinucleotide consensus sequence (‘CG’). In addition to methylation of cytosines at CpG sites being a well-known epigenetic mechanism for gene regulation, 5-methylcytosines have the propensity for transition mutation through spontaneous deamination and repair to form thymidines [22]. Consequently, a sense strand C>T mutation of the ancestral ‘CGA’ codon would result in the ‘TGA’ stop codon as is found in the Felidae and brown hyena, or an antisense strand C>T mutation (G>A on the sense strand) would result in the ‘CAA’ glutamine codon as is found in the spotted hyena and also all primates (see Fig. 2 and Fig. S2).

In contrast to the brown hyena *UGT1A6* mutation (M10), both of the reading frame mutations identified in northern elephant seal *UGT1A6* were clearly unrelated to those identified in Felidae *UGT1A6*. However, like the brown hyena, the northern elephant seal demonstrated relatively few adverse *UGT1A6* mutations (two) as compared with felids (four or more mutations), which along with the lack of mutations in other hyaenids and phocids suggests a relatively recent origin in both instances. Unfortunately, we were limited in the number of DNA samples we were able to acquire from different animals within each of these species, and so it is not clear whether our findings can be generalized to the entire population (pseudogene fixation has occurred), or whether functional alleles might still persist either as a polymorphism or rare variant. Future studies that include sampling across brown hyena and northern elephant seal populations are needed to explore such possibilities.

We were unable to amplify and sequence the *UGT1A6* gene in any of the three otariid species we sampled, despite using a variety of PCR primer sets that had worked in all other species, and readily obtaining the *UGT1A1* gene sequence in all three species. This could be the result of more substantial divergence in the *UGT1A6* sequence in this family as compared with other Carnivora families, or perhaps partial or complete deletion of the *UGT1A6* gene. Other genetic techniques could be employed in future studies to explore these possibilities.

We also explored whether there was evidence for relaxation of evolutionary constraint on the *UGT1A6* amino acid coding sequence in affected lineages (brown hyena, northern elephant seal and felid) that might enable the appearance of deleterious mutations and subsequent pseudogene fixation. Confirming our hypothesis, we determined that all lineages with adverse *UGT1A6* mutations demonstrated *dN/dS* values closer to 1.0 (i.e. the expected value for neutral selection) than all other species. Although the *dN/dS* estimate for felid *UGT1A6* (0.68) was clearly higher than estimates for other lineages (except Phocidae), it was not 1.0, which is the value we expected for a noncoding pseudogene that should be evolving neutrally. Although there are relatively few published studies that give *dN/dS* ratio estimates for large numbers of pseudogenes, in each instance a substantial proportion of the identified pseudogenes were found to have *dN/dS* values substantially less than 1.0 [23], [24], [25], [26]. The reason for the apparent discrepancy is not known but current nucleotide substitution models may overestimate *dS* and underestimate *dN* [26]. We evaluated effects of different available nucleotide substitution and codon bias models and observed only a minimal effect on felid *UGT1A6 dN/dS* estimates. Transcribed (but untranslated) pseudogenes may also play a role in the regulation of orthologous (translated) genes through an RNA interference mechanism, and so the low *dN/dS* values may indirectly reflect purifying selection acting on the protein coding region of the regulated orthologous gene [24]. It is not clear whether *UGT1A6* is transcribed in any felid species, although we have previously ascertained that fully spliced *UGT1A6* mRNA is not expressed in domestic cat liver [4].

Given evidence for reduced purifying selection of the *UGT1A6* gene within certain lineages of Carnivora, we next explored the possible association of this relaxed constraint with diet, specifically hypercarnivory. The analysis suggests that hypercarnivory may be a prerequisite for relaxed constraint and the appearance of deleterious *UGT1A6* mutations. However, not all identified hypercarnivore species demonstrated this association in that ferrets and mongoose were classified as hypercarnivores but demonstrated relatively low *UGT1A6 dN/dS* ratios (0.32 and 0.25 for Mustelidae and Herpestidae lineages, respectively). Since we limited our dietary classification to those species for which we had available DNA sequence within each lineage, it is possible that hypercarnivory may not generalize to the entire lineage. Furthermore, hypercarnivory could be a relatively recent dietary behavior in ferrets and mongoose (or even the Mustelidae and Herpestidae lineages as a whole) and so there might not have been sufficient time to affect *UGT1A6 dN/dS* estimates. Alternatively, the definition of hypercarnivory we used (based on that proposed by Van Valkenburgh [27]) may have been insufficiently stringent. These possibilities could be explored by a more complete analysis of Mustelidae and Herpestidae species.

While previous studies indicated that phenolic glucuronidation was undetectable in African civet and spotted hyena [10], [12], our results suggest that this phenotype is not a consequence of adverse mutations in the *UGT1A6* coding region of these hypercarnivorous species. We have previously shown that acetaminophen glucuronidation by domestic ferret liver is also quite low, although ferret *UGT1A6* contains no reading frame errors [28]. Consequently, other factors in addition to diet may be needed to enable *UGT1A6* pseudogene fixation such as genetic drift or population bottleneck. Interestingly, the late Miocene radiation of the modern Felidae follows the so-called “cat gap” - a prolonged period (23 to 17.5 MYA) during which few felid fossils have been identified [29]. More recently, the Northern elephant seal has undergone a well documented population bottleneck [30].

Beyond *UGT1A6*, there is considerable evidence for loss of function of other genes in the domestic cat that may also be adaptations to hypercarnivory as we have proposed for *UGT1A6* [15]. For example, cats possess very low levels of salivary amylase, an enzyme responsible for initial carbohydrate digestion [31]. They also cannot synthesize taurine from cysteine, vitamin A from carotene, and arachidonate from linoleate and so must receive each of these essential compounds directly from the diet or risk developing nutritional diseases such as blindness and cardiomyopathy [15]. Although it is thought that other felid species are likely to have such enzyme deficiencies, as yet the molecular genetic basis for these deficiencies is unknown. Given the importance of appropriate nutrition for captive breeding of endangered species of Carnivora, it would be of substantial importance to identify the molecular basis for these deficiencies in the cat and establish the extent of the defect in other species, much as we have done with *UGT1A6*.

One diet-related idiosyncrasy of cats that has been elucidated at the molecular level is the lack of preference of cats for sweet (i.e. sugar-containing) foods resulting from pseudogenization of the *Tas1r2* taste receptor gene [32]. Since dietary sugars most likely originate from plant-based sources (such as fruits and berries), *Tas1r2* pseudogenization may also be related to the hypercarnivorous diet of cats. Indeed, other Felidae species, including lion, tiger and cheetah, also demonstrated the *Tas1r2* gene defect, while Herpestidae (mongoose, meerkat), Viverridae (genet), Ailuridae (red panda), Canidae (domestic dog) or Mustelidae (ferret) [32], [33] have an intact *Tas1r2* gene. Behavioral studies also suggest that the lack of sweet taste preference is isolated to the Felidae [33]. Given the remarkable parallels in those results with the findings of the present study, it would be interesting to expand the evaluation of *Tas1r2* genetic mutations and sweet preference to include brown hyena and northern elephant seal.

There are several limitations to the current study that should be mentioned. Other than the Felidae, our survey of representative carnivoran *UGT1A6* and *UGT1A1* sequences was rather limited and so our findings with regard to the possible relationship between diet, UGT1A6 amino acid constraint, and pseudogenization should be viewed with caution. Nevertheless the results of the present study provide justification for proceeding with a more in-depth analysis of the Carnivora. The *UGT1A* gene structure is also unknown for most of the analyzed species so it is possible that some of the species analyzed may have had additional *UGT1A6* copies (as is found in the horse and mouse) that we may have inadvertently missed. Finally, the dietary information used to classify species was quite limited and in many instances quantitative data (such as scat analysis or direct observation) was lacking.

In conclusion, our results substantiate that *UGT1A6* pseudogenization occurred during establishment of the Felidae lineage such that all extant felids are predicted to be deficient in the glucuronidation of phenolic xenobiotics. Furthermore, we provide evidence that *UGT1A6* gene inactivation may have been recapitulated within several other carnivoran lineages, which, like the Felidae, are all hypercarnivores and display reduced *UGT1A6* amino acid fixation rates. *UGT1A6* is likely representative of a set of mammalian genes (including *Tas1r2*) that are essential for effective utilization of plants as a nutritional source, but dispensable during adaption to a primarily animal-based diet. These findings may provide the basis for developing a rational framework for understanding species differences in drug metabolism and disposition, beyond *UGT1A6*.

## Materials and Methods

### Ethics statement

All tissue samples used in this study were obtained with approval of the Institutional Animal Care and Use Committees (IACUC) at Tufts University (M.H.C.) and the National Cancer Institute (S.J.O.). Appropriate permissions were also obtained by the National Cancer Institute (S.J.O.) for use of tissues covered by the Convention on International Trade in Endangered Species (CITES).

### Taxon sampling, DNA amplification, and sequencing

The types and sources of samples used in this study to derive genomic DNA from the study species are listed in detail in Table S4. In many instances we were able to obtain samples from multiple unrelated animals within each species sampled. The genus and species names used follow that of Nowak (2005) [34]. A series of both degenerate and non-degenerate PCR primers specific for *UGT1A1* and *UGT1A6* (but conserved between species) were designed by alignment of all available *UGT1A1* and 1A6 exon 1 gene sequences identified by BLAST search of the Genbank database (Table S5). PCR amplification of 20 ng genomic DNA was performed using a touchdown thermal cycling method and PCR products sequenced directly. PCR product identities were initially confirmed as either *UGT1A1* or *UGT1A6* (and not any other *UGT1A* gene) by phylogenetic tree analysis (neighbor-joining) inputting all available mammalian UGT sequences (listed at http://www.flinders.edu.au/medicine/sites/clinical-pharmacology/ugt-homepage.cfm). Primer pairs that successfully amplified *UGT1A1* and *UGT1A6* for each species are given in Table S5.

### Identification of insertion, deletion, frame-shift, and protein truncation mutations

Insertion and deletion mutations were identified by alignment of novel nucleotide sequences with those of existing *UGT1A1* and *UGT1A6* exon 1 sequences. The effect of each insertion or deletion mutation on the encoded amino acid sequence (insertion or deletion of amino acids, or reading frame shift) was confirmed by virtual translation analysis. Nonsense codon mutations resulting in premature translation stop with truncated protein were also identified by virtual translation analysis. All identified mutations were confirmed by direct visualization of DNA sequence chromatograms, and by sequencing additional DNA samples (when available) obtained from unrelated animals of the same species.

### Phylogenetic tree construction

*UGT1A1* and *UGT1A6* sequences were aligned by Clustal X, adjusted manually, and trimmed to remove overhangs. Trees were constructed independently for *UGT1A1* and *UGT1A6* using multiple approaches including maximum parsimony (PHYLIP Ver. 3.6), maximum likelihood estimation (RAxML Ver. 7.0) and Bayesian inference (MrBayes Ver. 3.1). In each instance, human *UGT1A9* (Genbank ID NM021027) was used as the out-group. A general time reversible plus gamma model of DNA sequence evolution was used based on a comparison of available models using MODELTEST. Reliability of tree estimates was evaluated by bootstrap resampling (1000x) or Bayesian posterior probabilities.

### Nucleotide substitution rate analysis

The nonsynonymous (*N*) to synonymous (*S*) nucleotide substitution rate ratio (*dN/dS*) for each UGT coding sequence (*UGT1A1* and *UGT1A6*) were estimated using a maximum likelihood approach (CODEML module in PAML Ver. 4.4). *dN/dS* values were determined for all species using the basic model (Model 0) and for specific lineages (Felidae and each Carnivora family) using the branch model (Model 2). Estimates were made using each of the input trees shown in Fig. S3 that were generated by using the three different phylogenetic methods described above. Since results were similar regardless of the tree method, the results presented in the text and in Table 2 were generated using the maximum likelihood trees, while complete results are provided in Table S6.

The significance of differences in *dN/dS* values between an individual lineage and those derived for all sequences was evaluated using a likelihood ratio test (*P*<0.05 considered statistically significant). Log-likelihood values obtained from a branch model in which *dN/dS* values were estimated for the lineage of interest (alternative model) were compared to log-likelihood values from an equivalent model (null model) in which the lineage *dN/dS* value was fixed to the value originally obtained for all species (average value). A similar approach was used to evaluate differences in *dN/dS* values from 1.0 (the expected neutral evolution value). One degree of freedom was assumed, representing the difference in the number of free parameters between the tested models.

### Classification of species based on diet

All species of Carnivora evaluated in this study were classified as either hypercarnivores (more than 70% animal matter in diet), mesocarnivores (50 to 70% animal matter), or hypocarnivores (less than 50% animal matter in diet) based on the system previously proposed by Van Valkenburgh [27], [35] using observed or inferred composition of the diets of these animals in their natural environment. Complete details of the reference materials used to classify the species are given in Table S2. Additional support for this classification was inferred from an evaluation of the different minimum protein levels in commercial diets used to feed various Carnivora species maintained in captivity, including zoos and wild animal parks (Table S3). These levels were based on empirical and experimental data and are considered the minimum protein content in order to maintain optimum health for an adult animal.

## Supporting Information

Figure S1 **Clustal X alignment of** ***UGT1A1*** **exon 1 sequences.** No premature stop or frameshift mutations were identified within the coding region. See Table S1 for the full species and common names corresponding to the species abbreviation given on the left side of each sequence.
(PDF)

Figure S2 **Clustal X alignment of** ***UGT1A6*** **exon 1 sequences.** Inactivating mutations (highlighted in red; M1 to M12) within the coding region were defined as either a nucleotide sequence insertion or deletion non-divisible by 3, or a nucleotide substitution resulting in a nonsense (premature stop) codon. Mutation sequence positions (in bp) are relative to the adenine (+1) of the human *UGT1A6* start codon. See Table S1 for the full species and common names corresponding to the species abbreviation given on the left side of each sequence.
(PDF)

Figure S3 **Phylogenetic trees constructed for** ***UGT1A1*** **and** ***UGT1A6*** **exon 1 sequences using three different inference methods.** A. *UGT1A1* maximum likelihood tree (RAxML, Ver. 7.0). B. *UGT1A6* maximum likelihood tree (RAxML, Ver. 7.0). C. *UGT1A1* Bayesian tree (MrBayes, Ver. 3.1) D. *UGT1A6* Bayesian tree (MrBayes, Ver. 3.1) E. *UGT1A1* maximum parsimony tree (PHYLIP, Ver. 3.6) F. *UGT1A6* maximum parsimony tree (PHYLIP, Ver. 3.6). Bootstrap resampling confidence values as percentages (ML and MP trees) or posterior probabilities as ratios (Bayesian trees) are shown for each node.
(PDF)

Table S1 Genbank IDs of novel and existing UGT1A1 and UGT1A6 exon 1 sequences evaluated in this study.
(PDF)

Table S2 Classification of species based on observed dietary behavior or inferred from the literature.
(PDF)

Table S3 Protein content of commercial zoo animal diets formulated for various Carnivora in relation to the dietary classification proposed in this study.
(PDF)

Table S4 Origin of DNA samples used for sequencing in this study.
(PDF)

Table S5 PCR primers that successfully amplified UGT1A1 and UGT1A6 exons 1.
(PDF)

Table S6 Nonsynonymous to synonymous nucleotide substitution frequency ratios (dN/dS) for Carnivora UGT genes obtained using 3 different input tree topologies.
(PDF)
