# Supplementary material for: Evolution of a Major Drug Metabolizing Enzyme Defect in the Domestic Cat and Other Felidae: Phylogenetic Timing and the Role of Hypercarnivory
Source: PLoS One. 2011 Mar 28;6(3):e18046. doi: 10.1371/journal.pone.0018046 (PMC3065456; doi:10.1371/journal.pone.0018046)
Supplement: Figure S1 — Clustal X alignment of UGT1A1 exon 1 sequences. No premature stop or frameshift mutations were identified within the coding region. See Table S1 for the full species and common names corresponding to the species abbreviation given on the left side of each sequence. (PDF) [file pone.0018046.s001.pdf]

```
                   1                                                                         75
MusMus_1A1    (1) ATGACTGTGGTGTGCTGGAGCTCGCGTTTGCTTCTGCTTCTTCCGTACCTTCTGTTGTGTGTGTTCGGTCCCTCT
RatNor_1A1    (1) ATGTCCGTGGTGTGCCGGAGCTCATGTTCGCTTCTGCTTCTTCCGTGCCTTCTGCTGTGTGTGTTGGGTCCCTCT
GalGal_1A1    (1) ------ATGGCCCTGGTCCTTCCTTCTCATCCACAAGTCTCGGTGTCGCTGCTTCTGCTGCTGTCTGTGCTGAGC
HomSap_1A1    (1) ------ATGGCTGTGGAGTCCCAGGGCGGACGCCCACTTGTCCTGGGCCTGCTGCTGTGTGTGCTGGGCCCAGTG
PanTro_1A1    (1) ------ATGGCTGTGGAGTCCCAGGGCGGACGCCCACTTGTCCTGGGCCTGCTGCTGTGTGTGCTGGGCCCAGTG
MacMul_1A1    (1) ------ATGGCTGTGGAGTCCCAAGGCAGACATCCACTTGTCCTGGGCCTGCTGCTGTGTGTGCTGGGCCCAGTG
MacFas_1A1    (1) ------ATGGCTGTGGAGTCCCAAGGCAGACATCCACTTGTCCTGGGCCTGCTGCTGTGTGTGCTGGGCCCAGTG
PapAnu_1A1    (1) ------ATGGCTGTGGAGTCCCAAGGCAGACATCCACTTGTCCTGGGCCTGCTGCTGTGTGTGCTGGGCCCAGTG
BosTau_1A1    (1) ------ATGACAGCAGGGTCCCAGGGGGATCGCCCAGTCATCCTGCTCCTGCTGCTGTGCGCACTCGGCCCCTCC
CanFam_1A1    (1) ------ATGGCAGCAGAGGCCCCGGGGCCACGTCCGCTGGTCCTGGGCCTGCTGCTGTGCGCCTTGAGTGCCCCT
FelCat_1A1    (1) ------ATGGCAGCGAGGTCCCGGGGGCCACGTCCGCTTGTGCTGAGCCTGCTGCTGTGTGCCCTGAATCCCCTT
MusNig_1A1    (1) ---------------------------------------------------------------------------
UrsMar_1A1    (1) ---------------------------------------------------------------------------
UrsThi_1A1    (1) ---------------------------------------------------------------------------
ProLot_1A1    (1) ---------------------------------------------------------------------------
CanRuf_1A1    (1) ---------------------------------------------------------------------------
MusPut_1A1    (1) ---------------------------------------------------------------------------
ChrBra_1A1    (1) ---------------------------------------------------------------------------
VulVul_1A1    (1) ---------------------------------------------------------------------------
ArcBin_1A1    (1) ---------------------------------------------------------------------------
PanTig_1A1    (1) ---------------------------------------------------------------------------
HyaHya_1A1    (1) ---------------------------------------------------------------------------
PhoVit_1A1    (1) ---------------------------------------------------------------------------
CarSer_1A1    (1) ---------------------------------------------------------------------------
AciJub_1A1    (1) ---------------------------------------------------------------------------
PumCo2_1A1    (1) ---------------------------------------------------------------------------
PumCon_1A1    (1) ---------------------------------------------------------------------------
ParBru_1A1    (1) ---------------------------------------------------------------------------
CivCiv_1A1    (1) ---------------------------------------------------------------------------
CroCro_1A1    (1) ---------------------------------------------------------------------------
HerJav_1A1    (1) ---------------------------------------------------------------------------
MirAng_1A1    (1) ---------------------------------------------------------------------------
PriBen_1A1    (1) ---------------------------------------------------------------------------
ProCri_1A1    (1) ---------------------------------------------------------------------------
LeoGeo_1A1    (1) ---------------------------------------------------------------------------
PanUnc_1A1    (1) ---------------------------------------------------------------------------
LynRuf_1A1    (1) ---------------------------------------------------------------------------
CarAur_1A1    (1) ---------------------------------------------------------------------------
ParTem_1A1    (1) ---------------------------------------------------------------------------
CalUrs_1A1    (1) ---------------------------------------------------------------------------
LeoTig_1A1    (1) ---------------------------------------------------------------------------
PanLeo_1A1    (1) ---------------------------------------------------------------------------
LynCan_1A1    (1) ---------------------------------------------------------------------------
LeoWie_1A1    (1) ---------------------------------------------------------------------------
PanOnc_1A1    (1) ---------------------------------------------------------------------------
PhoHoo_1A1    (1) ---------------------------------------------------------------------------
ArcFor_1A1    (1) ---------------------------------------------------------------------------
PanPar_1A1    (1) ---------------------------------------------------------------------------

                   76                                                                       150
MusMus_1A1   (76) GCATCCCATGCTGGGAGGCTGTTAGTGTTCCCTATGGATGGAAGCCACTGGCTGAGTATGCTTGGAGTTATTCAG
RatNor_1A1   (76) GCGTCCCATGCTGGGAAGCTGTTAGTGATCCCCATAGATGGCAGCCACTGGCTGAGTATGCTCGGAGTTATTCAG
GalGal_1A1   (70) TTGGCTGCAGGTGGGAAGCTGCTGGTGGTGTCTGTGGATGGGAGTCCCTGGTTCAGCGTGCTGGAAATGTTAGAA
HomSap_1A1   (70) GTGTCCCATGCTGGGAAGATACTGTTGATCCCAGTGGATGGCAGCCACTGGCTGAGCATGCTTGGGGCCATCCAG
PanTro_1A1   (70) GTGTCCCATGCTGGGAAGATACTGTTGATCCCAGTGGATGGCAGCCACTGGCTGAGCATGCTTGGGGCCATCCAG
MacMul_1A1   (70) CTGTGCCATGCTGGGAAGATGCTGTTGATCCCAGTGGATGGCAGCCACTGGCTGAGCATGCTTGGGACCATCCAG
MacFas_1A1   (70) CTGTGCCATGCTGGGAAGATGCTGTTGATCCCAGTGGATGGCAGCCACTGGCTGAGCATGCTTGGGACCATCCAG
PapAnu_1A1   (70) CTGTGCCATGCTGGGAAGATGCTGTTGATCCCAGTGGATGGCAGCCACTGGCTGAGCATGCTTGGGGCCATCCAG
BosTau_1A1   (70) GTGTCCCAGGGTGGGAAGCTGCTGGTGGTCCCGGTAGATGGCAGCCACTGGCTGAGTTTGGTCGGACCCCTCCAG
CanFam_1A1   (70) GTGTCCCAGGGTGGGAAGCTGCTGTTGATCCCCGTGGATGGCAGCCACTGGCTGAGCATGCTCGGGGTCGTCAAG
FelCat_1A1   (70) CTGTCCCAGGGCGGGAAGCTGTTGTTGGTCCCAATGGATGGCAGCCACTGGCTGAGCTTGTTCGGGGTCATCCAG
MusNig_1A1    (1) ---------------------------------------------CACTGGCTGAGTGGGCTTGGGGTTATTGAG
UrsMar_1A1    (1) ----------------------------------------------------------------------GTCGG
UrsThi_1A1    (1) ----------------------------------------------------------------------GTCGG
ProLot_1A1    (1) -----------------------------------------------------------------------TCGG
CanRuf_1A1    (1) ------------------------------------------------------------------------CAG
MusPut_1A1    (1) ------------------------------------------------------------------------GAG
ChrBra_1A1    (1) -------------------------------------------------------------------------AG
VulVul_1A1    (1) -------------------------------------------------------------------------AG
```

```
ArcBin_1A1     (1) --------------------------------------------------------------------------G
PanTig_1A1     (1) --------------------------------------------------------------------------G
HyaHya_1A1     (1) ---------------------------------------------------------------------------
PhoVit_1A1     (1) ---------------------------------------------------------------------------
CarSer_1A1     (1) ---------------------------------------------------------------------------
AciJub_1A1     (1) ---------------------------------------------------------------------------
PumCo2_1A1     (1) ---------------------------------------------------------------------------
PumCon_1A1     (1) ---------------------------------------------------------------------------
ParBru_1A1     (1) ---------------------------------------------------------------------------
CivCiv_1A1     (1) ---------------------------------------------------------------------------
CroCro_1A1     (1) ---------------------------------------------------------------------------
HerJav_1A1     (1) ---------------------------------------------------------------------------
MirAng_1A1     (1) ---------------------------------------------------------------------------
PriBen_1A1     (1) ---------------------------------------------------------------------------
ProCri_1A1     (1) ---------------------------------------------------------------------------
LeoGeo_1A1     (1) ---------------------------------------------------------------------------
PanUnc_1A1     (1) ---------------------------------------------------------------------------
LynRuf_1A1     (1) ---------------------------------------------------------------------------
CarAur_1A1     (1) ---------------------------------------------------------------------------
ParTem_1A1     (1) ---------------------------------------------------------------------------
CalUrs_1A1     (1) ---------------------------------------------------------------------------
LeoTig_1A1     (1) ---------------------------------------------------------------------------
PanLeo_1A1     (1) ---------------------------------------------------------------------------
LynCan_1A1     (1) ---------------------------------------------------------------------------
LeoWie_1A1     (1) ---------------------------------------------------------------------------
PanOnc_1A1     (1) ---------------------------------------------------------------------------
PhoHoo_1A1     (1) ---------------------------------------------------------------------------
ArcFor_1A1     (1) ---------------------------------------------------------------------------
PanPar_1A1     (1) ---------------------------------------------------------------------------

                   151                                                                     225
MusMus_1A1   (151) CAGCTCCAGCAGAAGGGGCACGAAGTTGTGGTCATAGCACCTGAAGCCTCAATACACATAAAAGAAGGATCATTT
RatNor_1A1   (151) CAGCTCCAGCAAAAGGGGCACGAAGTGGTGGTCATAGCACCTGAAGCTTCGATACACATAAAAGAAGGATCATTT
GalGal_1A1   (145) GTTCTGAAGCAGAAAGGACATGAAATAGTCGTTGTTGCACCTGAAGCCAATTTAAATGTAAAGCCATCAGAGAGT
HomSap_1A1   (145) CAGCTGCAGCAGAGGGGACATGAAATAGTTGTCCTAGCACCTGACGCCTCGTTGTACATCAGAGACGGAGCATTT
PanTro_1A1   (145) CAGCTGCAGCAGAGGGGACATGAAATAGTTGTCCTAGCACCTGACGCCTCGTTGTACATCAGAGACGGAGCATTT
MacMul_1A1   (145) CAGCTGCAGCAGAGGGGACATGAAATAGTTGTCCTAGCACCTGATGCCTCATTGTACATCGGAGAGGGAGCATTT
MacFas_1A1   (145) CAGCTGCAGCAGAGGGGACATGAAATAGTTGTCCTAGCACCTGATGCCTCATTGTACATCAGAGAGGGAGCATTT
PapAnu_1A1   (145) CAGCTGCAGCAGAGGGGACATGAAATAGTTGTCCTAGCACCTGATGCCTCACTGTACATCAGAGAGGGAGCATTT
BosTau_1A1   (145) CCATTGCAGCAGAAGGGACATGACATAGTGGTCCTGGCACCTGACGCCTCCATATACATTAAAGAGGAAGCATTT
CanFam_1A1   (145) CAGTTGCACCAGAGGGGACATGAGGTAGTGGTGATAGCTTCCGAGGCTTCTGTGTACATCAAAGGAGCAGCATTT
FelCat_1A1   (145) CGGCTGCACCAGCGGGGACACGACGTAGTGGTCGTAGCTCCTGAGGCCTCCGTGTACATTAAAGAAGGAGCGTTT
MusNig_1A1    (31) CAGCTGCACCAGAGGGGACATGACATGGTAGTCCTAGCTTCCGAGGCTTCCGTGCACATTAAAGAAGGAGCGTTC
UrsMar_1A1     (6) CAGCTGCACCAGAGGGGACATGACATAGTGGTCCTAGCTCCTGAGGCCTCCGTGCACATCAAAGAAGCAGCATTT
UrsThi_1A1     (6) CAGCTGCACCAGAGGGGACATGACATAGTGGTCCTAGCTCCTGAGGCCTCCGTGCACATCAAAGAAGCAGCATTT
ProLot_1A1     (5) CAGCTGCACCAGAGGGGACATGACATAGTGGTCCTAGCTTCCGAGGCCTCCATGTACATCAAAGAAGGAACATTT
CanRuf_1A1     (4) CAGTTGCACCAGAGGGGACATGAGGTAGTGGTGATAGCTTCCGAGGCTTCTGTGTACATCAAAGGAGCAGCATTT
MusPut_1A1     (4) CAGCTGCACCAGAGGGGACATGACATGGTAGTCCTAGCTTCCGAGGCTTCCGTGCACATTAAAGAAGGAGCGTTC
ChrBra_1A1     (3) CAGTTGCACCAGAGGGGACATGAGGTAGTGGTGATAGCTTCCGAGGCTTCTGTGTACATCAAAGGAGCAGCATTT
VulVul_1A1     (3) CAGTTGCACCAGAGGGGACATGAGGTAGTGGTGATAGCTCCCGAGGCCTCTGTGCACATCAAAGGAGCAGCATTT
ArcBin_1A1     (2) CAGTTGCACCAGCGGGGACATGACATAGTGGTCCTCGCGTCCGAGGCCGCCGTGTACATTAAGGAAAACCCGATG
PanTig_1A1     (2) CGGCTGCACCAGCGGGGACACGACGTAGTGGTCATAGCTCCTGAGGCCTCCGTGTACATTAAAGAAGGAGCGTTT
HyaHya_1A1     (1) ---------------------------------------------------------------------------
PhoVit_1A1     (1) ---------------------------------------------------------------------------
CarSer_1A1     (1) ---------------------------------------------------------------------------
AciJub_1A1     (1) ---------------------------------------------------------------------------
PumCo2_1A1     (1) ---------------------------------------------------------------------------
PumCon_1A1     (1) ---------------------------------------------------------------------------
ParBru_1A1     (1) ---------------------------------------------------------------------------
CivCiv_1A1     (1) ---------------------------------------------------------------------------
CroCro_1A1     (1) ---------------------------------------------------------------------------
HerJav_1A1     (1) ---------------------------------------------------------------------------
MirAng_1A1     (1) ---------------------------------------------------------------------------
PriBen_1A1     (1) ---------------------------------------------------------------------------
ProCri_1A1     (1) ---------------------------------------------------------------------------
LeoGeo_1A1     (1) ---------------------------------------------------------------------------
PanUnc_1A1     (1) ---------------------------------------------------------------------------
LynRuf_1A1     (1) ---------------------------------------------------------------------------
CarAur_1A1     (1) ---------------------------------------------------------------------------
ParTem_1A1     (1) ---------------------------------------------------------------------------
CalUrs_1A1     (1) ---------------------------------------------------------------------------
```

```
LeoTig_1A1     (1) ---------------------------------------------------------------------------
PanLeo_1A1     (1) ---------------------------------------------------------------------------
LynCan_1A1     (1) ---------------------------------------------------------------------------
LeoWie_1A1     (1) ---------------------------------------------------------------------------
PanOnc_1A1     (1) ---------------------------------------------------------------------------
PhoHoo_1A1     (1) ---------------------------------------------------------------------------
ArcFor_1A1     (1) ---------------------------------------------------------------------------
PanPar_1A1     (1) ---------------------------------------------------------------------------

                   226                                                                     300
MusMus_1A1   (226) TACACTCTGAGGAAGTTCCCTGTGCCATTCCAGAAGGAAAATGTGACAGCTACTTTGGTGGAACTTGGACGGACT
RatNor_1A1   (226) TACACTATGAGGAAGTACCCTGTGCCATTCCAAAATGAAAACGTGACAGCTGCTTTTGTGGAACTTGGGCGGAGT
GalGal_1A1   (220) TTTATTTTGAAAACCTACCCAGCCTCTTTCACACAGGAAGAGATGGATGACAATTTCCAGGCATTTCTTAAGGAT
HomSap_1A1   (220) TACACCTTGAAGACGTACCCTGTGCCATTCCAAAGGGAGGATGTGAAAGAGTCTTTTGTTAGTCTCGGGCATAAT
PanTro_1A1   (220) TACACCTTGAAGACGTACCCTGTGCCATTCCAAAGGGAGGATGTGAAAGAGTCTTTTGTTAGTCTCGGGCATAAT
MacMul_1A1   (220) TACACCTTGAAGACGTACCCTGTGCCATTCCAAAGGGAGGATGTGAAAGAGTCTTTTGTTAGTCTTGGGCATAAT
MacFas_1A1   (220) TACACCTTGAAGACGTACCCTGTGCCATTCCAAAGGGAGGATGTGAAAGAGTCTTTTGTTAGTCTTGGGCATAAT
PapAnu_1A1   (220) TACACCTTGAAGACGTACCCTGTGCCATTCCAAAGGGAGGATGTGAAAGAGTCTTTTGTTAGTCTTGGGCATAAT
BosTau_1A1   (220) TACACCTTGAAGAGGTACCCTGTGCCATTCCGAAGGGAGGACTTGGAAGAGACTTTTATCAGCCTCGGGCGTACT
CanFam_1A1   (220) TACACCCTGAAGAGGTACCCTGTGCCATTCCGAAGGGAGGACGTGGAAGCCACTTTTACCAGCCTCGGGCGCGGT
FelCat_1A1   (220) TACACCTTGAAGAGCTACCCCGTCCCATTCCGGAGGGAGGACGTGGAAGCGTCTTTTACTGGTCTCGGGCTCGGG
MusNig_1A1   (106) TACACCTTGAAGAGGTACCCTGTGCCATTCAGAAGGGAGGACGTGGAAGAAGCTTTTATCAAACTCGGGCACGGT
UrsMar_1A1    (81) TACAGCTTGAAGAGGTACCCTGTGCCATTCCGAAGGGAGGACGTGGAAGCCACTTTTATCAGACTTGGGCACGGT
UrsThi_1A1    (81) TACAGCTTGAAGAGGTACCCTGTGCCATTCCGAAGGGAGGACGTGGAAGCCACTTTTATCAGACTTGGGCACGGT
ProLot_1A1    (80) TACACCTTGAAGAGGTACCCTGTGCCATTCCGAAGGGAGGACATAGAAGGAGCTTTCCTCAAACTTGGGCACGGT
CanRuf_1A1    (79) TACACCCTGAAGAGGTACCCTGTGCCATTCCGAAGGGAGGACGTGGAAGCCACTTTTACCAGCCTCGGGCGCGGT
MusPut_1A1    (79) TACACCTTGAAGAGGTACCCTGTGCCATTCAGAAGGGAGGACGTGGAAGAAGCTTTTATCAAACTCGGGCACGGT
ChrBra_1A1    (78) TACACCCTGAAGAGGTACCCTGTGCCATTCCGAAGGGAGGACGTGGAAGCCACTTTTACCAGCCTCGGGCGCGGT
VulVul_1A1    (78) TACACCCTGAAGAGGTACCCTGTGCCATTCCGAAGGGAGGACGTGGAAGCCACTTTTATCAGCCTCGGGCGCGGT
ArcBin_1A1    (77) TACACCTTGAAGAGCTACCCCGTGCCGTTCCGGAGGGAGGACGTGCAAGAGGCTTTCACCAGTCTTGGGCACAGT
PanTig_1A1    (77) TACACCTTGAAGAGCTACCCCGTCCCATTCCGGAGGGAGGACGTGGAAGCATCTTTTACTGGTCTCGGGCTCGAG
HyaHya_1A1     (1) ---------------TTCCCTGTGCCATTCCGCAAGGAGGATATGGAAGAGGCTTTTATCAGTCTCGGGCATGAT
PhoVit_1A1     (1) ------------------CCTGTGCCATTCCGAAGGGAGGACGTGGAAGCGGCTTTTACCAGACTTGGGCACGGT
CarSer_1A1     (1) ----------------------------------------------AAACGTCTTTTACTGGNCTCGGGCTCGGG
AciJub_1A1     (1) -----------------------------------------------AGCGTCTTTTACTGGTCTCGGGCTCGGG
PumCo2_1A1     (1) -----------------------------------------------AGCGTCTTTTACTGGTCTCGGGCTCGGG
PumCon_1A1     (1) --------------------------------------------------GTCTTTTACTGGTCTCGGGCTCGGG
ParBru_1A1     (1) --------------------------------------------------GGCTTTTATCAGTCTCGGGCATAAT
CivCiv_1A1     (1) ----------------------------------------------------CTTTTGTCAGGCTTGGTTACAGT
CroCro_1A1     (1) ------------------------------------------------------TTTATCAGTCTCGGGCATAAT
HerJav_1A1     (1) ----------------------------------------------------------TCGGTCTCGGACTTGCT
MirAng_1A1     (1) -----------------------------------------------------------CAGACTTGGGCACGGT
PriBen_1A1     (1) -----------------------------------------------------------GGGTCTCGGGCTCGAG
ProCri_1A1     (1) -------------------------------------------------------------GTCTCGGGCATAAT
LeoGeo_1A1     (1) --------------------------------------------------------------TCTCGGGCTCGAG
PanUnc_1A1     (1) ---------------------------------------------------------------CTTGGGCTCGAG
LynRuf_1A1     (1) ----------------------------------------------------------------TCGGGCTCGGG
CarAur_1A1     (1) ------------------------------------------------------------------GGGCTCGGG
ParTem_1A1     (1) ---------------------------------------------------------------------CTCGAG
CalUrs_1A1     (1) -----------------------------------------------------------------------CGGT
LeoTig_1A1     (1) -------------------------------------------------------------------------GG
PanLeo_1A1     (1) -------------------------------------------------------------------------AG
LynCan_1A1     (1) --------------------------------------------------------------------------T
LeoWie_1A1     (1) --------------------------------------------------------------------------G
PanOnc_1A1     (1) ---------------------------------------------------------------------------
PhoHoo_1A1     (1) ---------------------------------------------------------------------------
ArcFor_1A1     (1) ---------------------------------------------------------------------------
PanPar_1A1     (1) ---------------------------------------------------------------------------

                   301                                                                     375
MusMus_1A1   (301) GCCTTTAATCAAGATTCTTTTCTGCTGCGCGTGGTCAAAATATATATGAAAGTCAAAAGGGATTCCAGTATGCTC
RatNor_1A1   (301) GTCTTTGATCAAGATCCTTTTCTGCTGCGTGTGGTTAAAACATACAACAAAGTCAAAAGGGACTCCAGTATGCTG
GalGal_1A1   (295) GCATTTGAAGAAGGATCGTTTTTGGAACAATTTCATAGACTGCAAGAAAAAGTAAAAAGACTCTTTGATGTAGGT
HomSap_1A1   (295) GTTTTTGAGAATGATTCTTTCCTGCAGCGTGTGATCAAAACATACAAGAAAATAAAAAAGGACTCTGCTATGCTT
PanTro_1A1   (295) GTTTTTGAGAATGATTCTTTCCTGCAGCGTGTGATCAAAACATACAAGAAAATAAAAAAGGACTCTGCTATGCTT
MacMul_1A1   (295) GTTTTTGAGAATGATTCTTTCCTGCAGCGTGTGATCAAAACATACAAGAAAATAAAAAAGGACTCTGCTATGCTT
MacFas_1A1   (295) GTTTTTGAGAATGATTCTTTCCTGCGGCGTGTGATCAAAACATACAAGAAAATAAAAAAGGACTCTGCTATGCTT
PapAnu_1A1   (295) GTTTTTGAGGATGATTCTTTCCTGCGGCGTGTGATCAAAACATACAAGAAAATAAAAAAGGACTCTGCTATGCTT
BosTau_1A1   (295) GTTTTTGAGGATGATCCTTTCCTGAAGCGCGTGATCAAAACCTACCAGAAAATCAAAAAGGTCTCGGCTCTGCTC
CanFam_1A1   (295) GTTTTTGAGAATGTGCCTTTGCTGCGGCGTGTGATCAAAACGTACAAGAAGGTCAAGGAGGACTCGGCTCTGCTT
FelCat_1A1   (295) GTTTTTGAGAAGAAGCCTTTCCTGCAGCGTGTGGTCGCGACGTACAAGAGGGTCAAGAAGGACTCTGCTCTGCTT
```

```
MusNig_1A1  (181) GTCTTTGAGAATGAGCCTTTGCTGCAGCGTGTGGTCAAAACGTACAAGAAAGTCAAGGAGGACTCTGCCCTTATT
UrsMar_1A1  (156) GTTTTTGAGAATGACTCTTTGCTGCAGCGTGTGGTCAAAACGTACAAGAGAGTCAAGGAGGACTCTGCCCTGATT
UrsThi_1A1  (156) GTTTTTGAGAATGACTCTTTGCTGCAGCGTGTGGTCAAAACGTACAAGAGAGTCAAGGAGGACTCTGCCCTGATT
ProLot_1A1  (155) GTCTTTGAGAATGAGCCTTTGCTGCAGCGTGTGGTCAAAACGTACAAGAAAGTCAAGAAGGACTCTGCCCTGCTT
CanRuf_1A1  (154) GTTTTTGAGAATGTGCCTTTGCTGCGGCGTGTGATCAAAACGTACAAGAAGGTCAAGGAGGACTCGGCTCTGCTT
MusPut_1A1  (154) GTCTTTGAGAATGAGCCTTTGCTGCAGCGTGTGGTCAAAACGTACAAGAAAGTCAAGGAGGACTCTGCCCTGATT
ChrBra_1A1  (153) GTTTTTGAGAATGTGCCTTTGCTGCGGCGTGTGATCAAAACGTACAAGAAGGTCAAGGAGGACTCGGCTCTGCTT
VulVul_1A1  (153) GTTTTTGAGAATGTGCCTTTGCTGCGGCGTGTGATCAAAACGTACAAGAAGGTCAAGGAGGACTCGGCTCTGCTT
ArcBin_1A1  (152) GTTTTCGAGAACAAGCCTTTCCTGCAGCGTGCGGTCCAGACCTACAAGAAGGTCAAGAAGGACTCTGCTCTGCTC
PanTig_1A1  (152) GTTTTTGAGAAGAAGCCTTTCCTGCAGCGTGTGGTCGAGACATACAAGAGGGTCAAGAAGGACTCTGCTCTGCTT
HyaHya_1A1   (61) GTTTTTGAAAAGAAACCTTTCCTGCAGTATGCAGTCCAGATATACAAGAAAATCAAGAAGGACTCTGCTCTGCTT
PhoVit_1A1   (58) GTTTTTGAGAAAGAGCCTTTGCTGCAGCGTGTGGTCAAAACGTATAAGAAAGTCAAGGAGGACTCTGCCCTGCTT
CarSer_1A1   (30) GTTTTTGAGAAGAAGCCTTTCCTGCAGCGTGTGGTCGAGACATACAAGAGGGTCAAGAAGGACTCTGCTCTGCTT
AciJub_1A1   (29) GTTTTTGAGAAGAAGCCTTTCCTGCAGCGTGTGGTCGAGACATACAAGAGGGTCAAGAAGGACTCTGCTCTGCTT
PumCo2_1A1   (29) GTTTTTGAGAAGAAGCCTTTCCTGCAGCGTGTGGTCGAGACATACAAGAGGGTCAAGAAGGACTCTGCTCTGCTT
PumCon_1A1   (26) GTTTTTGAGAAGAAGCCTTTCCTGCAGCGTGTGGTCGAGACATACAAGAGGGTCAAGAAGGACTCTGCTCTGCTT
ParBru_1A1   (26) GTTTTTGAAAAGAAACCTTTCCTGCAGTATGCAGTCCAGATATACAAGAAAATCAAGAAGGACTCTGCTCTGCTT
CivCiv_1A1   (24) GTTTTTGAGAAGAAGCCTTTTCTGCAGCGTGTGGTCCAGACATACAAGAATGTACAGAAGAGCTCCGCTCTGCTT
CroCro_1A1   (22) GTTTTTGAAAAGAAACCTTTCCTGCAGTATGCAGTCCAGATATACAAGAAAATCAAGAAGGACTCTGCTCTGCTT
HerJav_1A1   (18) GTTTTTGAGAAGAAACCTCTCCTGCAGTACGTGGTCCAGACGTACAAGAAAATCCAGAAGGACTCTGCTCTGCTG
MirAng_1A1   (17) GTTTTTGAGAAAGAGCCTTTGCTGCAGCGTGTGGTCAAAACGTATAAGAAAGTCAAGGAGGACTCTGCCCTGCTT
PriBen_1A1   (17) GTTTTTGAGAAGAAGCCTTTCCTGCAGCGTGTGGTCAAGACATACAAGAGGGTCAAGAAGGACTCTGCTCTGCTT
ProCri_1A1   (15) GTTTTTGAGAAGAAACCTTTCCTGCAGTATGCAGTCCAGATATACAAGAGGATCAAGAAGGACTCTGCTCTGCTT
LeoGeo_1A1   (14) GTTTTTGAGAAGAAGCCTTTCCTGCAGCGTGTGGTCAAGACATATAAGAGGGTCAAGAAGGACTCTGCTCTGCTT
PanUnc_1A1   (13) GTTTTTGGGAAGAAGCCTTTCCTGCAGCGTGTGGTCGAGACATACAAGAGGGTCAAGAAGGACTCTGCTCTGCTT
LynRuf_1A1   (12) GTTTTTGAGAAGAAGCCTTTCCTGCAGCGTGTGGTCGAGACATACAAGAGGGTCAAGAAGGACTCTGCTCTGCTT
CarAur_1A1   (10) GTTTTTGAGAAGAAGCCTTTCCTGCAGCGTGTGGTCGAGACATACAAGAGGGTCAAGAAGGACTCTGCTCTGCTT
ParTem_1A1    (7) GTTTTTGAGAAGAAGCCTTTCCTGCAGCGTGTGGTCGAAACATACAAGAGGGTCAAGAAGGACTCTGCTCTGCTT
CalUrs_1A1    (5) GTTTTTGATGATGAGCCTTTGCTGCAGCGTGTGGTCAAAATGTATAAGAAAGTCAAGGAGGACTCTGCCCTGCTT
LeoTig_1A1    (3) TTTTTNGAGAAGAAGCCTTTCCTGCAGCGTGTGGTCAAGACATATAAGAGGGTCAAGAAGGACTCTGCTCTGCTT
PanLeo_1A1    (3) GTTTTTGAGAAGAAGCCTTTCCTGCAGCGTGTGGTCGAGACATACAAGAGGGTCAAGAAGGACTCTGCTCTGCTT
LynCan_1A1    (2) GTTTTTGAGAAGAAGCCTTTCCTGCAGCGTGTGGTCAAGACATACAAGAAGGTCAAGAAGGACTCTGCCCTGCTT
LeoWie_1A1    (2) GTTTTTGAGAAGAAGCCTTTCCTGCAGCGTGTGGTCAAGACATATAAGAGGGTCAAGAAGGACTCTGCTCTGCTT
PanOnc_1A1    (1) -----TGAGAAGAAGCCTTTCCTGCAGCGTGTGGTCGAGACATACAAGAGGGTCAAGAAGGACTCTGCTCTGCTT
PhoHoo_1A1    (1) --------GAATGAGCCTTTGCTGCAGCGTGTGGTCAAAATGTATAAGAAAGTCAAGGAGGACTCTGCCCTGCTT
ArcFor_1A1    (1) ---------AATGAGCCTTTGCTGCAGCGTGTGGTCAAAATGTATAAGAAAGTCAAGGAGGACTCTGCCCTGCTT
PanPar_1A1    (1) -----------GAAGCCTTTCCTGCAGCGTGTGGTCGAGACATACAAGAGGGTCAAGAAGGACTCTGCTCTGCTT

                  376                                                                      450
MusMus_1A1  (376) CTAGCTGGCTGCTCCCACCTGCTGCACAATGCCGAGTTTATGGCCTCTCTGGAAGAAAGTCACTTTGATGCTCTG
RatNor_1A1  (376) CTGTCTGGCTGCTCCCACCTTCTGCACAATGCCGAGTTTATGGCCTCTCTGGAACAAAGCCACTTTGATGCTCTG
GalGal_1A1  (370) TTCATATCCTGTGCATGGTTACTGAAGAACAAAGAGCTTATCAGATATCTTGAGGAAAGTAACTTTGATGCTCTC
HomSap_1A1  (370) TTGTCTGGCTGTTCCCACTTACTGCACAACAAGGAGCTCATGGCCTCCCTGGCAGAAAGCAGCTTTGATGTCATG
PanTro_1A1  (370) TTGTCTGGCTGTTCCCACTTACTGCACAACAAGGAGCTCATGGCCTCCCTGGCAGAAAGCAGCTTTGATGTCATG
MacMul_1A1  (370) TTGTCTGGCTGTTCCCACTTACTGCACAACAAGGAGCTCATGGCCTCCCTTGCGGAAAGCAGCTTTGACGTCATG
MacFas_1A1  (370) TTGTCTGGCTGTTCCCACTTACTGCACAACAAGGAGCTCATGGCCTCCCTTGCGGAAAGCAGCTTTGACGTCATG
PapAnu_1A1  (370) TTGTCTGGCTGTTCCCACTTACTGCACAACAAGGAGCTCATGGCCTCCCTTGCGGAAAGCAGCTTTGACGTCATG
BosTau_1A1  (370) TTATCCGCCTGCTCCCATTTACTGCACAACAAGGAGCTGATGGCCTCCCTGACGGCAAGTAGCTTCGATGCCGTG
CanFam_1A1  (370) TTGTCTGCTTGCTCCCATTTACTGCATAACAAGGAGCTGATGGCCTCGCTGGCAGAGAGCAGCTTCGATGCTGTG
FelCat_1A1  (370) TTGTCTGCCTGCTCCCACTTACTGTACAACGAGGAGCTGATGGCCTCCCTGGCGGAAAGCGGCTTCGATGCCATG
MusNig_1A1  (256) TTTTCTGCCTGCTCCCACTTATTGCACAACAAGGAACTTATGGCCTCCCTGGTGGAAAGCCGCTTCGATGCCGTG
UrsMar_1A1  (231) TTGTCTGCCTGCTCCCACTTACTGCACAACAAGGAGCTCATGGCCTCCCTGGTGGAAAGCAGCTTCGATGCCGTG
UrsThi_1A1  (231) TTGTCTGCCTGCTCCCACTTACTGCACAACAAGGAGCTCATGGCCTCCCTGGTGGAAAGCAGCTTCGATGCCGTG
ProLot_1A1  (230) TTTTCTGCCTGCTCCCACTTACTGCACAACAAGGAGCTTATGGCCTCCCTGGTGGAAAGCAGCTTTGATGCCGTG
CanRuf_1A1  (229) TTGTCTGCTTGCTCCCATTTACTGCATAACAAGGAGCTGATGGCCTCGCTGGCAGAGAGCAGCTTCGATGCTGTG
MusPut_1A1  (229) TTTTCTGCCTGCTCCCACTTATTGCACAACAAGGAACTTATGGCCTCCCTGGTGGAAAGCCGCTTCGATGCCGTG
ChrBra_1A1  (228) TTGTCTGCTTGCTCCCATTTACTGCATAACAAGGAGCTGATGGCCTCGCTGGCAGAGAGCAGCTTCGATGCTGTG
VulVul_1A1  (228) TTGTCTGCTTGCTCCCATTTACTGCATAACAAGGAGCTGATGGCCTCGCTGGCAGAAAGCAGCTTCGATGCTGTG
ArcBin_1A1  (227) TTCTCTGCCTGCTCCCACCTGCTGCACAACAAGGAGCTGATGGCCTCCCTGGCGGAAAGCCGCTTCGACGCCGTG
PanTig_1A1  (227) TTGTCTGCCTGCTCCCACTTACTGTACAACAAGGAGCTGATGGCCTCCCTGGCGGAAAGCGGCTTCGATGCCATG
HyaHya_1A1  (136) TTTTCTGCCTGCTCCCACCTACTGCACAACCAGGAGCTGATGGCCTCCCTGGTAGAAAGCGGCTTCGATGCCGTG
PhoVit_1A1  (133) TTGTCTGCCTGCTCCCACTTACTGCACAACAAGGAGCTAATGGCCTCGCTCGCGGAAAGCAACTTCGATGCTGTG
CarSer_1A1  (105) TTGTCTGCCTGCTCCCACTTACTGTACAACGAGGAGCTGATGGCCTCCCTGGCGGAAAGCGGCTTCGATGCCATG
AciJub_1A1  (104) TTGTCTGCCTGCTCCCACTTACTGTACAACGAGGAGCTGATGGCCTCCCTGGTGGAAAGCGGCTTCGATGCCATG
PumCo2_1A1  (104) TTGTCTGCCTGCTCCCACTTACTGTACAACGAGGAGCTGATGGCCTCCCTGGCGGAAAGCGGCTTCGATGCCGTG
PumCon_1A1  (101) TTGTCTGCCTGCTCCCACTTACTGTACAACGAGGAGCTGATGGCCTCCCTGGCGGAAAGCGGCTTCGATGCCGTG
ParBru_1A1  (101) TTTTCTGCCTGCTCCCACCTACTGCACAACCAGGAGCTGATGGCCTCCCTGGTAGAAAGCGGCTTCGATGCCGTG
CivCiv_1A1   (99) TTCTCTGCCTGCTCCCACTTGCTGCACAACAAGGAGCTGATGGCCTCCCTGGCGGAAAGCCACTTCGATGCCGTG
CroCro_1A1   (97) TTTTCTGCCTGCTCCCACCTACTGCACAACCAGGAGCTGATGGCCTCCCTGGCGGAAAGCGGCTTCGATGCCGTG
HerJav_1A1   (93) TTTTCGGCCTGCTCCCACCTCCTGCACAACGAGGAGCTGATGGCTTCCCTGGCGGAAGGCAGCTTCGACGCCGTG
MirAng_1A1   (92) TTGTCTGCCTGCTCCCACTTACTGCACAACAAGGAGCTAATGGCCTCGCTCGCGGAAAGCAACTTCGATGCTGTG
```

```
PriBen_1A1   (92) TTGTCTGCCTGCTCCCACTTGCTGTACGACGAGGAACTGATGGCCTCCCTGGCGGAAAGCGGCTTCGATGCCGTG
ProCri_1A1   (90) TTTTCTGCCTGCTCCCACCTACTGCACAACCAGGAGCTGATGGCCTCCCTGGCGGGAAGCGGCTTCGATGCCGTG
LeoGeo_1A1   (89) TTGTCTGCCTGCTCCCACTTACTGTACAACGAGGAGCTGATGGCCTCCCTGGCGGAAAGCGGCTTCGATGCCATG
PanUnc_1A1   (88) TTGTCTGCCTGCTCCCACTTACTGTACAACAAGGAGCTGATGGCCTCCCTGGCGGAAAGCGGCTTCGATGCCATG
LynRuf_1A1   (87) TTGTCTGCCTGCTCCCACTTACTGTACAACGAGGAGCTGATGGCCTCCCTGGCGGAAAGCGGCCTCGATGCCATG
CarAur_1A1   (85) TTGTCTGCCTGCTCCCACTTACTGTACAACGAGGAGCTGATGGCCTCCCTGGCGGAAAGCGGCTTCGATGCCATG
ParTem_1A1   (82) TTGTCTGCCTGCTCCCACTTACTGTACAACGAGGAGCTGATGGCCTCCCTGGCGGAAAGCGGCTTCGATGCCATG
CalUrs_1A1   (80) TTGTCTGCCTGCTCCCACTTACTGCACAACAAGGAGCTAATGGCCTCGCTGGTGGAAAGCAGCTTCGATGCCGTG
LeoTig_1A1   (78) TTGTCTGCCTGCTCCCACTTACTGTACAACGAGGAGCTGATGGCCTCCCTGGCGGAAAGCGGCTTCGATGCCATG
PanLeo_1A1   (78) TTGTCTGCCTGCTCCCACTTACTGTACAACAAGGAGCTGATGGCCTCCCTGGCGGAAAGCGGCTTCGATGCCATG
LynCan_1A1   (77) TTGTCTGCCTGCTCCCACTTACTGCACAACAAGGAGCTTATGGCCTCCCTGGGGGAAAGCAGCTTTGATGCCGTG
LeoWie_1A1   (77) TTGTCTGCCTGCTCCCACTTACTGTACAACGAGGAGCTGATGGCCTCCCTGGCGGAAAGCGGCTTCGATGCCATG
PanOnc_1A1   (71) TTGTCTGCCTGCTCCCACTTACTGTACAACAAGGAGCTGATGGCCTCCCTGGCGGAAAGCGGCTTCGATGCCATG
PhoHoo_1A1   (68) TTGTCTGCCTGCTCCCACTTACTGCACAACAAGGAGCTAATGGCCTCGCTGGTGGAAAGCAGCTTCGATGCCGTG
ArcFor_1A1   (67) TTGTCTGCCTGCTCCCACTTACTGCACAACAAGGAGCTAATGGCCTCGCTGGTGGAAAGCAGCTTCGATGCCGTG
PanPar_1A1   (65) TTGTCTGCCTGCTCCCACTTACTGTACAACAAGGAGCTGATGGCCTCCCTGGCGGAAAGCGGCTTCGATGCCATG

                  451                                                                     525
MusMus_1A1  (451) CTGACAGACCCTTTCCTTCCCTGTGGCTCCATTGTGGCCCAGTACCTGACTGTGCCCACTGTGTACTTCTTGAAT
RatNor_1A1  (451) CTGACAGACCCTTTCCTTCCGTGTGGCTCCATTGTGGCCCAGTACCTGTCTCTGCCTGCTGTGTACTTCTTGAAT
GalGal_1A1  (445) TTCACTGACCCTGTGTTTCCTTGTGGAGCGATACTGGCCGAGCATCTTTCCATCCCTTCTGTGTATTTCATGAGG
HomSap_1A1  (445) CTGACGGACCCTTTCCTTCCTTGCAGCCCCATCGTGGCCCAGTACCTGTCTCTGCCCACTGTATTCTTCTTGCAT
PanTro_1A1  (445) CTGACGGACCCTTTCCTTCCTTGCAGCCCCATCGTGGCCCAGTACCTGTCTCTGCCCACTGTATTCTTCTTGCAT
MacMul_1A1  (445) CTGACAGACCCTTTCCTTCCTTGTGGCCCCATCGTGGCCCAGTACCTGTCTCTGCCCACTGTATTCTTCTTGAAT
MacFas_1A1  (445) CTGACAGACCCTTTCCTTCCTTGTGGCCCCATCGTGGCCCAGTACCTGTCTCTGCCCACTGTATTCTTCTTGAAT
PapAnu_1A1  (445) CTGACAGACCCTTTCCTTCCTTGTGGCCCCATCGTGGCCCAGTACCTGTCTCTGCCCACTGTATTCTTCTTGAAT
BosTau_1A1  (445) TTGACAGACCCTTTCCTTCCCTGCGGCCCCATCGTGGCCCAGTACCTGTCTGTGCCTGCCGTGTTCTTCTTGAAT
CanFam_1A1  (445) TTGACGGACCCCTTCCTTCCTTGCGGTCCCATCGTGGCCCTGTACCTGGCCCTCCCTGCTGTGTTCTTCTTGCAC
FelCat_1A1  (445) TTGACAGACCCTTTCCTTCCTTGTGGCCCCATCGTGGCCCTGCGCCTGGCGCTGCCTGTCGTGTTCTTCTTGAAC
MusNig_1A1  (331) TTGGCAGACCCTTTCCTCCCTTGTGGCCCCATCGTGGCGCTGTACCTGGGTCTGCCTGCTGTGTTCTTCTTGAAT
UrsMar_1A1  (306) TTGACAGACCCTTTCCTCCCTTGTGGCCCCATCGTGGCCCTGTACCTGGCTCTGCCTGCCGTGTTCTTCTTGAAT
UrsThi_1A1  (306) TTGACAGACCCTTTCCTCCCTTGTGGCCCCATCGTGGCCCTGTACCTGGCTCTGCCTGCCGTGTTCTTCTTGAAT
ProLot_1A1  (305) TTGACAGACCCTTTGCTCCCTTGTGGCCCCATCGTGGCGCTGTACCTGGCTCTGCCTGCCGTGTTTTTCTTAAAC
CanRuf_1A1  (304) TTGACGGACCCCTTCCTTCCTTGCGGTCCCATCGTGGCCCTGTACCTGGCCCTCCCTGCTGTGTTCTTCTTGCAC
MusPut_1A1  (304) TTGGCAGACCCTTTCCTCCCTTGTGGCCCCATCGTGGCGCTGTACCTGGGTCTGCCTGCTGTGTTCTTCTTGAAT
ChrBra_1A1  (303) TTGACGGACCCCTTCCTTCCTTGCGGTCCCATCGTGGCCCTGTACCTGGCCCTCCCTGCTGTGTTTTTCTTGCAC
VulVul_1A1  (303) TTGACGGACCCCTTCCTTCCTTGCGGTCCCATCGTGGCCCTGTACCTGGCCCTCCCTGCTGTGTTCTTCTTGCAC
ArcBin_1A1  (302) TTGACAGACCTTTTCATCCCCTGCGGCCCCATCGTGGCCCTGCACCTGGCGCTGCCTGCTGTGTTCTTCTTGAAC
PanTig_1A1  (302) TTGACAGACCCTTTCCTTCCTTGTGGCCCCATCGTGGCCCTGCGCCTGGCACTGCCTGTCGTGTTCTTCTTGAAC
HyaHya_1A1  (211) TTGACAGACCCTTTCCTTCCCTGCGGCCCCATCGTGGCCCTGTACCTGGAGCTGCCCGCCGTGTTCTTCTCAAAC
PhoVit_1A1  (208) TTGACAGACCCTTTCCTCCCTTGTGGCCCCATCGTGGCCCTGTACCTGGCTCTGCCTGCCGTGTTCTTCTTGAAT
CarSer_1A1  (180) TTGACAGACCCTTTCCTTCCTTGTGGCCCCATCGTGGCCCTGCGCCTGGCCCTGCTGGTCGTGTTCTTCTTGAAC
AciJub_1A1  (179) TTGACAGACCCTTTCCTTCCTTGTGGCCCCATCGTGGCCCTGCGCCTGGCACTGCCTGTCGTGTTCTTCTTGCAC
PumCo2_1A1  (179) TTGACAGACCCTTTCCTTCCTTGTGGCCCCATCGTGGCCCTGCGCCTGGCACTGCCTGTCGTGTTCTTCTTGCAC
PumCon_1A1  (176) TTGACAGACCCTTTCCTTCCTTGTGGCCCCATCGTGGCCCTGCGCCTGGCACTGCCTGTCGTGTTCTTCTTGCAC
ParBru_1A1  (176) TTGACAGACCCTTTCCTTCCCTGCGGCCCCATCGTGGCCCTGTACCTGGAGCTGCCCGCCGTGTTCTTCTCAAAC
CivCiv_1A1  (174) TTGACAGACCCTTTCCTTCCCTGCGGCCCCATCGTGGCCCTGCACCTGGCGCTGCCTGCTGTGTTCTTCTTGAAC
CroCro_1A1  (172) TTGACAGACCCTTTCCTTCCCTGCGGCCCCATCGTGGCCCTGTACCTGGAGCTGCCCGCCGTGTTCTTCTCGAAC
HerJav_1A1  (168) CTGACGGACCCTTTCCTCCCCTGCGGCCCCATCGTGGCCCTGCGCCTGGCGCTGCCCGTTGTGTTCTTCTCCCAC
MirAng_1A1  (167) TTGACAGACCCTTTCCTCCCTTGTGGCCCCATCGTGGCCCTGTACCTGGCTCTGCCTGCCGTGTTCTTCTTGAAT
PriBen_1A1  (167) TTGACAGACCCTTTCCTTCCTTGTGGCCCCATCGTGGCCCTGCGCCTGGCACTGCCTGTCGTGTTCTTCTTGAAC
ProCri_1A1  (165) TTGACAGACCCTTTCCTTCCCTGCGGCCCCATCGTGGCCCTGTACCTGGAGCTGCCCGCCGTGTTCTTCTCGAAC
LeoGeo_1A1  (164) TTGACAGACCCTTTCCTTCCTTGTGGCCCCATCGTGGCCCTGCGCCTGGCACTGCCTGTCGTGTTCTTCTTGAAC
PanUnc_1A1  (163) TTGACAGACCCTTTCCTTCCTTGTGGCCCCATCGTGGCCCTGCGCCTGGCACTGCCTGTCGTGTTCTTCTTGAAC
LynRuf_1A1  (162) TTGACAGACCCTTTCCTTCCTTGTGGCCCCATCGTGGCCCTGCGCCTGGCACTGCCTGTCGTGTTCTTCTTGCAC
CarAur_1A1  (160) TTGACAGACCCTTTCCTTCCTTGTGGCCCCATCGTGGCCCTGCGCCTGGCACTGCCTGTCGTGTTCTTCTTGAAC
ParTem_1A1  (157) TTGACAGACCCTTTCCTTCCTTGTGGCCCCATCGTGGCCCTGCGCCTGGCGCTGCCTGTCGTGTTCTTCTTGAAC
CalUrs_1A1  (155) TTGACAGACCCTTTCCTCCCTTGTGGCCCCATCGTGGCCCTGTACCTGGGTCTGCCTGCCGTGTTCTTCTTGAAT
LeoTig_1A1  (153) TTGACAGACCCTTTCCTTCCTTGTGGCCCCATCGTGGCCCTGCGCCTGGCACTGCCTGTCGTGTTCTTCTTGAAC
PanLeo_1A1  (153) TTGACAGACCCTTTCCTTCCTTGTGGCCCCATCGTGGCCCTGCGCCTGGCACTGCCTGTCGTGTTCTTCTTGAAC
LynCan_1A1  (152) TTGACAGACCCTTTCCTTCCTTGTGGCCCCATCGTGGCCCTGTACCTGGCTCTGCCTGTCGTGTTTTTCTTGCAC
LeoWie_1A1  (152) TTGACAGACCCTTTCCTTCCTTGTGGCCCCATCGTGGCCCTGCGCCTGGCACTGCCTGTCGTGTTCTTCTTGAAC
PanOnc_1A1  (146) TTGACAGACCCTTTCCTTCCTTGTGGCCCCATCGTGGCCCTGCGCCTGGCACTGCCTGTCGTGTTCTTCTTGAAC
PhoHoo_1A1  (143) TTGACAGACCCTTTCCTCCCTTGTGGCCCCATCGTGGCCCTGTACCTGGGTCTGCCTGCCGTGTTCTTCTTGAAT
ArcFor_1A1  (142) TTGACAGACCCTTTCCTCCCTTGTGGCCCCATCGTGGCCCTGTACCTGGGTCTGCCTGCCGTGTTCTTCTTGAAT
PanPar_1A1  (140) TTGACAGACCCTTTCCTTCCTTGTGGCCCCATCGTGGCCCTGCGCCTGGCACTGCCTGTCGTGTTCTTCTTGAAC

                  526                                                                     600
MusMus_1A1  (526) AAATTGCCATGCAGCCTGGATTCAGAAGCTACCCAATGCCCCGTCCCATTGTCCTACGTGCCCAAGAGTTTGTCT
RatNor_1A1  (526) GCATTGCCATGCAGCCTGGATTTGGAAGCCACCCAATGCCCTGCTCCGTTGTCCTACGTGCCCAAGAGTTTGTCC
GalGal_1A1  (520) GGAATGCCATGTGGATTAGACTTTGAGGCTACTCAATGTCCCAATCCCCCTTCTTATATCCCTAGGGCATTTACA
```

```
HomSap_1A1  (520) GCACTGCCATGCAGCCTGGAATTTGAGGCTACCCAGTGCCCCAACCCATTCTCCTACGTGCCCAGGCCTCTCTCC
PanTro_1A1  (520) GCACTGCCATGCAGCCTGGAATTTGAGGCTACCCAGTGCCCCAACCCATTCTCCTACGTGCCCAGGCCTCTCTCC
MacMul_1A1  (520) GCATTGCCATGCAGCCTGGAATCTGAGGCTACCCAGTGCCCCAACCCATTCTCCTACGTGCCCAGGCCTCTGTCC
MacFas_1A1  (520) GCATTGCCATGCAGCCTGGAATCTGAGGCTACCCAGTGCCCCAACCCATTCTCCTACGTGCCCAGGCCTCTGTCC
PapAnu_1A1  (520) GCATTGCCATGCAGCCTGGAATTTGAGGCTACCCAGTGCCCCAACCCATTCTCCTACGTGCCCAGGCCTCTGTCC
BosTau_1A1  (520) GGACTGCCATGCAGCCTGGACTTTCAGGGTACCCAGAGCCCCAGTCCACCATCCTACGTGCCCAGGTATCTGTCC
CanFam_1A1  (520) GCACTGCCATGCAGCCTAGATTTTCAAGGTACCCAGTGCCCCAACCCACCATCCTATGTGCCCAGGGCTCTGTCC
FelCat_1A1  (520) TCGCTGCCCTGCGGCCTAGATTTTCAAGGTACCCGCTGCCCCAGCCCACCATCCTATGTGCCCAGGGTTCTGTCC
MusNig_1A1  (406) GCGCTGCCGTGTGGCCTAGATTTTCAAGGTACTCAGTGCCCCAACCCACCATCTTATGTGCCCAGGGCTCTGTCC
UrsMar_1A1  (381) GCGCTGCCGTGTGGCCTAGATTCTCAAGGTACCCAGTGCCCCAACCCACCATCCTATGTGCCCAGGCCTCTCTCC
UrsThi_1A1  (381) GCGCTGCCGTGTGGCCTAGATTCTCAAGGTACCCAGTGCCCCAACCCACCATCCTATGTGCCCAGGCCTCTCTCC
ProLot_1A1  (380) TCGCTACCGTGTGGCCTGGATTTTCAAGGTACCCAGTGCCCCAGCCCACCATCTTATGTGCCCAGGGCTCTGTCC
CanRuf_1A1  (379) GCACTGCCATGCAGCCTAGATTTTCAAGGTACCCAGTGCCCCAACCCACCATCCTATGTGCCCAGGGCTCTGTCC
MusPut_1A1  (379) GCGCTGCCGTGTGGCCTAGATTTTCAAGGTACTCAGTGCCCCAACCCACCATCTTATGTGCCCAGGGCTCTGTCC
ChrBra_1A1  (378) GCACTGCCATGCAGCCTAGATTTTCAAGGTACCCAGTGCCCCAGCCCACCATCCTATGTGCCCAGGGCTCTGTCC
VulVul_1A1  (378) GCACTGCCATGCAGCCTAGATTTTCAAGGTACCCAGTGCCCCAACCCACCATCCTATGTGCCCAGGACTCTGTCC
ArcBin_1A1  (377) GCGCTGCCGTGTGGAGTCGATTTTCAAGGTGCCCATTGCCCCAGCCCCCCGTCCTACGTGCCCCGGGGCATGTCC
PanTig_1A1  (377) TCACTGCCATGCGGCCTAGATTTTCAAGGTACCCGCTGTCCCAGCCCACCATCCTATGTGCCCAGGGTTCTGTCC
HyaHya_1A1  (286) GCACTGCCATGCGGCCTAGATTTGCAAGGTACCCATTGCCCCAGCCCACCATCCTATGTGCCCAGGGTTCTGTCC
PhoVit_1A1  (283) GCGCTGCCGTGTGGCCTAGATTTTCAAGGTACCCAGTGTCCCAGCCCACCATCCTATGTGCCCAGGGCTCTGTCC
CarSer_1A1  (255) TCACTGCCATGCGGCCTAGATTTTCAAGGTACCCGCTGCCCCAGCCCACCATCCTATGTGCCCAGGGTTCTGTCC
AciJub_1A1  (254) TCACTGCCATGCGGCCTAGATTTTCAAGGTACCCGCTGCCCCAGCCCACCATCCTATGTGCCCAGGGTTCTGTCC
PumCo2_1A1  (254) TCACTGCCATGCGGCCTAGATTTTCAAGGTACCCGCTGCCCCAGCCCACCATCCTATGTGCCCAGGGTTCTGTCC
PumCon_1A1  (251) TCACTGCCATGCGGCCTAGATTTTCAAGGTACCCGCTGCCCCAGCCCACCATCCTATGTGCCCAGGGTTCTGTCC
ParBru_1A1  (251) GCACTGCCATGCGGCCTAGATTTGCAAGGTACCCATTGCCCCAGCCCACCATCCTATGTGCCCAGGGTTCTGTCC
CivCiv_1A1  (249) GTGCTGCCATGTGGCCTAGATTTTCAAGGTACCCATTGCCCCAGCCCACCATCCTATGTGCCCAGGAGTCTGTCC
CroCro_1A1  (247) GCACTGCCATGCGGCCTAGATTTGCAAGGTACCCATTGCCCCAGCCCACCATCCTATGTGCCCAGGGTTCTGTCC
HerJav_1A1  (243) AAACTGCCGTGTGGCCTAGATTTCCAAGGTACCCAATGTCCCAGCCCACCGTCCTACGTGCCCAGGCCTCTGTCC
MirAng_1A1  (242) GCGCTGCCGTGTGGCCTAGATTTTCAAGGTACCCAGTGTCCCAACCCACCATCCTATGTGCCCAGGGCTCTGTCC
PriBen_1A1  (242) TCACTGCCATGCGGCCTAGATTTTCAAGGTACCCGCTGCCCCAGCCCACCATCCTATGTGCCCAGGGTTCTGTCC
ProCri_1A1  (240) GCGCTGCCATGCGGCCTAGATTTGCAAGGTACCCATTGCCCCAGCCCACCATCCTACGTGCCCAGGGTTCTGTCC
LeoGeo_1A1  (239) TCACTGCCATGCGGCCTAGATTTTCAAGGTACCCGCTGCCCCAGCCCACCATCCTATGTGCCCAGGGTTCTGTCC
PanUnc_1A1  (238) TCACTGCCATGCGGCCTAGATTTTCAAGGTACCCGCTGTCCCAGCCCACCATCCTATGTGCCCAGGGTTCTGTCC
LynRuf_1A1  (237) TCACTGCCATGCGGCCTAGATTTTCAAGGTACCCGCTGCCCCAGCCCACCATCCTATGTGCCCAGGGTTCTGTCC
CarAur_1A1  (235) TCACTGCCATGCGGCCTAGATTTTCAAGGTACCCGCTGCCCCAGCCCACCATCCTATGTGCCCAGGGTTCTGTCC
ParTem_1A1  (232) TCACTGCCATGCGGCCTAAATTCTCAAGGTACCCGCTGCCCCAGCCCACCATCCTATGTGCCCAGGGTTCTGTCC
CalUrs_1A1  (230) GCGCTGCCGTGTGGCCTAGATTTTCAAGGTACCCTGTGTCCCAACCCACCATCCTATGTGCCCAGGGCTCTGTCC
LeoTig_1A1  (228) TCACTGCCATGCGGCCTAGATTTTCAAGGTACCCGCTGCCCCAGCCCACCATCCTATGTGCCCAGGGTTCTGTCC
PanLeo_1A1  (228) TCACTGCCATGTGGCCTAGATTTTCAAGGTACCCGCTGTCCCAGCCCACCATCCTATGTGCCCAGGGTTCTGTCC
LynCan_1A1  (227) TCACTGCCATGTGGCCTGGATTTTCAAGGTACCCGGTGCCCCAGCCCACCATCTTATGTGCCCAGGGCTCTGTCC
LeoWie_1A1  (227) TCACTGCCGTGCGGCCTAGATTTTCAAGGTACCCTCTGCCCCAGCCCACCATCCTATGTGCCCAGGGTTCTGTCC
PanOnc_1A1  (221) TCACTGCCATGCGGCCTAGATTTTCAAGGTACCCGCTGTCCCAGCCCACCATCCTATGTGCCCAGGGTTCTGTCC
PhoHoo_1A1  (218) GCGCTGCCGTGTGGCCTAGATTTTCAAGGTACCCTGTGTCCCAACCCACCATCCTATGTGCCCAGGGCTCTGTCC
ArcFor_1A1  (217) GCGCTGCCGTGTGGCCTAGATTTTCAAGGTACCCTGTGTCCCAACCCACCATCCTATGTGCCCAGGGCTCTGTCC
PanPar_1A1  (215) TCACTGCCATGCGGCCTAGATTTTCAAGGTACCCGCTGTCCCAGCCCACCATCCTATGTGCCCAGGGTTCTGTCC

                  601                                                                     675
MusMus_1A1  (601) TTCAACTCAGACCGCATGAACTTCCTACAGCGAGTGAAGAACGTGCTCCTGGCCGTGTCAGAGAACTTTATGTGC
RatNor_1A1  (601) TCGAACACAGATCGCATGAACTTCCTGCAGCGGGTGAAGAACATGATTATTGCTTTGACAGAGAACTTTCTATGC
GalGal_1A1  (595) GACCATACCGATCACATGAACTTCCTCCAGCGTGTGAAGAATGTCATCTTTGATACCTCAAATCTTTTTCTCTGT
HomSap_1A1  (595) TCTCATTCAGATCACATGACCTTCCTGCAGCGGGTGAAGAACATGCTCATTGCCTTTTCACAGAACTTTCTGTGC
PanTro_1A1  (595) TCTCATTCAGATCACATGACCTTCCTGCAGCGGGTGAAGAACATGCTCATTGCCTTTTCACAGAACTTTCTGTGC
MacMul_1A1  (595) GCTCATTCAGATCACATGACCTTCCTGCAGCGGGTGAAGAACATGCTCATTGCCTTTTCACAGAACTTTCTGTGC
MacFas_1A1  (595) GCTCATTCAGATCACATGACCTTCCTGCAGCGGGTGAAGAACATGCTCATTGCCTTTTCACAGAACTTTCTGTGC
PapAnu_1A1  (595) GCTCATTCAGATCACATGACCTTCCTGCAGCGGGTGAAGAACATGCTCATTGCCTTTTCACAGAACTTTCTGTGC
BosTau_1A1  (595) TTTAACTCAGATCACATGACCTTCCTGCAGCGGGTGAAGAACATGTTCATCACCTTGTCAGAGAGTTTGCTGTGC
CanFam_1A1  (595) CTTAACTCAGATCACATGACCTTCCTACAGCGGGTGAAGAACATGCTCATTTTCTTGTCCGAGAGCTTTTTGTGC
FelCat_1A1  (595) CTTAACTCAGATCACATGACTTTCCTACAGCGGGTGAAGAACATGCTCATTCTTGGGTCAGAGGGCTTCCTGTGC
MusNig_1A1  (481) CTTAACTCAGATCACATGACCTTTTTACCGCGGGTGAAGAACATGCTCATTTTCTTGTCAGAGAGCTTTCTGTGC
UrsMar_1A1  (456) CTTAACTCTGATCACATGACCTTCTTACAGCGGGTGAAGAACATGCTCATTTTCTTGTCAGAGAACTTTCTGTGC
UrsThi_1A1  (456) CTTAACTCTGATCACATGACCTTCTNACAGCGGGTGAAGAACATGCTCATTTTCTTGTCAGAGAACTTTCTGTGC
ProLot_1A1  (455) CTTAACTCAGATCACATGACCTTTTTACCGCGGGTGAAGAACATGCTCATTTTCTTGTCAGAGAGCTTTCTGTGC
CanRuf_1A1  (454) CTTAACTCAGATCACATGACCTTCCTACAGCGGGTGAAGAACATGCTCATTTTCTTGTCCGAGAGCTTTTTGTGC
MusPut_1A1  (454) CTTAACTCAGATCACATGACCTTTTTACCACGGGTGAAGAACATGCTCATTTTCTTGTCAGAGAGCTTTCTGTGC
ChrBra_1A1  (453) CTTAACTCAGATCACATGACCTTCCTACAGCGGGTGAAGAACATGCTCATTTTCTTGTCCGAGAGCTTTTTGTGC
VulVul_1A1  (453) CTTAACTCAGATCACATGACTTTCCTACAGCGGGTGAAGAACATGCTCATTTTCTTGTCCGAGAGCTTTTTGTGC
ArcBin_1A1  (452) CTTAACTCGGATCACATGACCTTCCCACAGCGGGTGAAGAACATGCTCATTCTGGCATTGGAGAGCTTTCTGTGT
PanTig_1A1  (452) CGTAACTCAGATCACATGACTTTCCTCCAGCGGGTGAAGAACATGCTCATTCTTGTGTCAGAGGGCTTCCTGTGC
HyaHya_1A1  (361) CTTAACTCAGATCACATGACCTTCCTACAGCGGGTAAAGAACATGCTCATTCTCGCATCAGAGGGCTTTCTGTGC
PhoVit_1A1  (358) CTTAACTCAGATCACATGACCTTCCTACCGCGGGTGAAGAACATGCTCATTTTCTTGTCAGAGAGCTTTCTGTGC
CarSer_1A1  (330) CTTAACTCAGATCACATGACTTTCCTACAGCGGGTGAAGAACATGCTCATTCTTGTGTCAGAGGGCTTCCTGTGC
```

```
AciJub_1A1  (329) CTTAACTCAGATCACATGACTTTCCTACAGCGGGTGAAGAACATGCTCATTCTTGTGTCAGAGGGCTTCCTGTGC
PumCo2_1A1  (329) CTTAACTCAGATCACATGACTTTCCTACAGCGGGTGAAGAACATGCTCATTCTTGTGTCAGAGGGCTTCCTGTGC
PumCon_1A1  (326) CTTAACTCAGATCACATGACTTTCCTACAGCGGGTGAAGAACATGCTCATTCTTGTGTCAGAGGGCTTCCTGTGC
ParBru_1A1  (326) CTTAACTCAGATCACATGACCTTCCTACAGCGGGTAAAGAACATGCTCATTCTCGCATCAGAGGGCTTTCTGTGC
CivCiv_1A1  (324) TTTAACTCAGATCACATGACCTTCCTACAGCGGGTGAAGAACATACTCATTCTCGCATCAGAGGGCTTTCTGTGC
CroCro_1A1  (322) CTTAACTCAGATCACATGACCTTCCTACAGCGGGTAAAGAACATGCTCATTCTCGCATCAGAGGGCTTTCTGTGC
HerJav_1A1  (318) CTTAACTCAGATCACATGACCTTCCTACAGCGGGTGAAAAACATGCTCATTCTGGCGTCAGAGGGCTTTCTCTGC
MirAng_1A1  (317) CTTAACTCAGATCACATGACCTTCCTACCGCGGGTGAAGAACATGCTCATTTTCTTGTCAGAGAGCTTTCTGTGC
PriBen_1A1  (317) CTTAACTCAGATCACATGACTTTCCTACAGCGGGTGAAGAACATGCTCATCCTTGTGTCAGAGGGCTTCCTGTGC
ProCri_1A1  (315) CTTAACTCAGATCACATGACCTTCCTACAGCGGGTAAAGAACATGCTCATTCTCGCATCAGAGGGCTTCCTGTGC
LeoGeo_1A1  (314) CTTAACTCAGATCACATGACTTTCCTACAGCGGGTGAAGAACATGCTCATTCTTGTGTCAGAGGGCTTCCTGTGC
PanUnc_1A1  (313) CTTAACTCAGATCACATGACTTTCCTCCAGCGGGTGAAGAACATGCTCATTCTTGTGTCAGAGGGCTTCCTGTGC
LynRuf_1A1  (312) CTTAACTCAGATCACATGACTTTCCTACAGCGGGTGAAGAACATGCTCATTCTTGTGTCAGAGGGCTTCCTGTGC
CarAur_1A1  (310) CTTAACTCAGATCACATGACTTTCCTACAGCGGGTGAAGAACATGCTCATTCTTGTGTCAGAGGGCTTCCTGTGC
ParTem_1A1  (307) CTTAACTCAAATCACATGACTTTCCTACAGCGGGTGAAGAACATGCTCATTCTTGTGTCAGAGGGCTTCCTGTGC
CalUrs_1A1  (305) CTTAACTCAGATCACATGACCTTCCTACCGCGGGTGAAGAACATGCTCATTTTCTTGTCAGAGAGCTTTCTGTGC
LeoTig_1A1  (303) CTTAACTCAGATCACATGACTTTCCTACAGCGGGTGAAGAACATGCTCATTCTTGTGTCAGAGGGCTTCCTGTGC
PanLeo_1A1  (303) CTTAACTCAGATCACATGACTTTCCTCCAGCGGGTGAAGAACATGCTCATTCTTGTGTCAGAGGGCTTCCTGTGC
LynCan_1A1  (302) CTTAACTCAGATCACATGACTTTCCTACAGCGGGTGAAGAACATGCTCATTCTTGTGTCAGAGAGCTTTCTGTGC
LeoWie_1A1  (302) CTTAACTCAGATCACATGACTTTCCTACAGCGGGTGAAGAACATGCTCATTCTTGTGTCAGAGGGCTTCCTGTGC
PanOnc_1A1  (296) CTTAACACAGATCACATGACTTTCCTCCAGCGGGTGAAGAACATGCTCATTCTTGTGTCAGAGGGCTTCCTGTGC
PhoHoo_1A1  (293) CTTAACTCAGATCACATGACCTTCCTACCGCGGGTGAAGAACATGCTCATTTTCTTGTCAGAGAGCTTTCTGTGC
ArcFor_1A1  (292) CTTAACTCAGATCACATGACCTTCCTACCGCGGGTGAAGAACATGCTCATTTTCTTGTCAGAGAGCTTTCTGTGC
PanPar_1A1  (290) CTTAACTCAGATCACATGACTTTCCTCCAGCGGGTGAAGAACATGCTCATTCTTGTGTCAGAGGGCTTCCTGTGC

                  676                                                                       750
MusMus_1A1  (676) AGAGTGGTTTATTCCCCCTATGGGTCACTTGCCACTGAAATCTTACAGAAAGAGGTGACTGTCCAGGATCTTCTG
RatNor_1A1  (676) AGAGTGGTTTACTCCCCCTATGGGTCACTTGCCACTGAAATCTTACAGAAAGAGGTGACTGTCAAGGACCTTCTG
GalGal_1A1  (670) GACTTTATTTTTAAACCATATGAAAAACTGGCTTCTGAGTTCCTTCAGCGAGATGTGACCGTGATAGATCTCTTA
HomSap_1A1  (670) GACGTGGTTTATTCCCCGTATGCAACCCTTGCCTCAGAATTCCTTCAGAGAGAGGTGACTGTCCAGGACCTATTG
PanTro_1A1  (670) GACGTGGTTTATTCCCCGTATGCAACCCTTGCCTCAGAATTCCTTCAGAGAGAGGTGACTGTCCAGGACCTATTG
MacMul_1A1  (670) GACGTGGTTTATTCCCCATATGCAACCCTTGCCTCGGAATTCCTGCAGAGAGAGGTGACTGTCCAGAACCTATTG
MacFas_1A1  (670) GACGTGGTTTATTCCCCATATGCAACCCTTGCCTCGGAATTCCTGCAGAGAGAGGTGACTGTCCAGAACCTATTG
PapAnu_1A1  (670) GACGTGGTTTATTCCCCATATGCAACCCTTGCCTCGGAATTCCTGCAGAGAGAGGTGACTGTCCAGAACCTATTG
BosTau_1A1  (670) GATATGGTTTATTCCCCATACGGGCTGCTTGCCTCGGAAATCCTTCAGACAGACATGACTGTTCGGGATCTCATG
CanFam_1A1  (670) AATGTGGTTTACTCCCCATATGAACCACTTGCCTCGGAAGTCCTTCAGAAAGATGTGACAGTCCAGGAACTTATG
FelCat_1A1  (670) AATGTGGTTTATTCCCCATATGCGTCACTTGCTTCGGAAGTCCTTCAGAAAGATGTGACTGTCCAGGACCTTATG
MusNig_1A1  (556) AATGTGGTTTATTCCCCGTATGGAGCACTTGCCTCAGAAGTCCTTCAGAAAGATGTGACTGTCCAGGACCTGATG
UrsMar_1A1  (531) AATGTGGTTTATTTGCCGTACGGACCACTTGCCTCAGAAATCCTTCAGAAGGATGTGACTGTCCAGGACCTTTTG
UrsThi_1A1  (531) AATGTGGTTTATTTGCCGTACGGACCACTTGCCTCAGAAATCCTTCAGAAGGATGTGACTGTCCAGGACCTTTTG
ProLot_1A1  (530) AATGTGGTTTATTCCCCGTATGGAGCACTTGCCTCAGAAGTCCTTCAGAAAGATGTGACTGTCCAGGACCTAATG
CanRuf_1A1  (529) AATGTGGTTTACTCCCCATATGAACCACTTGCCTCGGAAGTCCTTCAGAAAGATGTGACAGTCCAGGAACTTATG
MusPut_1A1  (529) AATGTGGTTTATTCCCCGTATGGAGCACTTGCCTCAGAAGTCCTTCAGAAAGATGTGACTGTCCAGGACCTGATG
ChrBra_1A1  (528) AATGTGGTTTACTCCCCATATGAACCACTTGCCTCGGAAGTCCTTCAGAAAGATGTGACAGTCCAGGAACTTATG
VulVul_1A1  (528) AATGTGGTTTATTCCCCATATGAGCCACTTGCCTCGGAAGTCCTTCAGAAAGATGTGACAGTCCAGGAACTTATG
ArcBin_1A1  (527) GACGTGGTTTATTCCCCATATGCGCCACTCGCCTCGGAAATCCTTCAGAGGGATGTGACCGTCCAGGACCTGATG
PanTig_1A1  (527) AATGTGGTTTATTCCCCATATGCATCACTTGCTTCGGAAGTCCTTCAGAAAGATGTGACTGTCCAGGACCTTATG
HyaHya_1A1  (436) AACATGGTTTACTCC------------------------------------------------------------
PhoVit_1A1  (433) AATGTGGTTTACTCC------------------------------------------------------------
CarSer_1A1  (405) AATGTGGTTTACTCCCCATATGCATCACTTGCTTCGGAAGTCCTGCAGAAAGATGTGACTGTCCAGGACCTTATG
AciJub_1A1  (404) AATGTGGTTTACTCCCCCTATG-----------------------------------------------------
PumCo2_1A1  (404) AATGTGGTTTACTCCCCATATGAACCCCT----------------------------------------------
PumCon_1A1  (401) AATGTGGTTTACTCCCCCTATA-----------------------------------------------------
ParBru_1A1  (401) AACATGGTTTACTCCCCCTATG-----------------------------------------------------
CivCiv_1A1  (399) AACGTGGTTTACTCCCCCTATG-----------------------------------------------------
CroCro_1A1  (397) AACATGGTTTACTCCCCCTATG-----------------------------------------------------
HerJav_1A1  (393) AACGTGGTTTACTCCCCCTATG-----------------------------------------------------
MirAng_1A1  (392) AATGTGGTTTACTCCCCCTATG-----------------------------------------------------
PriBen_1A1  (392) AATGTGGTTTACTCCCCCTATG-----------------------------------------------------
ProCri_1A1  (390) AACATGGTTTACTCCCCCTATG-----------------------------------------------------
LeoGeo_1A1  (389) AATGTGGTTTACTCCCCCTATG-----------------------------------------------------
PanUnc_1A1  (388) AATGTGGTTTACTCCCCCTATGCATCACTTGCTTCGGA-------------------------------------
LynRuf_1A1  (387) AATGTGGTTTACTCCCCCTAT------------------------------------------------------
CarAur_1A1  (385) AATGTGGTTTACTCCCCCTATG-----------------------------------------------------
ParTem_1A1  (382) AATGTGGTTTACTCCCCCTATG-----------------------------------------------------
CalUrs_1A1  (380) AATGTGGTTTACTCCCCCTATGCGCCCCTTGA-------------------------------------------
LeoTig_1A1  (378) AATGTGGTTTACTCCCCCTATGCACCCCTTGA-------------------------------------------
PanLeo_1A1  (378) AATGTGGTTTACTCCCCCTATGG----------------------------------------------------
LynCan_1A1  (377) AATGTGGTTTATTCCCCGTATGGAGCACTTGCCTCAGAAGTCCTTCAGAAAGATGTGACTGTCCAGGACCTAATG
LeoWie_1A1  (377) AATGTGGTTTACTCCCCCTATG-----------------------------------------------------
PanOnc_1A1  (371) AATGTGGTTTACTCCCCCTATG-----------------------------------------------------
```

```
PhoHoo_1A1  (368) AATGTGGTTTACTCCCCCTATG-----------------------------------------------------
ArcFor_1A1  (367) AATGTGGTTTACTCCCCCTATG-----------------------------------------------------
PanPar_1A1  (365) AATGTGGTTTACTCCCCCTATG-----------------------------------------------------

                  751                                                                     825
MusMus_1A1  (751) AGCCCTGCATCTATCTGGCTGATGAGAAGTGACTTTGTGAAAGATTACCCCAGGCCCATCATGCCCAACATGGTT
RatNor_1A1  (751) AGTCCTGCATCTATCTGGCTGATGAGAAACGACTTTGTGAAAGATTACCCCAGGCCCATCATGCCCAACATGGTT
GalGal_1A1  (745) CGTAAGGCTTCCGTATGGCTTCTGAGGTATGACTTTGTGTTAGATTATCCAAGGCCATTGATGCCCAACATAATT
HomSap_1A1  (745) AGCTCTGCATCTGTCTGGCTGTTTAGAAGTGACTTTGTGAAGGATTACCCTAGGCCCATCATGCCCAATATGGTT
PanTro_1A1  (745) AGCTCTGCATCTGTCTGGCTGTTTAGAAGTGACTTTGTGAAGGATTACCCTAGGCCCATCATGCCCAATATGGTT
MacMul_1A1  (745) AGCTCTGCATCTGTCTGGCTGCTTAGAAGTGACTTTGTGAAGGATTACCCTAGGCCCATCATGCCCAATATGGCT
MacFas_1A1  (745) AGCTCTGCATCTGTCTGGCTGCTTAGAAGTGACTTTGTGAAGGATTACCCTAGGCCCATCATGCCCAATATGGCT
PapAnu_1A1  (745) AGCTCTGCATCTGTCTGGCTGCTTAGAAGTGACTTTGTGAAGGATTACCCTAGGCCCATCATGCCCAATATGGCT
BosTau_1A1  (745) AGTTTTGGGTCTGTCTGGATTCTCAGAAGTGACTTTGTGTTTAATTTCCCAAGACCCATCATGCCCAACATAGTT
CanFam_1A1  (745) GGCTCTGCATCCATCTGGCTTCTCAAAGGTGACTTTGTCAAGGATTACTCCAGGCCCATCATGCCCAGCATGGTT
FelCat_1A1  (745) GGCTCCGCATCGGTCTGGCTTTTCAGAAGTGACTTTGTAAAGGATTACTCCAGGCCCATCATGCCCAACATGGTT
MusNig_1A1  (631) AGCTCTGCATCTGTCTGGATTCTCAGAAGTGACTTTGT-------------------------------------
UrsMar_1A1  (606) GGCTCCGGCTCTGTCTGGCTTCTCAGAAGTGACTTTGTCAAAGAT------------------------------
UrsThi_1A1  (606) GGCTCCGGCTCTGTCTGGCTTCTCAGAAGTGACTTTGTCAA----------------------------------
ProLot_1A1  (605) GGCTCTGCGTCTGTCTGGCTTCTCAGAACAGACTTTGTC------------------------------------
CanRuf_1A1  (604) GGCTCTGCATCCATCTGGCTTCTCAAAGGTGACTTTGTC------------------------------------
MusPut_1A1  (604) AGCTCTGCATCTGTCTGGATTCTCAGAAGTGACTTTGTAAAGGA-------------------------------
ChrBra_1A1  (603) GGCTCTGCATCCATCTGGCTTCTCAAAGGTGACTTTGTCAAAGA-------------------------------
VulVul_1A1  (603) GGCTCTGCATCCATCTGGCTTCTCAAAGGTGACTTTGTCAAA---------------------------------
ArcBin_1A1  (602) GCCTCCGGGTCAGTCTGGCTTTTCAGAAACGACTTTGTAAAG---------------------------------
PanTig_1A1  (602) GGCTCCGCATCGGTCTGGCTTTTCAGAAGTGACTTTGT-------------------------------------
HyaHya_1A1  (451) ---------------------------------------------------------------------------
PhoVit_1A1  (448) ---------------------------------------------------------------------------
CarSer_1A1  (480) GGCTCCGCATC----------------------------------------------------------------
AciJub_1A1  (426) ---------------------------------------------------------------------------
PumCo2_1A1  (433) ---------------------------------------------------------------------------
PumCon_1A1  (423) ---------------------------------------------------------------------------
ParBru_1A1  (423) ---------------------------------------------------------------------------
CivCiv_1A1  (421) ---------------------------------------------------------------------------
CroCro_1A1  (419) ---------------------------------------------------------------------------
HerJav_1A1  (415) ---------------------------------------------------------------------------
MirAng_1A1  (414) ---------------------------------------------------------------------------
PriBen_1A1  (414) ---------------------------------------------------------------------------
ProCri_1A1  (412) ---------------------------------------------------------------------------
LeoGeo_1A1  (411) ---------------------------------------------------------------------------
PanUnc_1A1  (426) ---------------------------------------------------------------------------
LynRuf_1A1  (408) ---------------------------------------------------------------------------
CarAur_1A1  (407) ---------------------------------------------------------------------------
ParTem_1A1  (404) ---------------------------------------------------------------------------
CalUrs_1A1  (412) ---------------------------------------------------------------------------
LeoTig_1A1  (410) ---------------------------------------------------------------------------
PanLeo_1A1  (401) ---------------------------------------------------------------------------
LynCan_1A1  (452) GGCTCTGCGTCTGTCTGGCTTCTCAAAAGAGACTTTGT-------------------------------------
LeoWie_1A1  (399) ---------------------------------------------------------------------------
PanOnc_1A1  (393) ---------------------------------------------------------------------------
PhoHoo_1A1  (390) ---------------------------------------------------------------------------
ArcFor_1A1  (389) ---------------------------------------------------------------------------
PanPar_1A1  (387) ---------------------------------------------------------------------------

                  826                                                   882
MusMus_1A1  (826) TTTATTGGTGGTATAAATTGCCTTCAGAAAAAGCCCCTATCCCAG------------
RatNor_1A1  (826) TTTATTGGTGGGATAAACTGCCTTCAGAAAAAAGCCCTATCCCAG------------
GalGal_1A1  (820) GTGGTTGGAGGAATAAACTGTGCTCACAAGCAGCTACCTCAGGTGGGTCGTGCTCAG
HomSap_1A1  (820) TTTGTTGGTGGAATCAACTGCCTTCACCAAAATCCACTATCCCAG------------
PanTro_1A1  (820) TTCGTTGGTGGAATCAACTGCCTTCACCAAAATCCACTATCCCAG------------
MacMul_1A1  (820) TTCATTGGTGGAATCAACTGCCTTCACCAAAGTCCACTATCCCAG------------
MacFas_1A1  (820) TTCATTGGTGGAATCAACTGCCTTCACCAAAGTCCACTATCCCAG------------
PapAnu_1A1  (820) TTCATTGGTGGAATCAACTGCCTTCACCAAAGTCCACTATCCCAG------------
BosTau_1A1  (820) TTTGTTGGTGGGATCAACTGCGCTAGCAAAAAGCCACTCTCTCAG------------
CanFam_1A1  (820) TTTGTTGGTGGGATCAACTGTGCCAGCAAAAACCCACTATCCAAG------------
FelCat_1A1  (820) TTTATCGGTGGGATCAACTGTGCCGGCAAAAACCCACTGTCCCAG------------
MusNig_1A1  (669) ---------------------------------------------------------
UrsMar_1A1  (651) ---------------------------------------------------------
UrsThi_1A1  (647) ---------------------------------------------------------
ProLot_1A1  (644) ---------------------------------------------------------
CanRuf_1A1  (643) ---------------------------------------------------------
```

```
MusPut_1A1  (648) ---------------------------------------------------------
ChrBra_1A1  (647) ---------------------------------------------------------
VulVul_1A1  (645) ---------------------------------------------------------
ArcBin_1A1  (644) ---------------------------------------------------------
PanTig_1A1  (640) ---------------------------------------------------------
HyaHya_1A1  (451) ---------------------------------------------------------
PhoVit_1A1  (448) ---------------------------------------------------------
CarSer_1A1  (491) ---------------------------------------------------------
AciJub_1A1  (426) ---------------------------------------------------------
PumCo2_1A1  (433) ---------------------------------------------------------
PumCon_1A1  (423) ---------------------------------------------------------
ParBru_1A1  (423) ---------------------------------------------------------
CivCiv_1A1  (421) ---------------------------------------------------------
CroCro_1A1  (419) ---------------------------------------------------------
HerJav_1A1  (415) ---------------------------------------------------------
MirAng_1A1  (414) ---------------------------------------------------------
PriBen_1A1  (414) ---------------------------------------------------------
ProCri_1A1  (412) ---------------------------------------------------------
LeoGeo_1A1  (411) ---------------------------------------------------------
PanUnc_1A1  (426) ---------------------------------------------------------
LynRuf_1A1  (408) ---------------------------------------------------------
CarAur_1A1  (407) ---------------------------------------------------------
ParTem_1A1  (404) ---------------------------------------------------------
CalUrs_1A1  (412) ---------------------------------------------------------
LeoTig_1A1  (410) ---------------------------------------------------------
PanLeo_1A1  (401) ---------------------------------------------------------
LynCan_1A1  (490) ---------------------------------------------------------
LeoWie_1A1  (399) ---------------------------------------------------------
PanOnc_1A1  (393) ---------------------------------------------------------
PhoHoo_1A1  (390) ---------------------------------------------------------
ArcFor_1A1  (389) ---------------------------------------------------------
PanPar_1A1  (387) ---------------------------------------------------------
```
