# Supplementary material for: Evolution of a Major Drug Metabolizing Enzyme Defect in the Domestic Cat and Other Felidae: Phylogenetic Timing and the Role of Hypercarnivory
Source: PLoS One. 2011 Mar 28;6(3):e18046. doi: 10.1371/journal.pone.0018046 (PMC3065456; doi:10.1371/journal.pone.0018046)
Supplement: Figure S2 — Clustal X alignment of UGT1A6 exon 1 sequences. Inactivating mutations (highlighted in red; M1 to M12) within the coding region were defined as either a nucleotide sequence insertion or deletion non-divisible by 3, or a nucleotide substitution resulting in a nonsense (premature stop) codon. Mutation sequence positions (in bp) are relative to the adenine (+1) of the human UGT1A6 start codon. See Table S1 for the full species and common names corresponding to the species abbreviation given on the left side of each sequence. (PDF) [file pone.0018046.s002.pdf]

```
                     1                                                                    70
HomSap_1A6     (1) ATGGCCTGCCTCCTTCGCTCATTTCAGAGAATTTCTGCAGGGGTTTTCTTCTT---AGCACTTTGGGGCA
PanTro_1A6     (1) ATGGCCTGCCTCCTTCGCGCATTTCAGAGAATTTCTGCAGGGGTTTTCTTCTT---AGCACTTTGGGGCA
PonAbe_1A6     (1) ATGGCCTGCCTCCTTCGTGCATTTCAGAGAATTTCTGCAGGGGTTTTCTTCTT---AGCACTTTGGGGCA
PapAnu_1A6     (1) ATGGCCTGCCTGCTTCGTGCATTTCAGAGAATTTCCGCAGGGGTTTTCTTCTT---AGCACTTTGGGGCA
MacFas_1A6     (1) ATGGCCTGCCTGCTTCGTGCATTTCAGAGAATTTCCGCAGGGGTTTTCTTCTT---AGCACTTTGGGGCA
AciJub_1A6     (1) ATGGCCTGCCTCCCCCATGGGTTTCAGAAAGTTTCCCCAGGGGTTTTCCTCTT---AACGCTTTGGAGTG
PanTig_1A6     (1) ATGGCCTG--------------------------------------------------------------
PanOnc_1A6     (1) ATGGCCTG--------------------------------------------------------------
PanUnc_1A6     (1) ATGGCCTG-------------------------------------------------------------- M6: bp9-108
PanLeo_1A6     (1) ATGGCCTG--------------------------------------------------------------
PanPar_1A6     (1) ATGGCCTG--------------------------------------------------------------
LeoGeo_1A6     (1) ATGGCCTGCCTCCCCCCTGCGTTGCAGAAAGTTTCCGCAGGGGTTTTCCTCTT---AGCGCTTTGGAGTG
LeoTig_1A6     (1) ATGGCCTGCCTCCCCCCTGCGTTGCAGAAAGTTTCCGCAGGGGTTTTCCTCTT---AGCGCTTTGGAGTG
LeoWie_1A6     (1) ATGGCCTGCCTCCCCCCTGCGTTGCAGAAAGTTTCCGCAGGGGTTTTCCTCTT---AGCGCTTTGGAGTG
LynCan_1A6     (1) ATGGCCTGCCTCCCCCATGCGTTTCAGAAAGTTTCCGCAGGGGTTTTCCTCTT---AGCGCTTTGGAGTG
LynRuf_1A6     (1) ATGGCCTGCCTCCCCCATGCGTTTCAGAAAGTTTCCGCAGGGGTTTTCCTCTT---AGCGCTTTGGAGTG
ParTem_1A6     (1) ATGGCCTGCCTCCCCCATGCGTTTCAGAAAGTTTCCGCAGGGGTTTTCCTCTT---AGCGCTTTGGAGTG
PumCon_1A6     (1) ATGGCCTGCCTCCCCCATGCGTTTCAGAAAGTTTCCGCAGGGGTTTTCCTCTT---AGCGCTTTGGAGTG
PumCo2_1A6     (1) ATGGCCTGCCTCCCCCATGCGTTTCAGAAAGTTTCCGCAGGGGTTTTCCTCTT---AGCGCTTTGGAGTG
FelCat_1A6     (1) ATGGCCTGCCTCCCCCATGCGTTTCAGAAAGTTTCCGCAGGGGTTTTCCTCTT---AGCGCTTTGGAGTG
PriBen_1A6     (1) ATGGCCTGCCTCCCCCATGCGTTTCAGAAAGTTTCCGCAGGGGTTTTCCTCTT---AGCGCTTTGGAGTG
CarAur_1A6     (1) ATGGCCTGCCTCCCCCATGCGTTTCAGAAAGTTTCCGCAGGGGTTTTCCTCTT---AGCGCTTTGGAGTG
CarSer_1A6     (1) ATGGCCTGCCTCCCCCATGCGTTTCAGAAAGTTTCCGCAGGGGTTTTCCTCTT---AGCGCTTTGGAGTG
CroCro_1A6     (1) ATGGCCTGCCTCCTCCGTGCATTTCAGAAAGTTTCTGCAGGGGTCTTCTTCTT---AGTGCTTTGGGGTG
ParBru_1A6     (1) ATGGCCTGCCTCCTCCGTGCATTTCAGAAAGTTTCTGCAGGGGTCTTCCTCTT---AGTGCTTTGGGGTG
ProCri_1A6     (1) ----------------------------------------------------------------------
HyaHya_1A6     (1) ATGGCCTGCCTCCTCCGTGCATTTCAGAAAGTTTCTGCAGGGGTCTTCCTCTT---AGTGCTTTGGGGTG
BosTau_1A6     (1) ATGGCTTGCCTTCTT---------TGGAGAGTTTCCGTGGCGGTTTTCTTCTT---AGCACTTTGGGGCT
OviAri_1A6     (1) ATGGCTTGCCTTGCC---TTCTTCGCACGAGTTTCTGTGGCAGTTTTCTTCTT---AGCACTTTGGGGCT
SusScr_1A6     (1) ATGGCCTGCCTTCTCCGGGCGTTTTGGGCAGTTTCTGCAGCGGTTCTCCTCTC---AGCACTTTGGGGCA
MusMus_1A6a    (1) ATGGCTTGCCTTCTTCCTGCTGCTCAGACACTTCCTGCAGGGTTTCTCTTCCT---AGTGCTTTGGGCCT
MusMus_1A6b    (1) ATGGCTTGCCTTCTTCCTGCTGCTCAGACACTTCCTGCAGGGTTTCTCTTCCT---AGTGCTTTGGGCCT
RatNor_1A6     (1) ATGGCTTGCCTTCTTCCTGCTGCTC---GACTTCCTGCAGGCTTTCTCTTCTT---AGTGCTCTGGGGCT
OryCun_1A6     (1) ATGGCCTGCCTGCTTTCTGCAGCTCAGAGAGCGTCTGCGGGGGTTCTCTTCGT---GGCACTCTGGGGCA
EquCab_1A6a    (1) ATGGCCTGCCTTCTCCGTGCATTTTGGAGAGTTTCTGCAGTGGTTTTCTTCTT---AGCATTGTGGGGCA
EquCab_1A6b    (1) ATGGCCTGCCTTCTCCGTGCATTTGGGAGAGTTTCAGCAGGGGTTTTCTTCTT---AGCATTGTGGGGCA
EquCab_1A6c    (1) ATGGCCTGCCTTCTCTGTGCATTTTGGAGAGTTTCTGCAGGGGTTTTCTTCTT---ATTGCTGTGGGGCA
MusNig_1A6     (1) ATGACCTGCCTCCTCCAGTTGTTTTGGAAAGTT------------TTCTTCTT---AGTGCTCTGGGGGG
MusPut_1A6     (1) ATGGCCTGCCTCCTCCAGTTGTTTCGGAAAGTT------------TTCTTCTT---AGTGCTCTGGGGGG
MirAng_1A6     (1) ATGGCCTGCCTCCTCCACATGTTTCAGAAAGTT------------CTCCTCTT---AGTGCTCTGGGCGG
PhoVit_1A6     (1) ATGGCCTGCCTCCTCCACGTGTTTCAGAAAGTT------------CTCTTCTT---AGTGCTCTGGGCGG
UrsMar_1A6     (1) ----------------------------------------------------------------------
UrsThi_1A6     (1) ----------------------------------------------------------------------
ProLot_1A6     (1) ----------------------------------------------------------------------
CanFam_1A6     (1) ATGGCTCGCCTCCTTCATTTGTTTCAAAAGGTG------------TTCTTCCT---AATGCTCTGGGGGG
CanRuf_1A6     (1) ----------------------------------------------------------------------
ChrBra_1A6     (1) ----------------------------------------------------------------------
VulVul_1A6     (1) ----------------------------------------------------------------------
AilFul_1A6     (1) ----------------------------------------------------------------------
ArcBin_1A6     (1) ATGGCCTGCCTCCTCCGCGCGTTTCAGAAAGTTTCTGCNGGAGTTTTCTTCTT---AGTGCTGTGGGGAG
CivCiv_1A6     (1) ----------------------------------------------------------------------
HerJav_1A6     (1) ATGGCCTGCCTCCGCCATGCGTTTCAGAAAGTTTCTGCGGGGGTTTCCTCTT---AGTGCTTTGGGGTG
GalGal_1A6     (1) ATGGCTCTTCTATTTCTGTGTTTTTACCCACGTACTGGGATATTTTTTCTTCTTCTGTCATCTTTAATCT

                     71                                                                  140
HomSap_1A6    (68) TGGTTGTAGGTGACAAGCTGCTGGTGGTCCCTCAGGACGGAAGCCACTGGCTTAGTATGAAGGATATAGT
PanTro_1A6    (68) TGGTTGTAGGTGACAAGCTGCTGGTGGTCCCTCAGGACGGAAGCCACTGGCTTAGTATGAAGGATATAGT
PonAbe_1A6    (68) TGGTTGTAGGTGACAAGCTGCTGGTGGTCCCTCAGGATGGAAGCCACTGGCTTAGTATGAAGGATATAGT
PapAnu_1A6    (68) TGGTTGTAGGTGACAAGCTGCTGGTGATCCCTCAGGATGGAAGCCACTGGCTTAGTATGAAGGATATAGT
MacFas_1A6    (68) TGGTTGCAGGTGACAAGCTGCTGGTGATCCCTCAGGATGGAAGCCACTGGCTTAGTATGAAGGATATAGT
AciJub_1A6    (68) GGGGGGNAGGNGACAAGCTGCTGGNGGTTCCNCAGGANGGAAGCCCCGGGCTCAGTCTGAAGGACGCAGT
PanTig_1A6     (9) -----------------------------------------AGCCACGGGCTCAGTCTGAAGGACACAGT
PanOnc_1A6     (9) -----------------------------------------AGCCACGGGCTCAGTCTGAAGGACACAGT
PanUnc_1A6     (9) -----------------------------------------AGCCACGGGCTCAGTCTGAAGGACACAGT M6: bp9-108
PanLeo_1A6     (9) -----------------------------------------AGCCACGGGCTCAGTCTGAAGGACACAGT
PanPar_1A6     (9) -----------------------------------------AGCCACGGGCTCAGTCTGAAGGACACAGT
LeoGeo_1A6    (68) AGGTGGTAGGTGACAAGCTGCTGGTGGTTCCTCCGGATGGAAGCCACGGGCTCAGTCTGAAGGACACAGT
LeoTig_1A6    (68) AGGTGGTAGGTGACAAGCTGCTGGTGGTTCCTCCGGATGGAAGCCACGGGCTCAGTCTGAAGGACACAGT
LeoWie_1A6    (68) AGGTGGTAGGTGACAAGCTGCTGGTGGTTCCTCCGGATGGAAGCCACGGGCTCAGTCTGAAGGACACAGC
LynCan_1A6    (68) AGGTGGTAGGTGACAAGCTGCTGGTGGTTCCTCAGGATGGAAGCCACGGGCTCAGTCTGAAGGATGCAGT
LynRuf_1A6    (68) AGGTCGTAGGTGACAAGCTGCTGGTGGTTCCTCAGGATGGAAGCCACGGGCTCAGTCTGAAGGACGCAGT
ParTem_1A6    (68) AGGTGGTAGGTGACAAGCTGCTGGTGGTTCCTCAGGATGGAAGCCACGGGCTCAGTCTGAAGGACGCAGT
PumCon_1A6    (68) AGGTGGTAGGTGACAAGCTGCTGGTGGTTCCTCAGGATGGAAGCCACGGGCTCAGTCTGAAGGACGCAGT
PumCo2_1A6    (68) AGGTGGTAGGTGACAAGCTGCTGGTGGTTCCTCAGGATGGAAGCCACGGGCTCAGTCTGAAGGACGCAGT
FelCat_1A6    (68) AGGTGGTAGGTGACAAGCTGCTGGTGGTTCCTCAGGATGGAAGCCACGGGCTCAGTCTGAAGGACGCAGT
```

```
  PriBen_1A6   (68) AGGTGGTAGGTGACAAGCTGCTGGTGGTTCCTCAGGATGGAAGCCACGGGCTCAGTCTGAAGGACGCAGT
  CarAur_1A6   (68) AGGTGGTAGGTGACAAGCTGCTGGTGGTTCCTCAGGATGGAAGCCACGGGCTCAGTCTGAAGGACGCAGT
  CarSer_1A6   (68) AGGTGGTAGGTGACAAGCTGCTGGTGGTTCCTCAGGATGGAAGCCACGGGCTCAGTCTGAAGGACGCAGT
  CroCro_1A6   (68) AGGTTGTAGCTGACAAGCTGCTGGTGGTCCCTCAGGATGGAAGCCACTGGCTCAGTATGAAGGAGATAGT
  ParBru_1A6   (68) AGGTTGTAGCTGACAAGCTGCTGGTGGTCCCTCAGGATGGAAGCCACTGGCTCAGTATGAAGGAGATAGT
  ProCri_1A6    (1) ----------------------------------------------------------------------
  HyaHya_1A6   (68) AGGTTGTAGCTGACAAGCTGCTGGTGGTCCCTCAGGATGGAAGCCACTGGCTCAGTATGAAGGAGATAGT
  BosTau_1A6   (59) TCACCCTGGGGGACAGGCTGCTGGTGGTCCCTCAGGATGGAAGCCACTGGCTCAGCATGAAGGACATCGT
  OviAri_1A6   (65) TCGCCCTGGGGGACAGGCTGCTGGTGGTCCCTCAGGATGGAAGCCACTGGCTCAGCATGAAGGACATCAC
  SusScr_1A6   (68) CGGTTGCAGGGGACAGGCTGCTGGTGGTCCCTCAGGATGGAAGCCACTGGCTCAGCATGAAGGACATCGT
 MusMus_1A6a   (68) CAGTTCTAGGTGACAAGCTGCTGGTGGTCCCCCAGGATGGAAGCCACTGGCTTAGCATGAAGGAGATAGT
 MusMus_1A6b   (68) CAGTTCTAGGTGACAAGCTGCTGGTGGTCCCCCAGGATGGAAGCCACTGGCTTAGCATGAAGGAGATAGT
  RatNor_1A6   (65) CAGTTCTAGGTGACAAGCTGCTGGTGGTCCCCCAGGATGGCAGCCACTGGCTTAGCATGAAGGAGATAGT
  OryCun_1A6   (68) CGGTGCTGGGTGACAGGCTGCTGGTGGTGCCCCAGGATGGAAGCCACTGGCTTAGCATGCAGGACATAGT
 EquCab_1A6a   (68) TGAGTGTAGGTGACAAGCTGCTGGTGGTCCCTCAGGATGGAAGCCACTGGCTCAGTATGAAGGACATAAT
 EquCab_1A6b   (68) TGACTGTAGGTGACAAGCTGCTGGTGGTCCCTCAGGACGGAAGCCACTGGCTCAGTATGAAGGACATCAT
 EquCab_1A6c   (68) TGGTTGTAGGTGACAAGCTGCTGGTAGTCCCTCAGGATGGAAGCCACTGGCTCAGTATGAAGGACATAAT
  MusNig_1A6   (56) AGGTTGTAGGTGACAGGCTGCTGGTGGTCCCTCAGGATGGAAGCCACTGGCTCAGTATGAAGGACATAGT
  MusPut_1A6   (56) AGGTTGTAGGTGACAGGCTGCTGGTGGTCCCTCAGGATGGAAGCCACTGGCTCAGTATGAAGGACATAGT
  MirAng_1A6   (56) GGGTTGTAGGTGACAAGCTGCTGGTGGTTCCTCAGGATGGAAGCCACTGGCTCAGTATGAAGGACATAGT
  PhoVit_1A6   (56) GGGTTGTAGGTGACAAGCTGCTGGTGGTCCCTCAGGATGGAAGCCACTGGCTCAGTATGAAGGACATAGT
  UrsMar_1A6    (1) ---------------------------------------------ACTGGCTCAGTATGAAGGACATAGT
  UrsThi_1A6    (1) ----------------------------------------------CTGGCTCAGTATGAAGGACATAGT
  ProLot_1A6    (1) ----------------------------------------------------------------------
  CanFam_1A6   (56) AGGCTGTAGGTGACAAGCTTCTGGTGGTCCCTCAGGACGGAAGCCACTGGCTCAGTATGGAGAACATAGT
  CanRuf_1A6    (1) ----------------------------------------------CTGGCTCAGTATGGAGAACATAGT
  ChrBra_1A6    (1) ----------------------------------------------CTGGCTCAGTATGGAGAACATAGT
  VulVul_1A6    (1) --------------------------------------------CACTGGCTCAGTATGGAGAACATAGT
  AilFul_1A6    (1) ----------------------------------------------------------------------
  ArcBin_1A6   (68) AGGTCGTATGTGACAAGCTGCTGGGGGTGCCTCAGGATGGAAGCCACTGGCTCAGTATGAAGGAGATAGT
  CivCiv_1A6    (1) ----------------------------------------------------------------------
  HerJav_1A6   (68) AGGTTGTAGGTGACAAGCTGCTGGTGGTGCCTCAGGACGGAAGCCACTGGCTCAGTATGAAGGAGATAGT
  GalGal_1A6   (71) TTGCTGAATGTGGAAAGATCCTGGTGATACCTCAGGATGGAAGTCATTGGCTCAGTATGCAACCAGTAGT

                    141                                                                210
  HomSap_1A6  (138) TGAGGTTCTCAGTGACCGGGGTCATGAGATTGTAGTGGTGGTGCCTGAAGTTAATTTGCTTTTGAAA---
  PanTro_1A6  (138) TGAGGTTCTCAGTGACCGGGGTCACGAGATTGTAGTGGTGGTGCCTGAAGTTAATTTGCTTTTGAAA---
  PonAbe_1A6  (138) TGAGGTTCTCAGTGACCGGGGGCATGACATTGTAGTGGTGGTGCCTGAAGTTAATTTGCTTTTGAAA---
  PapAnu_1A6  (138) TGAAGTTCTCAGTGACCGGGGGCATGACATTGTAGTGGTGGTGCCTGAAGTCAATTTGCTTTTGAAA---
  MacFas_1A6  (138) TGAAGTTCTCAGTGACCGGGGGCATGACATTGTAGTGGTGGTGCCTGAAGTCAATTTGCTTTTGAAA---
  AciJub_1A6  (138) TGAGCTTCTCAGCGAGAAGGGACATGACGTGGTAGTGCTGGTGTCAGAGGTCAATCTGCTTCTGAAG---
  PanTig_1A6   (38) TGAGCTTCTCAGTGAGAAGGGACATGACATGGTAGTGCTGGTGTCAGAGGTCAATCTGCTTCTGAAG---
  PanOnc_1A6   (38) TGAGCTTCTCAGTGAGAAGGGACATGACATGGTAGTGCTGGTGTCAGAGGTCAATCTGCTTCTGAAG---
  PanUnc_1A6   (38) TGAGCTTCTCAGTGAGAAGGGACATGACATGGTAGTGCTGGTGTCAGAGGTCAATCTGCTTCTGAAG---
  PanLeo_1A6   (38) TGAGCTTCTCAGTGAGAAGGGACATGACATGGTAGTGCTGGTGTCAGAGGTCAATCTGCTTCTGAAG---
  PanPar_1A6   (38) TGAGCTTCTCAGTGAGAAGGGACATGACATGGTAGTGCTGGTGTCAGAGGTCAATCTGCTTCTGAAG---
  LeoGeo_1A6  (138) TGAGCTTCTCAGTGAGAAGGGACATGACATGGTAGTGCTGGTGTCAGAGGTCAATCTGCTTCTGAAG---
  LeoTig_1A6  (138) TGAGCTTCTCAGTGAGAAGGGACATGACATGGTAGTGCTGGTGTCAGAGGTCAATCTGCTTCTGAAG---
  LeoWie_1A6  (138) TGAGCTTCTCAGTGAGAAGGGACATGACATGGTGGTGCTGGTGTCAGAGGTCAATCTGCTTCTGAAG---
  LynCan_1A6  (138) TGAGCTTCTCAGTGAGAAGGGACATGACATGGTAGTGCTGGTGTCAGAGGTCAATCTGCTTCTGAAG---
  LynRuf_1A6  (138) TGAGCTTCTCAGTGAGAAGGGACATGACATGGTAGTGCTGGTGTCAGAGGTCAATCTGCTTCTGAAG---
  ParTem_1A6  (138) TGAGCTTCTCAGTGAGAAGGGACATGACATGGTAGTGCTGGTGTCAGAGGTCAATCTGCTTCTGAAG---
  PumCon_1A6  (138) TGAGCTTCTCAGTGAGAAGGGACATGACGTGGTAGTGCTGGTGTCAGAGGTCAATCTGCTTCTGAAG---
  PumCo2_1A6  (138) TGAGCTTCTCAGTGAGAAGGGACATGATGTGGTAGTGCTGGTGTCAGAGGTCAATCTGCTTCTGAAG---
  FelCat_1A6  (138) TGAGCTTCTCAGTGAGAAGGGACATGACGTGGTAGTGCTGGTGTCAGAGGTCAATCTGCTTCTGAAG---
  PriBen_1A6  (138) TGAGCTTCTCAGTGAGAAGGGACATGACGTGGTAGTGCTGGTGTCAGAGGTCAATCTGCTTCTGAAG---
  CarAur_1A6  (138) TGAGCTTCTCAGTGAGAAGGGACATGACGTGGTAGTGCTGGTGTCAGAGGTCAATCTGCTTCTGAAG---
  CarSer_1A6  (138) TGAGCTTCTCAGTGAGAAGGGACATGACGTGGTAGTGCTGGTGTCAGAGGTCAATCTGCTTCTGAAG---
  CroCro_1A6  (138) TGAGTTCCTCAGTGAGAAGGGGCATGACATTGTAGTGCTGGTGCCAGAGGTCAATCTGCTTCTGAAA---
  ParBru_1A6  (138) TGAGTTCCTCAGTGAGAAGGGCCATGACATTGTAGTGCTGGTGCCAGAGGTCAATCTGCTTCTGAAA---
  ProCri_1A6    (1) ------------------------------------------------------TCTGCTTCTGAAA---
  HyaHya_1A6  (138) TGAGTTCCTCAGTGAGAAGGGCCATGACATTGTAGTGCTGGTGCCAGAGGTCAATCTGCTTCTGAAA---
  BosTau_1A6  (129) TGAGCATCTCAGTGAGAAAGGGCATGAGATCGTGGTGGTGGTGCCTGAAGTCAACCTGCTCCTCCAA---
  OviAri_1A6  (135) TGAACGTCTCAGCGAGAAAGGGCATGAGATCGTGGTGGTGGTGCCCAAAGTCAACCTGCTCCTCCAA---
  SusScr_1A6  (138) TGTGCGGCTCAGTGAGAAAGGGCACGAGATCGTGGTGGTGGCGCCGGAAATCAACCTGCTTCTCACA---
 MusMus_1A6a  (138) AGAACATCTCAGTGAACGAGGACATGACATTATGGTCCTAGTGCCAGAAGTCAATTTGCTTTTGGGA---
 MusMus_1A6b  (138) GGAGCATCTCAGTGAACGAGGACATGACATTGTGGTCCTAGTGCCAGAAGTCAATTTGCTTTTGGGA---
  RatNor_1A6  (135) GGAGCACCTCAGTGAACGCGGACACGACATTGTGGTGCTAGTGCCAGAAGTCAATTTGCTTTTGGGA---
  OryCun_1A6  (138) TGAGGCTCTTGGGGCGAGGGGGCATGAAATCGTGGTGCTGGTGCCAGAAGTCAACTTGCTCTTGAGA---
 EquCab_1A6a  (138) TGAGCCTCTCAGTGAGAAGGGGCATGACATCGTGGTGCTGGTGCCAGAAGTCAATTTGCTCCTGAAA---
 EquCab_1A6b  (138) TGAGCCTCTCAGTGAGAAGGGGCATGACATCGTGGTGCTGGTGCCAGAAGTCAATTTGCTTCTGAAA---
 EquCab_1A6c  (138) TGAGCCTCTCAGTGAGAAGGGGCATGACATCGTGGTGCTGGTGCCGGAAGTCAGTCTTCTTGTGAAA---
  MusNig_1A6  (126) TGAGCTCCTCAGTGAGAAGGGGCATGACATTGTGGTGCTGGTGCCAGAAGTCAATTTGCTTCTGAAG---
  MusPut_1A6  (126) TGAGCTCCTCAGTGAGAAGGGGCATGACATTGTGGTGCTGGTGCCAGAAGTCAATTTGCTTCTGAAG---
  MirAng_1A6  (126) TGAGCTCCTCAGTCAGAGGGGGCACAACATTGTGGTGCTGGTACCAGAAGTCAATTTGCTTCTGAAG---
  PhoVit_1A6  (126) TGAGCTCCTCAGTCAGAAGGGGCACGACATTGTGGTGCTGGTACCAGAAGTCAATTTGCTGCTGAAG---
  UrsMar_1A6   (26) TGAGCTCCTCAGTGAGAAGGGGCATGACATTGTGGTGCTGGTGCCAGAAGTCAATTTGCTTCTGAAG---
  UrsThi_1A6   (25) TGAGCTCCTCAGTGAGAAGGGGCATGACATTGTGGTGCTGGTGCCAGAAGTCAATTTGCTTCTGAAG---
```

```
 ProLot_1A6    (1) -------------------GGGCATGACTTTGTGGTGCTGGTGCCCCAAAGCAATCTGCTTCGAAAT---
 CanFam_1A6  (126) TGAGCTCCTCAGTGAGAAGGGGCATGACATTGTGGTGCTGGTGCCAGAAGTCAATTTGCTTCTGAAG---
 CanRuf_1A6   (25) TGAGCTCCTCAGTGAGAAGGGGCATGACATTGTGGTGCTGGTGCCAGAAGTCAATTTGCTTCTGAAG---
 ChrBra_1A6   (25) TGAGCTCCTCAGTGAGAAGGGGCATGACATTGTGGTGCTGGTGCCAGAAGTCAATTTGCTTCTGAAG---
 VulVul_1A6   (27) TGAGCTCCTCAGTGAGAAGGGGCATGACATTGTGGTGCTGGTGCCAGAAGTCAATTTGCTTCTGAAG---
 AilFul_1A6    (1) -----------------------------------TGCTGGTGTCAGAGGGCAATCTGCTTCTGAAG---
 ArcBin_1A6  (138) TGAGCACCTCAGTGAGAAGGGACATGATATTGTAGTGCTGGCGCCAGAGGTCAATCTGATTCTGAAG---
 CivCiv_1A6    (1) ---------------------------------------------------ATCTGCTTCTGAAG---
 HerJav_1A6  (138) TGAGCTCCTCAGTGAGAAGGGACATGACCTTGTAGTGCTGGTGCCAGAGGTCAATCTGCTTCTGAAG---
 GalGal_1A6  (141) AGGAAAACTCCAGCAAAATGGACATGATGTTGTTGTGGTTGTACCGTCATCAAGTTTGTTTATGAAGTCA

                   211                                                                 280
 HomSap_1A6  (205) ---GAATCCAAATACTACACAAGAAAAATCTATCCAGTGCCGTATGACCAAGAAGAGCTGAAGAACCGTT
 PanTro_1A6  (205) ---GAATCCAAATACTACACAAGAAAAATCTATCCAGTGCCGTATGACCAAGAAGAGCTGAAGAACCGTT
 PonAbe_1A6  (205) ---GAATCCAAATACTACACAAGAAAAATCTATCCAGTGCCGTATGACCAAGAAGAGCTGAAGAACCGTT
 PapAnu_1A6  (205) ---GAATCCAAATACTACACAAGAAAAATCTATCCAGTGCCGTATGACCAAGAAGAGATGAAGAACCGTT
 MacFas_1A6  (205) ---GAATCCAAATACTACACAAGAAAAATCTATCCAGTGCCGTATGACCAAGAAGAGATGAAGAACCGTT
 AciJub_1A6  (205) ---GAATCCAAACACTACACGAGAAAAATCTATCTAGTGCCATTGGACCGGGAAGAGCCGGAGCACCGTT
 PanTig_1A6  (105) ---GAATCCAAACACTACACGAGAAAAATCTATCCAGTGCCATTTGACCGGGAAGAGCCGGAGCACCGTT
 PanOnc_1A6  (105) ---GAATCCAAACACTACACGAGAAAAATCTATCCAGTGCCATTTGACCGGGAAGAGCCGGAGCACCGTT
 PanUnc_1A6  (105) ---GAATCCAAACACTACACGAGAAAAATCTATCCAGTGCCATTTGACCGGGAAGAGCCGGAGCACCGTT
 PanLeo_1A6  (105) ---GAATCCAAACACTACACGAGAAAAATCTATCCAGTGCCATTTGACCGGGAAGAGCCGGAGCACCGTT
 PanPar_1A6  (105) ---GAATCCAAACACTACACGAGAAAAATCTATCCAGTGCCATTTGACCGGGAAGAGCCGGAGCACCGTT
 LeoGeo_1A6  (205) ---GAATCCAAACACTACACGAGAAAAATCTATCCAGTGCCATTTGACCGGGAAGAGCCAGAGCACCGTT
 LeoTig_1A6  (205) ---GAATCCAAACACTACACGAGAAAAATCTATCCAGTGCCATTTGACCGGGAAGAGCCAGAGCACCGTT
 LeoWie_1A6  (205) ---GAATCCAAACACTACGCGAGAAAAATCTATCCAGTGCCATTTGACCGGGAAGAGCCAGAGCACCGTT
 LynCan_1A6  (205) ---GAATCCAAACACTACACGAGAAAAATCTATCCAGTGCCATTTGACCGGGAAGAGCCGGAGCACCGTT
 LynRuf_1A6  (205) ---GAATCCAAACACCACACGAGAAAAATCTATCCAGTGCCATTTGACCGGGAAGAGCCAGAGCACCGTT
 ParTem_1A6  (205) ---GAATCCAAACACTACACGAGAAAAATCTATCCAGTGCCATTTGACCGGGAAGAGCCGGAGCACCGTT
 PumCon_1A6  (205) ---GAATCCAAACACTACACGAGAAAAATCTATCCAGTGCCATTTGACCGGGAAGAGCCGGAGCACCGTT
 PumCo2_1A6  (205) ---GAATCCAAACACTACACGAGAAAAATCTATCCAGTGCCATTTGACCGGGAAGAGCCGGAGCACCGTT
 FelCat_1A6  (205) ---GAATCCAAACACTACACGAGAAAAATCTATCCAGTGCCATTTGACCGGGAAGAGCCGGAGCACCGTT
 PriBen_1A6  (205) ---GAATCCAAACACTACACGAGAAAAATCTATCCAGTGCCATTTGACCGGGAAGAGCCGGAGCACCGTT
 CarAur_1A6  (205) ---GAATCCAAACACTACACGAGAAAAATCTATCCAGTGCCATTTGACCGGGAAGAGCCGGAGCACCGTT
 CarSer_1A6  (205) ---GAATCCAAACACTACACGAGAAAAATCTATCCAGTGCCATTTGACCGGGAAGAGCCGGAGCACCGTT
 CroCro_1A6  (205) ---GAATCCAAACACTACATGAGAAAAATCTACCCAGTGCCATTTGACCAGGAAGAGCTGCAGAACCGTT
 ParBru_1A6  (205) ---GAATCCAAACACTACGTGAGAAAAATCTACCCAGTGCCTTTTGACCACGAAGAGCTGCAGAACCGTT
 ProCri_1A6   (14) ---GAATCCAAACACTACATGAGAAAAATCCACCCAGTGCCATTTGACCAGCAAGAGCTGAAGAACCGTT
 HyaHya_1A6  (205) ---GAATCCAAACACTACGTGAGAAAAATCTACCCAGTGCCTTTTGACCACGAAGAGCTGCAGAACCGTT
 BosTau_1A6  (196) ---GAGTCCAAGCACTACACAAGGAAAATCCACCCAGTGCCGTTCAACCAGGAGGAGCTGGAAGCCCGTT
 OviAri_1A6  (202) ---GAGTCCAAGCACTACACAAGGAGAATCCACCCAGTGCCCTACGACCAGGAGGAGCTGGAAGCCCGTT
 SusScr_1A6  (205) ---GAATCCAAATACTACACCAGGAAGATCTATCCGGTGCCCTACGACCAAGAGGAGCTGGAGGGCCGTT
MusMus_1A6a  (205) ---GAATCCAAATACTACAGGAGGAAAATCTTCTCAGTTACCTACAGCCTAGAAGAACTGCAGACCCGTT
MusMus_1A6b  (205) ---GAATCCAAATACTACAGGAGGAAAATTTTCTCAGTTCCCTACAGCCTAGAAGAACTACAGACCCGTT
 RatNor_1A6  (202) ---GAATCCAAATACTACAGGAGGAAAAGCTTCCCGGTCCCCTACAACCTAGAAGAGTTGCGGACCCGCT
 OryCun_1A6  (205) ---GAATCCAGGTTCTACACGAGGAGAATCTACCCAGTGCCGTTTGACCAGGAGGAGCAGAGTTACCGGT
EquCab_1A6a  (205) ---GAATCGAAATACTACACAAGAAGAATCTATCCAGTGCCCTACGACGAGGAAGAGATGGTGAGCCGTT
EquCab_1A6b  (205) ---GAATCTGAATACTACACAAGAAGAATCTATCCAGTGCCCTATGAAGAGGAAGAGCTGATGAGCCGTT
EquCab_1A6c  (205) ---GAATCCAAATACTACACAAGAAGAATCTATCCAGTGCCCTACGACGAGGAAGAAATGGTGAGCCGTT
 MusNig_1A6  (193) ---GAGTCCAAGCACTACACAAGAAAAACCTATCCAGTGCCGTTCCAACAGGAGGAGCTGGAGAATCGGT
 MusPut_1A6  (193) ---GAGTCCAAGCACTACACAAGAAAAACCTATCCAGTGCCGTTCCAACAGGAGGAGCTGGAGAATCGGT
 MirAng_1A6  (193) ---GAATCCAAGCACTACACAAGAAGAAGCTACCCGGTGCCATTCGATCAGAAAGAGCTGGGGAATCGTT
 PhoVit_1A6  (193) ---GAATCCAAGCACTACACAAGAAAAACCTACCCAGTGCCATTCGATCAGAAAGAGCTGGAGAATCGTT
 UrsMar_1A6   (93) ---GAATCCAAGCACTATGCGAGAAAAATCTACCCGGTGCCGTATGACCAGGAAGAGCTGCAGGCTCGTT
 UrsThi_1A6   (92) ---GAATCCAAGCACTACGCGAGAAAAATCTACCCGGTGCCGTATGACCAGGAAGAGCTGCAGGCTCGTT
 ProLot_1A6   (49) ---GAATCCTCGCACTACACGAGAAGGATCTATCCAGTGCCCTTCGAGCGGGAGGAGCTGGGGGATCGTT
 CanFam_1A6  (193) ---GAATCCAAACACTACACGAGACAAATCTACTCAGTGCCATTTGGCCAGGAAGAGTTGGAGAACCGCT
 CanRuf_1A6   (92) ---GAATCCAAACACTACACGAGACAAATCTACTCAGTGCCATTTGGCCAGGAAGAGTTGGAGAACCGCT
 ChrBra_1A6   (92) ---GAATCCAAACACTACACGAGACAAATCTACTCAGTGCCATTTGGCCAGGAAGAGTTGGAGAACCGTT
 VulVul_1A6   (94) ---GAATCCAAACACTACACGAGACAAATCTACTCAGTGCCATTTGGCCAGGAAGAGTTGGAGAACCGTT
 AilFul_1A6   (33) ---GAGTCCAAGCACTACACAAGGAAAATCTACCCAGTGCCATTCGACCAGGAAGAGCTCGAGAATCGGT
 ArcBin_1A6  (205) ---GAATCCAAACACTACACGAGAAAAATCTATCCAGTGCCATTTGACCAGGAAGAGCTGGAGAAGCGTT
 CivCiv_1A6   (15) ---GAATCCAAACACTACACGAGAAAAATCTATCCAGTGCCATTTGACCAGGAAGAGCTGGAGAACCGTT
 HerJav_1A6  (205) ---GAATCCAAACACTACAGGAGAAAAACCTACCCAGTGCCATTTGACCAGGAGGAGCTGCAGAACCGTT
 GalGal_1A6  (211) AAGGAGCATCAGAATTATACAGTGAAAGTGTATCGAATACCTTACGAAGAAGAACATTTACATCTTGTGC

                   281                                                                 350
 HomSap_1A6  (272) ACCAATCATTTGGAAACAATCACTTTGCTGAGCGATCATTCCTAACTGCTCCTCAGACAGAGTACAGGAA
 PanTro_1A6  (272) ACCAATCATTTGGAAACAATCACTTTGCTGAGCGATCATCCCTGACTGCTCCTCAGACAGAGTACAGGAA
 PonAbe_1A6  (272) ACCAATCATTTGGAAACAATCACTTTGCTGAGCGATCATTCCTGACTGCTCCTCAGACAGAGTACAGGAA
 PapAnu_1A6  (272) ACCAATTGTTTGGAAACAATCACTTTGCTGAGCGATCATTCCTGACTGCTCCTCAGACAGAGTACAGGAA
 MacFas_1A6  (272) ACCAATTGTTTGGAAACAATCACTTTGCTGAGCGATCATTCCTGACTGCTCCTCAGACAGAGTACAGGAA
 AciJub_1A6  (272) TCTGATCTTTTGGAAACAGTCACTTCGCTGAGAGGTGGCTCCTGAACCCTGCTGTGAGGGAGGACAGGAA
 PanTig_1A6  (172) TCTGATCTTTTGGAAACAGTCACTTCGCTGAGAGGTGGCTCCTGAACCCTGCTGTGCGGGAGGACAGGAA
 PanOnc_1A6  (172) TCTGATCTTTTGGAAACAGTCACTTCGCTGAGAGGTGGCTCCTGAACCCTGCTGTGCGGGAGGACAGGAA
 PanUnc_1A6  (172) TCTGATCTTTTGGAAACAGTCACTTCGCTGAGAGGTGGCTCCTGAACCCTGCTGTGCGGGAGGACAGGAA M1: bp274-276
 PanLeo_1A6  (172) TCTGATCTTTTGGAAACAGTCACTTCGCTGAGAGGTGGCTCCTGAACCCTGCTGTGCGGGAGGACAGGAA
 PanPar_1A6  (172) TCTGATCTTTTGGAAACAGTCACTTCGCTGAGAGGTGGCTCCTGAACCCTGCTGTGCGGGAGGACAGGAA
```

```
LeoGeo_1A6   (272) TCTGATCTTTTGGAAATAGTCACTTCGCTGAGAGGTGGCTCCTGAACCCTGCTGTGAGGGAGGACAGGAA
LeoTig_1A6   (272) TCTGATCTTTTGGAAATAGTCACTTCGCTGAGAGGTGGCTCCTGAACCCTGCTGTGAGGGAGGACAGGAA
LeoWie_1A6   (272) TCTGATCTTTTGGAAATAGTCACTTCGCTGAGAGGTGGCTCCTGAACCCTGCTGTGAGGGAGGACAGGAA
LynCan_1A6   (272) TCTGATCTTTTGGAAACAGTCACTTCGCTGAGAGGTGGCTCCTGAACCCTGCTGTGAGGGAGGACAGGAA
LynRuf_1A6   (272) TCTGATCTTCTGGAAACAGTCACTTCGCTGAGAGGTGGCTCCTGAACCCTGCTGTGAGGGAGGACAGGAA
ParTem_1A6   (272) TCTGATCTTTTGGAAACAGTCACTTCGCTGAGAGGTGGCTCCTGAACCCTGCTGCGAGGGAGGACAGGAA
PumCon_1A6   (272) TCTGATCTTTTGGAAACAGTCACTTCACTGAGAGGTGGCTCCTGAACCCTGCTGTGAGGGAGGACAGGAA
PumCo2_1A6   (272) TCTGATCTTTTGGAAACAGTCACTTCACTGAGAGGTGGCTCCTGAACCCTGCTGTGAGGGAGGACAGGAA
FelCat_1A6   (272) TCTGATCTTTTGGAAACAGTCACTTCGCTGAGAGGTGGCTCCTGAACCCGGCTGTGAGGGAGGACAGGAA
PriBen_1A6   (272) TCTGATCTTTTGGAAACAGTCACTTCGCTGAGAGGTGGCTCCTGAACCCGGCTGTGAGGGAGGACAGGAA
CarAur_1A6   (272) TCTGATCTTTTGGAGATTGTCACTTCGCTGAGAGGTGGCTCCTGAACCCTGCTGTGAGGGAGGACAGGAA
CarSer_1A6   (272) TCTGATCTTTTGGAGATTGTCACTTTGCTGAGAGGTGGCTCCTGAACCCTGCTGTGAGGGAGGACAGGAA
CroCro_1A6   (272) TCCAATCTTTTGGAAACAATCACTTTGTGGAGAAAGGGCTCCTGAGCGCTGCTCTGATGGAGTACAGGAA
ParBru_1A6   (272) TCTGATCTTTTGGAAGCAATCACTTTGTGGAGAAAGGGCTCCTGAGCGCTGCTCTGATGGAGTACAGGAA M10: bp274-276
ProCri_1A6    (81) TCCGATCTTTTGGAAACAATCACTTTGTGGAGAAAGGGCTCCTGAGCGCTGCTTTGATAGAGTACAGGAA
HyaHya_1A6   (272) TCCGGTCTTTTGGAAGCAATCACTTTGTGGAGAAAGGGCTCCTGAGCGCTGCTCTGATGGAGTACAGGAA
BosTau_1A6   (263) ACCGCTCCTTCGGGAAACACCACTTTTCTCCACGCTGGTTGGTGACTGCCCCTGTGGTGGAGTATAGGAA
OviAri_1A6   (269) ACCGTTCCTTCGGGAAACACCACTTTTCTCCACGCTGGCTGGTGACTGCCCCTATGGTGGAGTATAGGAA
SusScr_1A6   (272) ACCGGTCTTTTGGAAGAAACCACTTTTCGGAGCGATGGTTGCTGAACGCCGCTCAGGTGGAGTACAGGAA
MusMus_1A6a  (272) TTCGCACCTTTGGAAACAACCACTTTCTTCCCGGTGCCTCCCTGATGGGTCCTCTAAGAGAGTACAGGAA
MusMus_1A6b  (272) TTCGCACCTTTGGACGCAACCAGTTTGTTCCTGGTGCCCCCCTGATGGGTCCTCTAAGAGAGTACAGGAA
RatNor_1A6   (269) ATCGCTCCTTTGGGAACAACCACTTTGCTGCCAGTTCCCCCCTGATGGCTCCTCTAAGAGAGTACAGGAA
OryCun_1A6   (272) ATCGCACCTTTGGAGAAAAGCACTTTACTGACAGGTCCTGGCTGAGCGGGCCTCAGACGGAATACAGGAA
EquCab_1A6a  (272) ACCGCTCTTTTGGAAAGAATCACTTTGCTGAGAGATGGCTCCTAAATGCTGCTCAGACTGAGTATAGGAA
EquCab_1A6b  (272) TCCGCTCTTTTGGAAAGAATCAATTCGCTGAGAGATGGCTCCTGGATGCTGCTCAGACTGAGTACAGGAA
EquCab_1A6c  (272) TCTGTTCATTTGGCGACAATCACTTTGTTAAGAGATGGCTCCTGGATGCTGTTCAGACTGAGTACAGGAA
MusNig_1A6   (260) ACCGCTCTTTTGGAAACAATCACTTTGCTGAGAGATGGCTCCTGAACGCTGCTCAGATGGAATACAGGAA
MusPut_1A6   (260) ACCGCTCTTTTGGAAACAATCACTTTGCTGAGAGATGGCTCCTGAACGCTGCTCAGATGGAATACAGGAA
MirAng_1A6   (260) ACCACTTTTTTGGAAACGATCACTTTGCTGAGACATGGCTCCCGAACGCTGCTCAGATGGAGTACAGGAA
PhoVit_1A6   (260) ACCACTCTTTTGGAAACAATCACTTTGCTGAGAGATGGCTCCTGAACGCTGCTCAGATGGAGTACAGGAA
UrsMar_1A6   (160) ACCGCTCTTTTGGAAACAATCACTTTGCTGAGAGATGGCTCCTGAACGCGGCTCAAACGGAGTACAGGAA
UrsThi_1A6   (159) ACCGCTCTTTTGGAAACAATCACTTTGCTGAGAGATGGCTCCTGAACGCGGCTCAAACGGAGTACAGGAA
ProLot_1A6   (116) TCCGCTCTTTTGGAAACAATCACTTTGCTGAGAGATGCTTCCTGAACGCTGCTCAGGGGGAGGACAGGAA
CanFam_1A6   (260) ACCGCTCTTTTGGAAAGAATCACTTTGCTGAGAGATGGCTCCTGAACGCTGCTCAGATGGAGTACAGAAA
CanRuf_1A6   (159) ACCGCTCTTTTGGAAAGAATCACTTTGCTGAGAGATGGCTCCTGAACGCTGCTCAGATGGAGTACAGAAA
ChrBra_1A6   (159) ACCGCTCTTTTGGAAAGAATCACTTTGCTGAGAGATGGCTCCTGAACACTGCTCAGATGGAGTACAGAAA
VulVul_1A6   (161) ACCGCTCTTTTGGAAAGAACCACTTTGCTGAGAGATGGCTCCTGAACGCTGCTCAGATGGAGTACAGAAA
AilFul_1A6   (100) ACCGCTCTTTTGGAAACGGTCACTTTGCTGAGAGGTGGCTCCTGAATGCTGCTCAGACGGAGTACAGGAA
ArcBin_1A6   (272) TCCGAGCTTTTGGAAACAATCACTTTGCTGAGAGATGGCTCCTGAACGCTGCTCTGATGGAGTACAGGAA
CivCiv_1A6    (82) TCCGAGCTTTTGGAAACAATCACTTTGCTGAGAGATGGCTCCTGAACGCTGCTCTGATGGAGTACAGGAA
HerJav_1A6   (272) TCCGATCTTTTGGAAATAATCACTTTGCGGAGAGAGGGCTCCTGAGCGCCGCTCCGATGGAATACAGGAA
GalGal_1A6   (281) TCAAGTCATTTGTTAATGATCATTTTACTGAACAATCTTTATTGAATATTATTATTACAATGTATCAAAA

                   351                                                                420
HomSap_1A6   (342) TAACATGATTGTTATTGGCCTGTACTTCATCAACTGCCAGAGCCTCCTGCAGGACAG-GGACACCCTGAA
PanTro_1A6   (342) TAACATGATCGTTATTGGCCTGTACTTCATCAACTGCCAGAGCCTCCTGCAGGACAG-GGACACCCTGAA
PonAbe_1A6   (342) TAACATGATCGTTATTAGCCTGTACTTCATCAACTGCCAGAGCCTCCTGCAGGACAG-GGACACCCTGAA
PapAnu_1A6   (342) TAACATGGTCGTTATTGGCATGTACTTCATCAACTGCCAGAGCCTCCTGCAGGACGT-GGACACCCTGAA
MacFas_1A6   (342) TAACATGGTCGTTATTGGCATGTACTTCATCAACTGCCAGAGCCTCCTGCAGGACGT-GGGCACCCTGAA
AciJub_1A6   (342) TACCATGATTGTTACCGACACGTACTTCGCCAACTGCTAGAGCCTCCTGGAGGACTC--GCCACCTTGAG
PanTig_1A6   (242) TACCATGATTGTTACCGACACGTACTTCGCCAACCGCTAGAGCCTCCTGGAGGACTC--GCCACCTTGAG
PanOnc_1A6   (242) TACCATGATTGTTACCGACACGTACTTCGCCAACCGCTAGAGCCTCCTGGAGGACTC--GCCACCTTGAG
PanUnc_1A6   (242) TACCATGATTGTTACCGACACGTACTTCGCCAACCGCTAGAGCCTCCTGGAGGACTC--GCCACCTTGAG M2: bp379-381
PanLeo_1A6   (242) TACCATGATTGTTACCGACACGTACTTCGCCAACCGCTAGAGCCTCCTGGAGGACTC--GCCACCTTGAG
PanPar_1A6   (242) TACCATGATTGTTACCGACACGTACTTCGCCAACCGCTAGAGCCTCCTGGAGGACTC--GCCACCTTGAG M3: bp399
LeoGeo_1A6   (342) TACCGTGATTGTTACCGACACATACTTCGCCAACTGCTAGAGCCTCCTGGAGGACTC--GCCACCTTGAG
LeoTig_1A6   (342) TACCGTGATTGTTACCGACACATACTTCGCCAACTGCTAGAGCCTCCTGGAGGACTC--GCCACCTTGAG
LeoWie_1A6   (342) TACCGTGATTGTTACCGACACATACTTCGCCAACTGCTAGAGCCTCCTGGAGGACTC--GCCACCTTGAG
LynCan_1A6   (342) TACCATGATTGTTACCGACACATACTTCGCCAACTGCTAGAGCCTCCTGGAGGACTC--GCCACCTTGAG
LynRuf_1A6   (342) TACCATGATTGTTACCGACACATACTTCGCCAACTGCTAGAGCCTCCTGGAGGACTC--GCCACCTTGAG
ParTem_1A6   (342) TACCATGATTGTTACCGACACGTACTTCGCCAACTGCTAGAGCCTCCTGGAGGACTC--GCCACCTTGAG
PumCon_1A6   (342) TACCATGATTGTTACCGAC----ACTTCGCCAACTGCTAGAGCCTCCTGGAGGACTC--GCCACCTTGAG M7: bp361-364
PumCo2_1A6   (342) TACCATGATTGTTACCGAC----ACTTCGCCAACTGCTAGAGCCTCCTGGAGGACTC--GCCACCTTGAG
FelCat_1A6   (342) TACCATGATTGTTACCGACACGTACTTCGCCAACTGCTAGAGCCCCCTGGAGGACTC--GCCACCTTGAG
PriBen_1A6   (342) TACCATGATTGTTACCGACACGTACTTCGCCAACTGCTAGAGCCCCCTGGAGGACTC--GCCACCTTGAG
CarAur_1A6   (342) TACCATGATTGTTACCGACACGTACTTCGCCAACTGCTAGAGCCTCCTGGAGGACTC--GCCACCTTGAG
CarSer_1A6   (342) TACCATGATCGTTACCNACACGTACTTCGCCGACCGCTAGAGCCTCCTGGAGGACTC--GCCACCTTGAG
CroCro_1A6   (342) TAGCATGATTATTCTTGACATGTGGTTCACCAACTGCCAGAGCCTCCTGGGGGACTC-CGCCACCCTGAG
ParBru_1A6   (342) TAGCATGATTATCCTTGACATGTGGTTCACCAACTGCCAGAGCCTCCTGGGGGACTC-CGCCACCCTGAG
ProCri_1A6   (151) TAGCATGATTATTCTTGACATGTGGTTCACCAACTGCCAGAGCCTCCTGGGGGACTC-CACAACCCTGAG
HyaHya_1A6   (342) TAGCATGATTATCCTTGACATGTGGTTCACCAACTGCCAGAGCCTCCTGGGGGACTC-CGCCACCCTGAG
BosTau_1A6   (333) CAACATGATTGTCATCAATATGTACTTTCTCAACTGCCAGAGTCTCCTGAGGCACTC-AGACACCCTGCG
OviAri_1A6   (339) CAACATGATCGTCATCAACATGTACTTTCTCAACTGCCAGAGTCTCCTGAGGCACTC-GGGAACCCTGCG
SusScr_1A6   (342) TAACATGATTGTGATCAACATGTATTTCCTCACCTGCCAGAGCCTCCTGAGGGACGC-CGCCACCCTGAG
MusMus_1A6a  (342) CAACATGATTGTCGTGGACATGTTCTTTTCCAACTGCCAGAGCCTCCTGAAGGACTC-TGCCACCCTCAG
MusMus_1A6b  (342) CAGCATGCTTACATTGGAGATGTTCTTTTCCAACTGCCAGAGCCTCCTGAAGGACTC-TGCCACCCTCAG
RatNor_1A6   (339) CAACATGATTGTCATTGACATGTGCTTTTTCAGCTGCCAGAGCCTCCTGAAGGACTC-GGCCACCCTCAG
OryCun_1A6   (342) TAACATGGTTGTGATTGACATGTACTTCATCAACTGCCAGAGCCTGCTGAGACACGG-CGACACCTTGGA
```

```
EquCab_1A6a  (342) TAACATGATCGTTATCAACATGTACTTCACCAACTGCCAGAGCCTCCTGAACCACTC-TGAGACCCTGAG
EquCab_1A6b  (342) TACCATGATCGTTATCCACATGTACTTCACCAACTGCCAGAGCCTCCTGAACCACTC-TGAGACCCTGAG
EquCab_1A6c  (342) TACCATGGTGGTTATGGAGCTGTGCTTCTTCAACTGCCAGAGCCTCCTGAACCACTC-TGAGACCCTGAG
 MusNig_1A6  (330) TAGCATGATTGTTATTGACATGTATTTCACCACCTGCCAGAGCCTCCTGGAGGACAC-AGCCACCCTGTG
 MusPut_1A6  (330) TAGCATGATTGTTATTGACATGTATTTCACCACCTGCCAGAGCCTCCTGGAGGACAC-AGCCACCCTGAG
 MirAng_1A6  (330) TAGCATGATGGTTATTGACATGTACTTCACCAACTGCCAGAGCCTCCTGGAGGACCCCGGCCACCCTGAG M11: bp398-399
 PhoVit_1A6  (330) TAGCATGATGGTTATTGACATGTACTTCACCAACTGCCAGAGCCTGCTGGAGGACTC-GGCCACCCTGAG
 UrsMar_1A6  (230) TAACATGATTGTTATTGACATGTACTTCACCAACTGCCAGAGCCTCCTGGAGGACGC-GGCCACCCTGAG
 UrsThi_1A6  (229) TAACATGATTGTTATTGACATGTACTTCACCAACTGCCAGAGCCTCCTGGAGGACGC-GGCCACCCTGAG
 ProLot_1A6  (186) CAACATGATCGGTATTGACATGTACTTCACCAACTGCCAGAGCCTCCTGGAGGACTC-GGCCACCCTGAG
 CanFam_1A6  (330) TAGCATGATTGTTATTGACATGTACTTCACCAACTGCCAGAGCCTTCTGGAGGACTC-GGCCACGCTCAG
 CanRuf_1A6  (229) TAGCATGATTGTTATTGACATGTACTTCACCAACTGCCAGAGCCTTCTGGAGGACTC-GGCCACGCTCAG
 ChrBra_1A6  (229) TAGCATGATTGTTATTGACATGTACTTCACCAACTGCCAGAGCCTTCTGGAGGACTC-GGCCACGCTCAG
 VulVul_1A6  (231) TAACATGATTGTTATTGACATGTACTTCACCAACTGCCAGAGCCTTCTGGAGGACTC-GGCCACGCTCAG
 AilFul_1A6  (170) TAACATGATCGTTATTGACATGTATTTCACCAACTGCCAGAGCCTCCTGGAGGACTT-GGCCACCCTGCG
 ArcBin_1A6  (342) CACCATGATTATTCTGGACATGTGGTTCACCAGCTGCCAGAGCCTCCTGGAGGACTC-CGCCACCCTGAG
 CivCiv_1A6  (152) CACCATGATTGTTCTTGACATGTACTTCACCAACTGCCAGAGCCTCCTGGAGGACTC-CGCCACCCTGAG
 HerJav_1A6  (342) TACCATGATTGTTATTGACATGTACTTCACCAGCTGCCAGAGCCTTCTGGGGGACCC-CGCCACCCTGCG
 GalGal_1A6  (351) CATTATAGAAATTTCCAGGTTTTTTTCACCACCTGTGAGAGCCTGTTGCACAACAA-AGAAATGATGCA

                   421                                                                490
 HomSap_1A6  (411) CTTCTTTAAGGAGAGCAAGTTTGATGCTCTTTTCACAGACCCAGCCTTACCCTGTGGGGTGATCCTGGCT
 PanTro_1A6  (411) CTTCTTTAAGGAGAGCAAGTTTGATGCTCTTTTCACAGACCCAGCCTTACCCTGTGGGGTGATCCTGGCT
 PonAbe_1A6  (411) CTTCTTCAAGGAGAGCAAGTTCGATGCTCTTTTCACAGACCCAGCCTTACCCTGTGGGGTGATCCTGGCT
 PapAnu_1A6  (411) CTTCCTCAAGGAGAGCAAGTTCGATGCTCTTTTCACAGACCCAGCCTTACCCTGTGGGGTGATCCTGGCT
 MacFas_1A6  (411) CTTCCTCAAGGAGAGCAAGTTCGATGCTCTTTTCACAGACCCAGCCTTACCCTGTGGGGTGATCCTGGCT
 AciJub_1A6  (410) CTTCCTCAGGGAGAGCAAGTTCGACACCCTCTTCACAGACCCAGCCCTGCCCTGTGGGGTGATCTTGGCA
 PanTig_1A6  (310) TTTCCTCAGGGAGAGCAAGTTCGACACCCTCTTCACAGACCCAGCCCTGCCCTGTGGGGTGATCTTGGCA
 PanOnc_1A6  (310) TTTCCTCAGGGAGAGCAAGTTCGACACCCTCTTCACAGACCCAGCCCTGCCCTGTGGGGTGATCTTGGCA
 PanUnc_1A6  (310) TTTCCTCAGGGAGAGCAAGTTCGACACCCTCTTCACAGACCCAGCCCTGCCTTGTGGGGTGATCTTGGCA
 PanLeo_1A6  (310) TTTCCTCAGGGAGAGCAAGTTCAACACCCTCTTCACAGACCCAGCCCTGCCCTGTGGGGTGATCTTGGCA
 PanPar_1A6  (310) TTTCCTCAGGGAGAGCAAGTTCGACACCCTCTTCACAGACCCAGCCCTGCCCTGTGGGGTGATCTTGGCA
 LeoGeo_1A6  (410) TTTCCTCAGGGAGAGCAAGTTCGACACCCTCTTCACAGACCCAGCCCTGCCCTGTGGGGTGATCTTGGCA
 LeoTig_1A6  (410) TTTCCTCAGGGAGAGCAAGTTCGACACCCTCTTCACAGACCCAGCCCTGCCCTGTGGGGTGATCTTGGCA
 LeoWie_1A6  (410) TTTCCTCAGGGAGAGCAAGTTCGACACCCTCTTCACAGACCCAGCCCTGCCCTGTGGGGTGATCTTGGCA
 LynCan_1A6  (410) TTTCCTCAGGGAGAGCAAGTTCGACACCCTCTTCACAGACCCAGCCCTGCCCTGTGGGGTGATCTTGGCA
 LynRuf_1A6  (410) TTTCCTCAGGGAGAGCAAGTTCGACACCCTCTTCACAGACCCAGCCCTGCCCTGTGGGGTGACCTTGGCA
 ParTem_1A6  (410) TTTCCTCAGGGAGAGCAAGTTCGACACCCTCTTCACAGACCCAGCCCTGCCCTGTGGGGTGATCTTGGCA
 PumCon_1A6  (406) TTTCCTCAGGGAGAGCAAGTTCAACACCCTCTTCACAGACCCAGCCCTGCCCTGTGGGGTGATCTTGGCA
 PumCo2_1A6  (406) TTTCCTCAGGGAGAGCAAGTTCAACACCCTCTTCACAGACCCAGCCCTGCCCTGTGGGGTGATCTTGGCA
 FelCat_1A6  (410) TTTCCTCAGGGAGAGCAAGTTCGACACCCTCTTCACAGACCCAGCCCTGCCCTGTGGGGTGATCTTGGCA
 PriBen_1A6  (410) TTTCCTCAGGGAGAGCAAGTTCGACACCCTCTTCACAGACCCAGCCCTGCCCTGTGGGGTGATCTTGGCA
 CarAur_1A6  (410) TTTCCTCAGGGAGAGCAAGTTCGACACCCTCTTCACAGACCCAGCCCTGCCCTGTGGGGTGATNTTGGCA
 CarSer_1A6  (410) TTTCCTCAGGGGGAGCAAGTTCGACACCCTCTTCACAGACCCAGCCCTGCCCTGTGGGGTGATCTTGGCA
 CroCro_1A6  (411) TTTCCTCAAGGAGAGCAAATTCGACGTCCTCTTCACAGACCCAGCCCTACCCTGTGGGGTGATCTTGGCA
 ParBru_1A6  (411) CTTCCTCAAGGAGAGCAAATTCGATGTCCTCTTCACAGACCCAGCCCTGCCCTGTGGGGTGATCTTGGCA
 ProCri_1A6  (220) TTTCCTCAAGGAGAGCAAATTCGACATCCTCTTCACAGACCCGGCCCTACCCTGTGGCGTGATCCTGGCA
 HyaHya_1A6  (411) CTTCCTCAAGGAGAGCAAATTCGATGTCCTCTTCACAGACCCAGCCCTGCCCTGTGGGGTGATCTTGGCA
 BosTau_1A6  (402) CTTCCTCCGGGAGAACAAGTTCGACGCCCTGTTCACAGACCCGGCCTTACCTTGCGGGGTGATCCTGGCC
 OviAri_1A6  (408) CTTCCTCCGGGAGAGCAAGTTCGATGCCCTGTTCACAGACCCGGCCTTACCTTGCGGGGTGATCCTGGCC
 SusScr_1A6  (411) CTTCCTCCGGGAGGCCAAGTTCGATGCCCTGTTCACAGACCCGGCCTTACCCTGCGGGGTGATCCTGGCC
MusMus_1A6a  (411) CTTCCTCAGGGAGAACAAGTTTGATGCTCTGTTCACAGACCCAGCCATGCCCTGTGGTGTGATCCTGGCT
MusMus_1A6b  (411) CTTCCTCAGGGAGAACAAGTTTGATGCTCTGTTCACAGACCCAGCCATGCCCTGTGGTGTGATCCTGGCT
 RatNor_1A6  (408) CTTCCTCAGGGAGAACCAGTTTGATGCTCTGTTCACAGACCCGGCCATGCCCTGTGGTGTGATCCTGGCT
 OryCun_1A6  (411) TTTCCTCAGGGCGGGCAAGTTCGATGCTCTTTTCACAGACCCGGCCTTACCCTGTGGTGTGATCCTGGCT
EquCab_1A6a  (411) CTTCCTCAGGGAGAGCAAGTTCGATGCCCTTTTCACAGACCCGGCCTTACCCTGTGGGGTGATCCTGGCT
EquCab_1A6b  (411) CTTCCTCAGGGAGAGCAAGTTCGATGCCCTTTTCACGGACCCGGCCTTACCCTGTGGGGTGATCCTGGCT
EquCab_1A6c  (411) TTTCCTCAGGGAGAGCAAGTTCGATGCCCTTTTCACAGACCCGGCCTTACCCTGTGGGGTGATCCTGGCT
 MusNig_1A6  (399) TCTCCTCAGGGAGAGCAAGTTCGATGCCCTTTTCACAGACCCAGCCCTACCCTGCGGGGTGATCCTGGCT
 MusPut_1A6  (399) TGTCCTCAGGGAGAGCAAGTTCGATGCCCTTTTCACAGACCCAGCCCTACCCTGCGGGGTGATCCTGGCT
 MirAng_1A6  (400) TGTCCTCAGGGAGAGCAAGTTCGATGCCCTTTTCACAGAACCAGCCCTACCCTGCGGGGTCATCCTGGCC
 PhoVit_1A6  (399) TGTCCTCAGGGAGAGCAAGTTCGATGCCCTTTTCACAGACCCAGCCCTACCCTGCGGGGTCATCCTGGCC
 UrsMar_1A6  (299) CGTCCTCCGGAAGAGCAAGTTCGATGCCCTTTTCACAGACCCGGCCCTACCCTGCGGGGTGATCCTGGCC
 UrsThi_1A6  (298) CGTCCTCCGGAAGAGCAAGTTCGATGCCCTTTTCACAGACCCGGCCCTACCCTGCGGGGTGATCCTGGCC
 ProLot_1A6  (255) CGTCCTCAGGGAGAGCAAGTTCGATGCCCTTTTCACAGACCCAGCCCTACCCTGCGGGGTGATCCTGGCC
 CanFam_1A6  (399) TGTCCTCAGGCAGAGCAAGTTCGATGCCCTTTTCACAGACCCAGCTCTGCCCTGTGGGGTGATCCTGGCC
 CanRuf_1A6  (298) TGTCCTCAGGCAGAGCAAGTTCGATGCCCTTTTCACAGACCCAGCTCTGCCCTGTGGGGTGATCCTGGCC
 ChrBra_1A6  (298) TGTCCTCAGGCAGAGCAAATTCGATGCCCTTTTCACAGACCCAGCTCTGCCCTGTGGGGTGATCCTGGCC
 VulVul_1A6  (300) TGTCCTCAGGCAGAGCAAGTTCGATGCCCTTTTCACAGACCCAGCTCTGCCCTGTGGGGTGATCCTGGCC
 AilFul_1A6  (239) TGTCCTCAGGGAGAGCAAGTTCGATGCCCTTTTCACAGACCCAGCCCTACCCTGCGGGGTGATCCTGGCC
 ArcBin_1A6  (411) TTTCCTCAGGGAGAGCAAGTTCGACGCCCTCTTCACAGACCCGGCCCTCCCCTGTGGGGTGATCTTGGCA
 CivCiv_1A6  (221) TTTCCTCAGGGAGAGCAAGTTCGACGCCCTCTTCACAGACCCAGCCCTCCCTTGTGGGGTGATCTTGGCA
 HerJav_1A6  (411) CTTCCTCCGGGAGAGCAAGTTCGACGCCCTCTTCACAGACCCAGCTCTACCCTGTGGGGTGATCTTGGCG
 GalGal_1A6  (420) GTATTTGAGAAAGAGCAAATTTGACGTTGTTTTCACAGATCCAATTCTGATGTGTGGACCAATACTTGCT

                   491                                                                560
 HomSap_1A6  (481) GAGTATTTGGGCCTACCATCTGTGTACCTCTTCAGGGGTTTTCCGTGTTCCCTGGAGCATACATTCAGCA
 PanTro_1A6  (481) GAGTATTTGGGCCTACCATCTGTGTACCTCTTCAGGGGTTTTCCGTGTTCCCTGGAGCATACATTCAGCA
```

```
  PonAbe_1A6  (481) GAGTATTTGGGCCTACCCTCCGTGTACCTCTTCAGGGGTTTTCCGTGTTCCCTCGAGCATACATTCAGCA
  PapAnu_1A6  (481) GAGTATTTGGGCCTACCCTCCGTGTACCTCTTCAGGGGTTTTCCGTGTTCCCTGGAGCATACATTCAGCA
  MacFas_1A6  (481) GAGTATTTGGGCCTACCCTCCGTGTACCTCTTCAGGGGTTTTCCGTGTTCCCTGGAGCATACATTCAGCA
  AciJub_1A6  (480) GAGTACCTAGGCCTGCCCTCCATGTATCTCTTCAGGGGCTTCCCACGCTCCTTGGGGCACGCAATCAGCC
  PanTig_1A6  (380) GAGTACCTAGGCCTGCCCTCCATGTATCTCTTCAGGGGCTTCCCACGCTCCCTGGGGCACGCGATCAGCC
  PanOnc_1A6  (380) GAGTACCTAGGCCTGCCCTCCATGTATCTCTTCAGGGGCTTCCCACGCTCCCTGGGGCACGCGATCAGCC
  PanUnc_1A6  (380) GAGTACCTAGGCCTGCCCTCCATGTATCTCTTCAGGGGCTTCCCACGCTCCCTGGGGCACGCGATCAGCC
  PanLeo_1A6  (380) GAGTACCTAGGCCTGCCCTCCATGTATCTCTTCGGGGGCTTCCCACGCTCCCTGGGGCACGCGATCAGCC
  PanPar_1A6  (380) GAGTACCTAGGCCTGCCCTCCATGTATCTCTTCAGGGGCTTCCCACGCTCCCTGGGGCACGCGATCAGCC
  LeoGeo_1A6  (480) GAGTACCTAGGCCTGCCCTCCATGTATCTCTTCAGGGGCTTCCCACGCTCCCTGGGGCACGCAATCAGCC
  LeoTig_1A6  (480) GAGTACCTAGGCCTGCCCTCCATGTATCTCTTCAGGGGCTTCCCACGCTCCCTGGGGCACGCAATCAGCC
  LeoWie_1A6  (480) GAGTACCTAGGCCTGCCCTCCATGTATCTCTTCAGGGGCTTCCCATGCTCCCTGGGGCACGCAATCAGCC
  LynCan_1A6  (480) GAGTACCTAGGCCTGCCCTCCATGTATCTTTTCAGGGGCTTCCCACGCTCCCTGGGGCACGCGATCAGCC
  LynRuf_1A6  (480) GAGCACCTAGGCCTGCCCTCCATGTATCTTTTCAGGGGCTTCCCACGCTCCCTGGGGCACGCGATCACCC
  ParTem_1A6  (480) GAGTACCTAGGCCTGCCCTCCATGTATCTTTTCAGGGGCTTCCCACGCTCCCTGGGGCACGCGATCAGCC
  PumCon_1A6  (476) GAGTACCTAGGCCTGCCCTCCATGTATCTCTTCAGGGGCTTCCCACGCTCCTTGGGGCACACAATCAGCC
  PumCo2_1A6  (476) GAGTACCTAGGCCTGCCCTCCATGTATCTCTTCAGGGGCTTCCCACGCTCCTTGGGGCACACAATCAGCC
  FelCat_1A6  (480) GAGTACCTAGGCCTGCCCTCCATGTATCTCTTCAGGGGCTTCCCACGCTCCTTGGGGCACGCGATCAGCC
  PriBen_1A6  (480) GAGTACCTAGGCCTGCCCTCCATGTATCTCTTCAGGGGCTTCCCACGCTCCTTGGGGCACGCGATCAGCC
  CarAur_1A6  (480) GAGTACCTAGGCCTGCCCTCCATGTGTCTCTTCAGGGGCTTCCCACGCTCCCTGGGGCACGCGATCAGCC
  CarSer_1A6  (480) GAGTACCTAGGCCTGCCCTCCATGTGTCTCTTCAGGGTCTTCCCACGCTCCCTGGGGCACGCGATCAGCC
  CroCro_1A6  (481) GAGTACCTGGGCCTGCCCTCCGTATATCTCTTCAGGGGCTTCCCATGCTCCTTGGAGCACGCGATCAGCA
  ParBru_1A6  (481) GAGTACCTGGGCCTGCCCTCCGTATATCTCTTCAGGGGCTTCCCATGCTCCTTGGAGCACGTGATCAGCA
  ProCri_1A6  (290) GAGTACCTGGGCCTGCCCTCCGTATATCTCTTCAGGGGCTTCCCATGCTCCTTGGAGCACGCGATCAACA
  HyaHya_1A6  (481) GAGTACCTGGGCCTGCCCTCCGTATATCTCTTCAGGGGCTTCCCATGCTCCTTGGAGCACGTGATCAGCA
  BosTau_1A6  (472) GAGTACCTGAACCTGCCCTCCGTGTATCTCTTCAGGGGCTTCCCCTGTGCCCTGGAGAACACTTTCACCA
  OviAri_1A6  (478) GAGTACCTGAACCTGCCCTCTGTGTATCTCTTCAGGGGCTTTCCCTGTGCCCTGGAGAACACGTTCACCA
  SusScr_1A6  (481) GAGTACCTGGGCCTGCCGTCCGTGTACCTCTTCAGGGGCTTCCCGTGCTCCCTGGAGAACACGTTCACCA
 MusMus_1A6a  (481) GAGTATCTCAACCTGCCCTCTGTCTACCTCTTCAGAGGTTTCCCATGCTCTCTGGAACACATGCTTGGTC
 MusMus_1A6b  (481) GAGTATCTCAACCTGCCCTCTGTCTACCTCTTCAGAGGTTTCCCATGCTCTCTGGAACACATGCTTGGTC
  RatNor_1A6  (478) GAGTATCTCAAGCTGCCTTCCATCTACCTCTTCAGAGGTTTCCCATGCTCTCTGGAGCACATGCTTGGTC
  OryCun_1A6  (481) GAGTACCTGGGCCTGCCCTCCGTGTACCTGTTCAGGGGCTTCCCCTGTTCCCTGGAGCATGGGTTTGGCG
 EquCab_1A6a  (481) GACTACCTGGGCCTGCCCTCCGTGTACCTCTTCAGGGGCTTCCCGTGTTCCCTGGAGTTTGCGTTCACTA
 EquCab_1A6b  (481) GAGTACCTGGGCCTGCCCTCCGTGTACCTCTTCAGGGGCTTCCCATGTTCCCTGGAGTATGCGTTCACCA
 EquCab_1A6c  (481) GAGTACCTGGGCCTGCCCTCTGTGTACCTCTTCAGGGGTTTCCCATGTTCCCTGGAGTATGCATTCACCA
  MusNig_1A6  (469) GAGTACCTGGGCCTGCCCTCCGTGTACCTCTTCAGGGGCTTCCCATGCTCCCTGGAGCATACCATGAGCA
  MusPut_1A6  (469) GAGTACCTGGGCCTGCCCTCCGTGTACCTCTTCAGGGGCTTCCCATGCTCCCTGGAGCATACCATGAGCA
  MirAng_1A6  (470) GAGTACCTGAGCCTGCCCTCTGTGTACCTCTTCGGGGGCTTCCCATGCTCCCTGGAGCATATGATGAGCA
  PhoVit_1A6  (469) GAGTACCTGGGCCTGCCCTCCGTGTACCTCTTCAGGGGCTTCCCATGCTCCCTGGAGCATATGATGAGCA
  UrsMar_1A6  (369) GAGTACCTGGGCCTGCCCTCCGTGTACCTCTTCAGGGGCTTCCCATGCTCCCTGGAGCATACGATGAGCA
  UrsThi_1A6  (368) GAGTACCTGGGCCTGCCCTCCGTGTACCTCTTCAGGGGCTTCCCATGCTCCCTGGAGCATACGATGAGCA
  ProLot_1A6  (325) GAGTACCTGGGCCTGCCCTCCGTGTACCTCTTCAGGGGTTTCCCGTGCTCCCTGGAGCATGCCATGAGCA
  CanFam_1A6  (469) GAGTACCTGGGCCTGCCCTCCGTGTACCTCTTCAGGGGCTTCCCATGCTCCCTGGAGCATACTATCAGCA
  CanRuf_1A6  (368) GAGTACCTGGGCCTGCCCTCCGTGTACCTCTTCAGGGGCTTCCCATGCTCCCTGGAGCATACTATCAGCA
  ChrBra_1A6  (368) GAGTACCTGGGCCTGCCCTCCGTGTACCTCTTCAGGGGCTTCCCATGCTCCCTGGAGCATACTATCAGCA
  VulVul_1A6  (370) GAGTACCTGGGCCTGCCCTCCGTGTACCTCTTCAGGGGCTTCCCATGCTCCCTGGAGCATACTATCAGCA
  AilFul_1A6  (309) GAGTACCTGGGCCTGCCCTCCGTATACCTCTTCAGGGGTTTCCCATGCTCCCTGGAGCATGCGATGAGCA
  ArcBin_1A6  (481) GAGTACCTGGGCCTGCCCTCCGTGTACCTCTTCAGGGGCTTCCCGTGCTCCCTGGAGCACGCCATCAGCA
  CivCiv_1A6  (291) GAGTACCTGGGCCTGCCCTCTGTGTATCTCTTCAGGGGCTTCCCATGCTCCCTGGAGCACGCGATCAGCA
  HerJav_1A6  (481) GAGTACCTGGGCCTGCCCTCTGTGTATCTCTTCAGGGGCTTCCCGTGCTCCCTGGAGCACGCGATCAGCA
  GalGal_1A6  (490) GATTATCTTTCAGTTCCTTCTGTCTACTTCTTGCGAGGATTTCCTTGTGGTATGGATTCAAAAGCTACCC

                    561                                                                 630
  HomSap_1A6  (551) GAAGCCCAGACCCTGTGTCCTACATTCCCAGGTGCTACACAAAGTTTTCAGACCACATGACTTTTTCCCA
  PanTro_1A6  (551) GAAGCCCAGACCCTGTGTCCTACATTCCCAGGTGCTACACAAAGTTTTCAGACCACATGACTTTTTCCCA
  PonAbe_1A6  (551) GAAGCCCAGACCCTGTGTCCTACATTCCCAGGTGCTACACAAAGTTTTCAGACCACATGACTTTTTCCCA
  PapAnu_1A6  (551) GAAGCCCAAACCCTGTGTCCTATATTCCCAGGTGCTACACAAAGTTTTCAGACCACATGACTTTTCCCCA
  MacFas_1A6  (551) GAAGCCCAAACCCTGTGTCCTATATTCCCAGGTGCTACACAAAGTTTTCAGACCACATGACTTTTCCCCA
  AciJub_1A6  (550) AGAGCCCAAACCCCGCATCCTACATTCCCAGATGCTACACTCAGTTCTCAGACCGGATGACCTTCCCCCA
  PanTig_1A6  (450) AGAGCCCGAACCCCGCATCCTACATTCCCAGATGCTACA-TCAGTTCTCAGACCGGATGACCTTCCCCCA
  PanOnc_1A6  (450) AGAGCCCGAACCCCGCATCCTACATTCCCAGATGCTACACTCAGTTCTCAGACCGGATGACCTTCCCCCA
  PanUnc_1A6  (450) AGAGCCCGAACCCCGCATCCTACATTCCCAGATGCTACACTCAGTTCTCAGACCGGATGACCTTCCCCCA
  PanLeo_1A6  (450) AGAGCCCGAACCCCGCATCCTACATTCCCAGATGCTACACTCAGTTCTCAGACCGGATGACCTTCCCCCA
  PanPar_1A6  (450) AGAGCCCGAACCCCGCATCCTACATTCCCAGATGCTACACTCAGTTCTCAGACCGGATGACCTTCCCCCA
  LeoGeo_1A6  (550) AGAGCCCAAACCCTGCATCCTACATTCCCAGATGCTACACTCAGTTCTCAGACCGGATGACCTTCCCCCA
  LeoTig_1A6  (550) AGAGCCCAAACCCTGCATCCTACATTCCCAGATGCTACACTCAGTTCTCAGACCGGATGACCTTCCCCCA
  LeoWie_1A6  (550) AGAGCCCAAACCCTGCATCCTACATTCCCAGATGCTACGCTCAGTTCTCAGACCGGATGACCTTCCCCCA
  LynCan_1A6  (550) AGAGCCCAAACCCCGCATCCTACATTCCCAGATGCTACACTCAGTTCTCACACCGGATGACCTTCCCCCA
  LynRuf_1A6  (550) AGAGCCCGAACCCCGCATCCTACGTTCCCAGATGCTACACTCAGTTCTCAGACCGGATGACCTTCCCCCA
  ParTem_1A6  (550) AGAGCCCAAACCCCGCATCCTACATTCCCAGATGCTACACTCAGTTCTCAGACCAGATGACCTTCCCCCA
  PumCon_1A6  (546) AGAGCCCAAACCCCGCATCCTACATTCCCAGATGCTATACTCAGTTCTCAGACCGGATGACCTTCCCCCA
  PumCo2_1A6  (546) AGAGCCCAAACCCTGCATCCTACATTCCCAGATGCTATACTCAGTTCTCAGACCGGATGACCTTCCCCCA
  FelCat_1A6  (550) AGAGCCCAAACCCCGCATCCTACATTCCCAGATGCTACACTCAGTTCTCAGACCGGATGACCTTCCCCCA
  PriBen_1A6  (550) AGAGCCCAAACCCCGCATCCTACATTCCCAGATGCTACACTCAGTTCTCAGACCGGATGACCTTCCCCCA
  CarAur_1A6  (550) AGAGCCCAAACCCCGCATCCTACATTCCCAGATGCTACACTCAGTTCTCAGACCGGATGACCTTCCCCCA
  CarSer_1A6  (550) AGAGCCCAAACCCCGCATCCTACATTCCCAGATGCTACACTCAGTTCTCAGACCGGATGACCTTCCCCCA
  CroCro_1A6  (551) GAAGCCCAAACCCTGGGTCCTACATTCCCAGATGCTACACTCAGTTCTCAGACCGGATGACCTTCCCCCA
  ParBru_1A6  (551) GAAGCCCAAACCCTGGGTCCTACATTCCCAGATGCTACACTCAGTTCTCAGACCGGATGACCTTCCCCCA
```

```
  ProCri_1A6  (360) GAAGCCCAAACCCTGGGTCCTACATTCCCAGATGCTACACTCAGTTCTCAGACCGGATGACCTTCCCCCA
  HyaHya_1A6  (551) GAAGCCCAAACCCTGGGTCCTACATTCCCAGATGCTACACTCAGTTCTCAGACCGGATGACCTTCCCCCA
  BosTau_1A6  (542) GGACTCCGAGCCCCTTGTCCTACGTCCCCAGGTACTACACTCAGTTCTCAGACCACATGACCTTCCTCCA
  OviAri_1A6  (548) GGACTCCAAGCCCTTTGTCCTACGTCCCCAGGTACTACACTCAGTTTTCAGACAAGATGACCTTCCTCCA
  SusScr_1A6  (551) GGACCCCAAGCCCCACGTCCTACGTCCCCAGGTACTACACTGCGTTCTCCGACCGGATGACTTTCCCCCA
 MusMus_1A6a  (551) AAAGCCCAAGCCCTGTGTCCTATGTGCCCAGATTCTACACCAAATTCTCAGACCACATGACGTTTCCCCA
 MusMus_1A6b  (551) AAAGCCCAAGCCCTGTGTCCTATGTTCCCAGATTCTACACCAAATTCTCAGACCACATGACGTTTCCCCA
  RatNor_1A6  (548) AAAGCCCAAGCCCCGTATCCTATGTTCCCAGATTCTACACCAAATTCTCAGACCACATGACATTTCCCCA
  OryCun_1A6  (551) GAAGCCCCAACCCGGTGTCGTACATCCCCCGGTGCTACACAAAGTTCTCCGACCAGATGAGCTTCCCCCA
 EquCab_1A6a  (551) GAAGTCCAAACCCGGTGTCCTACGTTCCCACATGCTACACCCAGTTCTCAGACCAGATGACTTTCCCCCA
 EquCab_1A6b  (551) GAAGCCCAAACCCGGTGTCCTACACTCCCAGGTGCTACACCCAGTTCTCAGACAGGATGATTTTCCCCCA
 EquCab_1A6c  (551) GAAGCCCAAACCCAGTGTCCTACACTCCCAGGTGCTATACCCAGTTCTCAGACAGGATGACTTTCCCCCA
  MusNig_1A6  (539) GAAGCCCAAACCCTGTGTCCCACATTCCCAGATGCTACACTCAGTTCTCAGACCAGATGACGTTCCCCCA
  MusPut_1A6  (539) GAAGCCCAAACCCTGTGTCCCACATTCCCAGATGCTACACTCAGTTCTCAGACCAGATGACGTTCCCCCA
  MirAng_1A6  (540) GGAGCCCAAACCCTGTGTCCCATATTCCCAGATGCTACACTCAGTTCTCAGACCAGATGACGTTCCCCCA
  PhoVit_1A6  (539) GGAGCCCAAACCCTGTGTCCCACATTCCCAGCTGCTACACTCAGTTCTCAGACCAGATGACGTTCCCCCA
  UrsMar_1A6  (439) GGAGCCCGAACCCCGTGTCCTACATTCCCAGATGCTACACTCAGTTCTCAAACCGGATGACATTCCCCCA
  UrsThi_1A6  (438) GGAGCCCGAACCCCGTGTCCTACATTCCCAGATGCTACACTCAGTTCTCAAACCGGATGACATTCCCCCA
  ProLot_1A6  (395) GGACCCCGAACCCCGTGTCCTACATTCCCAGATGCTACACTCAGTTCTCAAACCCGATGACGTTCCCCCA
  CanFam_1A6  (539) GGAGCCCAAACCCTGTGTCCTACATTCCCAGGTGCTATACTCAGTTCTCAGACAAGATGACATTTCCCCA
  CanRuf_1A6  (438) GGAGCCCAAACCCTGTGTCCTACATTCCCAGGTGCTATACTCAGTTCTCAGACAAGATGACATTTCCCCA
  ChrBra_1A6  (438) GGAGCCCAAACCCTGTGTCCTACATTCCCAGGTGCTATACTCAGTTCTCAGACAAGATGACATTTCCTCA
  VulVul_1A6  (440) GGAGCCCAAACCCTGTGTCCTACATTCCCAGGTGCTATACTCAGTTCTCAGACAAGATGACATTTCCCCA
  AilFul_1A6  (379) GGAGTCCAAACCCCGTGTCCTACATTCCCAGATGCTACACTCAGTTCTCAGACCGGATGACGTTCCCCCA
  ArcBin_1A6  (551) GGAGCCCAAACCCTGTGTCCTACGTTCCCAGATGCTACACTAAGTTCTCAGACCGGATGACCTTCCCCCA
  CivCiv_1A6  (361) GGAGCCCAAACCCTGTGTCCCACATTCCCAGGTGCTACACTCAGTTCTCAGACCGGATGACCTTCCCCCA
  HerJav_1A6  (551) GGAGCCCAAACCCTGTGTCCTACATTCCCAGATGCTACACTCAGTTCTCAGACCGGATGACCTTCCCCCA
  GalGal_1A6  (560) AGTGTCCAAGCCCTCCTTCTTATGTTCCCAGGCTCTTCCTAAATAATTCAGATAGCATGACATTTGCTCA

                    631                                                                 700
  HomSap_1A6  (621) ACGAGTGGCCAACTTCCTTGTTAATTTGTTGGAGCCCTATCTATTTTATTGTCTGTTTTCAAAGTATGAA
  PanTro_1A6  (621) ACGAGTGGCCAACTTCCTTGTTAATTTGTTGGAGCCCTATCTATTTTATTGTCTGTTTTCAAAGTATGAA
  PonAbe_1A6  (621) ACGAGTGGCCAACTTCCTTGTTAATTTGTTGGAGCCATATCTATTTTATTGTCTGTTTTCAAAGTATGAA
  PapAnu_1A6  (621) ACGAGTGGCCAACTTCCTCGTTAATTTGTTGGAGCCCTATCTATTTTATTGTCTGTTTTCAAAGTACGAC
  MacFas_1A6  (621) ACGAGTGGCCAACTTCCTCGTTAATTTGTTGGAGCCCTATCTATTTTATTGTCTGTTTTCAAAGTACGAT
  AciJub_1A6  (620) ACGGGTGGCCAACTACCTCGTTCGTTACTTGGAGACCTA----------CCTTTGTATTCAAAGTATGAA
  PanTig_1A6  (519) ACGGGTGGCCAACTACCTCGTTCATTACTTGGAGACCTA----------CCTTCGTATTCAAAGTATGAA
  PanOnc_1A6  (520) ACGGGTGGCCAACTACCTCGTTCATTACTTGGAGACCTA----------CCTTCGTATTCAAAGTATGAA
  PanUnc_1A6  (520) ACGGGTGGCCAACTACCTCGTTCATTACTTGGAGACCTA----------CCTTCGTATTCAAAGTATGAA
  PanLeo_1A6  (520) ACGGGTGGCCAACTACCTCGTTCATTACTTGGAGACCTA----------CCTTCATATTCAAAGTATGAA
  PanPar_1A6  (520) ACGGGTGGCCAACTACCTCGTTCATTACTTGGAGACCTA----------CCTTCGTATTCAAAGTATGAA
  LeoGeo_1A6  (620) ACGGGTGGCCAACTACCTCGTTCATTACTTGGAGACCTA----------CCTTCGTATTCAAAGTATGAA
  LeoTig_1A6  (620) ACGGGTGGCCAACTACCTCGTTCATTACTTGGAGACCTA----------CCTTCGTATTCAAAGTATGAA
  LeoWie_1A6  (620) ACGGGTGGCCAACTACCTCGTTCATTACTTGGAGACCTA----------CCTTCGTATTCAAAGTATGAA
  LynCan_1A6  (620) ATGGGTGGCCAACTACCTCGTTCATTACTTGGAGACCTA----------CCTTCGTATTCAAAGTATGAA M4: bp660-669
  LynRuf_1A6  (620) GTGGGTGGCCAACTACCTCGTTCATTACTTGGAGACCTA----------CCTTCGTATTCAAAGTATGAA
  ParTem_1A6  (620) ACGGGTGGCCAACTATCTTGTTCATTACTTGGAGACCTA----------CCTTCGTATTCAAAGTATGAA
  PumCon_1A6  (616) ACGGGTGGCCAACTACCTCGTTCGTTACTTGGAGGCCTA----------CCTTCGTATTCAAACTATGAA
  PumCo2_1A6  (616) ACGGGTGGCCAACTACCTCGTTCGTTACTTGGAGGCCTA----------CCTTCGTATTCAAAGTATGAA
  FelCat_1A6  (620) ACGGGTGGCCAACTACCTCGTTCGTTACTTGGAGACCTA----------CCTTCGTATTCAAAGTATGAA
  PriBen_1A6  (620) ACGGGTGGCCAACTACCTCGTTCGTTACTTGGAGACCTA----------CCTTCGTATTCAAAGTATGAA
  CarAur_1A6  (620) ACGGGTGGCCAACTCCCTCGTTCATTACTTGGAGACCTA----------CCTTCGTATTCAAAGTATGAA
  CarSer_1A6  (620) ACGGGTGGCCAACTCCCTCGTTCATTACTTGGAGACCTA----------CCTTCGTATTCAAAGTATGAA
  CroCro_1A6  (621) ACGGGTGGCCAACTACCTCGTTAGTTACTTGGAGACCTATTTATTCTACCTTCTGTATTCAAAGTATGAA
  ParBru_1A6  (621) ACGGGTGGCCAACTACCTCGTTAGTTACTTGGAGACCTATTTATTCTACCTTCTATATTCAAAGTATGAA
  ProCri_1A6  (430) ACGGGTGGTCAACTACCTCGTTAGTTACTTGGAGACCTATTTATTCTACCTTCTGTATTCAAAGTACGAA
  HyaHya_1A6  (621) ACGGGTGGCCAACTACCTCGTTAGTTACTTGGAGACCTATTTATTCTACCTTCTATATTCAAAGTATGAA
  BosTau_1A6  (612) AAGGGTGGGCAATTTCCTGGTGAACTACCTGGAGAACATTCTGCTCTACGCGCTGTATTCCAAGTACGAA
  OviAri_1A6  (618) AAGGGTGGCCAACTTCCTGGTGAGTTACCTGGAGAACATTTTGCTCTATGCGCTGTATTCCAAGTACGAA
  SusScr_1A6  (621) ACGGGTGGCCAACTTCCTTACTAGGATCTTGGAGAATATCCTCCTTGACCTGCTGTATACTAAGTACGAA
 MusMus_1A6a  (621) ACGGCTGGCCAACTTCATTGTTAACATCTTGGAAAACTACCTATATTATTGTCTGTATTCAAAGTATGAA
 MusMus_1A6b  (621) ACGGCTGGCCAACTTCATTGTTAACATCTTGGAAAACTACCTATATTATTGTCTGTATTCAAAGTATGAA
  RatNor_1A6  (618) ACGGCTGGCCAACTTCATTGCTAACATCTTGGAGAACTACCTTTATCATTGTCTGTACTCAAAGTATGAG
  OryCun_1A6  (621) GCGCGTGGTCAACTTCCTCGTTAACTTGTTGGAGGTCCCTCTATTTTACTGTCTGTATTCAAAGTATGAG
 EquCab_1A6a  (621) ACGTGTGGCCAACTTCCTCGCTAATTACTTGAAGGACTACCTATTTTACTGTCTGTATTCCAAGTATGAA
 EquCab_1A6b  (621) ACGTGTGGCCAACTTCCTCGTTAGTTACTTGGAGGACTACCTATTTTACTGTCTGTATTCAAAGTATGAA
 EquCab_1A6c  (621) ACGTGTGGCCAACTTCCTCGTTAGTTACTTGGAGAAACTCCTATTTTACTGTCTGTATTCAAAGTATGAA
  MusNig_1A6  (609) ACGGGTGGCCAACTACCTCGTTAATTACTTGGAGACCTATCTGTTCCACTGTCTGTACTCGAAGTACGAA
  MusPut_1A6  (609) ACGGGTGGCCAACTACCTCGTTAATTACTTGGAGACCTATCTGTTCCACTGTCTGTACTCGAAGTACGAA
  MirAng_1A6  (610) ACGGGTGGCCAACTACCTCGTTAATTACTTGGAGACCTATCTGTTCTAATGCCTGTATTCAAAGTATGAA M12: bp667-669
  PhoVit_1A6  (609) ACGGGTGGCCAACTACCTCGTTAATTCCTTGGAGACCTATCTGTTCTGCTGCCTGTATTCAAAGTATGAA
  UrsMar_1A6  (509) AAGGGTGGCCAACTACCTCGTTAATTACTTGGAGACCTATCTGTTCTACTGTCTGTATTCCAAGTATGAA
  UrsThi_1A6  (508) AAGGGTGGCCAACTACCTCGTTAATTACTTGGAGACCTATCTGTTCTACTGTCTGTATTCCAAGTATGAA
  ProLot_1A6  (465) ACGGGTGGCCAACTACCTTGCTAATTACCTGGAGACTTATCTGTTCCACTGTCTGTACTCAAAGTACGAA
  CanFam_1A6  (609) ACGGGTGGGCAACTACCTCGTTAATTACCTGGAGACCTACCTGTTCTACTGTCTGTATTCAAAGTACGAA
  CanRuf_1A6  (508) ACGGGTGGGCAACTACCTTGTTAATTACCTGGAGACCTACCTGTTCTACTGTCTGTATTCAAAGTACGAA
  ChrBra_1A6  (508) ACGGGTGGGCAACTACCTCGTTAATTACCTGGAGACCTACCTGTTCTACTGTCTGTATTCAAAGTACGAA
  VulVul_1A6  (510) ACGGGTGGGCAACTACCTCGTTAATTACCTGGAGACCTACCTGTTCTACTGTCTGTATTCAAAGTACGAA
```

```
   AilFul_1A6   (449) ACGGGTGGCCAACTTCCTCGTTAATTACTTAGAGACCTACCTGTTCTACTGTCTGTACTCAAAGTACGAA
   ArcBin_1A6   (621) GCGGGTGGCCAACTACCTCGTTAATAACTTGGAGACCTATATATTCCACCTTCTGTATTCAAAGTATGAA
   CivCiv_1A6   (431) GCGGGTGGCCAACTACCTCGTTAATTACTTGGAGACCTATATATTTTACCTTCTGTATTCAAAGTATGAA
   HerJav_1A6   (621) ACGGGTGGCCAACTACCTTGTTAGTTACTTGGAGACCTACATATTCTACCTTCTGTATTCAAAGTATGAA
   GalGal_1A6   (630) ACGCATGAAAAACATGCTGGTCAGTATACTGGAGCTTGTTTACTGTCAGCCTTTATTCACTCAGTTTGAA

                      701                                                                  770
   HomSap_1A6   (691) --GAACTCGCATCAGCTGTCCTCAAGAGAGATGTGGATATAATCACCTTATATCAGAAGGTCTCTGTTTG
   PanTro_1A6   (691) --GAACTCGCATCAGCTGTCCTCAAGAGAGATGTGGATATAATCACCTTATATCAGAAGGTCTCTGTTTG
   PonAbe_1A6   (691) --GAACTTGCATCAGCTGTCCTCAAGAGAGATGTGGATATAATCACCTTATATCAGAAGGTCTCTATTTG
   PapAnu_1A6   (691) --GAACTCGCATCAGCTGTCCTCAAGAGAGATGTGGATGTAATCACCTTGTATCAGAAGGTCTCTGTTTG
   MacFas_1A6   (691) --GAACTCGCATCAGCTGTCCTCAAGAGAGATGTGGATGTAATCACCTTATATCAGAAGGTCTCTGTTTG
   AciJub_1A6   (680) --GACCTTGCATCCAATATCCTCAAGAGAGATGTGCNCTTACCCACCTTGTATCGGAAGGGCTCCGTCTG
   PanTig_1A6   (579) --GACCCTGCATCCAATATCCTCAAGAGAGATGTGCACTTACCCACCTTGTATCGGAAGGGCTCCGTCTG
   PanOnc_1A6   (580) --GACCCTGCATCCAATATCCTCAAGAGAGATGTGCACTTACCCACCTTGTATCGGAAGGGCTCCGTCTG
   PanUnc_1A6   (580) --GACCCTGCATCCAATATCCTCAAGAGAGATGTGCACTTACCCACCTTGTATCGGAAGGGCTCCGTCTG
   PanLeo_1A6   (580) --GACCCCGCATCCAATATCCTCAAGAGAGATGTGCACTTACCCACCTTGTATCGGAAGGGCTCCGTCTG
   PanPar_1A6   (580) --GACCCCGCATCCAATATCCTCAAGAGAGATGTGCACTTACCCACCTTGTATCGGAAGGGCTCCGTCTG
   LeoGeo_1A6   (680) --GACCTTGCATCCAATATCCTCAAGAGAGATGTGCACTTACTCGCCTTGTATCGGAAGGGCTCCGTCTG
   LeoTig_1A6   (680) --GACCTTGCATCCAATATCCTCAAGAGAGATGTGCACTTACTCGCCTTGTATCGGAAGGGCTCCGTCTG
   LeoWie_1A6   (680) --GACCTTGCATCCAATATCCTCAAGAGAGATGTGCACTTACCCACCTTGTGTCGGAAGGGCTCCGTCTG
   LynCan_1A6   (680) --GACCTTGCATCCAATATCCTCAAGAGAGATGTGCACTTACCCACCTTGTATCGGAAGGGCTCCGTCTG
   LynRuf_1A6   (680) --GACCTTGCATCCAACATCCTCAAGAGAGATGTGCACTTACCCACCTTGTATCGGAAGGGCTCCGTCTG
   ParTem_1A6   (680) --GACCTTGCATCCAATATCCTCAAGAGAGATGTGCACTTATCCACCTTGCATCGGAAGGGCTCCATCTG
   PumCon_1A6   (676) --GACCTTGCATCCAATATCCTCAAGAGAGACGTGCACTTACCCACCTTGTATCGGAAGGGCTCCGTCTG
   PumCo2_1A6   (676) --GACCTTGCATCCAATATCCTCAAGAGAGATGTGCACTTACCCACCTTGTATCGGAAGGGCTCCGTCTG
   FelCat_1A6   (680) --GGCCTTGCATCCAATATCCTCAAGAGAGATGTGCACTTACCCACCTTGTATCGGAAGGGCTCCGTCTG
   PriBen_1A6   (680) --GGCCTTGCATCCAATATCCTCAAGAGAGATGTGCACTTACCCACCTTGTATCGGAAGGGCTCCGTCTG
   CarAur_1A6   (680) --GACCTTGCATCCAATATCCTCAAGAGAGATGTGCACTTACCCACCTTGTATCGGAAGGGCTCCGTCTG
   CarSer_1A6   (680) TGGACCTTGCATCCAATATCCTCAAGAGAGATGTGCACTTACCCACCTTGTATCGGAAGGGCTCCGTCTG M8: bp691-692
   CroCro_1A6   (691) --GACCTCGCATCCAATATCCTCAAGAGAGATGTGCACTTACCCACCTTGTATCGGAAGGGCTCCATTTG
   ParBru_1A6   (691) --GACCTCGCATCCAATATCCTCAAGAGAGATGTGCACTTACCCACCTTGTATCGGAAGGGCTTCATTTG
   ProCri_1A6   (500) --GACCTCGCATCCAATATCCTCAAGAGAGATGTGCACTTACCCACCTTGTATCGGAAGGGCTCCATTTG
   HyaHya_1A6   (691) --GACCTCGCATCCAATATCCTCAAGAGAGATGTGCACTTACCCACCTTGTATCGCAAGGGCTCCATTTG
   BosTau_1A6   (682) --GACCTGGCAGGGGAAGTCCTTGGGAGACAGGTGCACTTGCCGGCCTTGTATCGGAAGGCCTCCATTTG
   OviAri_1A6   (688) --GACCTGGCGGAGGAAGTCCTTGGGAGACAGGTGCACTTGCCGGCCTTGTATCAGAAGGCCTCCATTTG
   SusScr_1A6   (691) --GACCTCGCACGTGATATCCTCAAGATAGAGGTGGACCTACCCGCCTTATATCGCAAGGCTGACATTTG
 MusMus_1A6a   (691) --ATCATTGCCTCAGACCTCCTCAAGAGAGATGTGTCCCTACCTTCCTTACACCAGAA---TTCTCTGTG
 MusMus_1A6b   (691) --ATCATTGCCTCAGACCTCCTCAAGAGAGATGTGTCCCTACCTTCCTTACACCAGAA---TTCTCTGTG
   RatNor_1A6   (688) --ATCCTTGCCTCAGACCTCCTCAAGAGAGATGTGTCCCTACCTGCCTTACACCAGAA---CTCTCTGTG
   OryCun_1A6   (691) --GACCTGGCGGTAGAGCTCCTCAAGAGGGAAGTGGACCTGCCCACCTTATTTCAGAAGGACCCCGTGTG
 EquCab_1A6a   (691) --GACCTCACATGGAATGTTCTCAAGAGAGATGTGCACTTACCCAGCTTATATCGGAAGGTCTCCATTTG
 EquCab_1A6b   (691) --GACCTTGCATGGAATCTTCTCAAGAAAGATGTGCACTTACCTGCCTTATATCAGAAGGGCTCCATTTG
 EquCab_1A6c   (691) --GAACTTGCATCTCATATCCTCAAGAGAGATGTGCACTTACCCGCCTTATATCAGAAGGGCTCTATTTG
   MusNig_1A6   (679) --GACCTCGCCTCCAACATCCTCAAGAGGGATGTGCACCTACCCACCTTGTATCAGAAGGGCTCCATCTG
   MusPut_1A6   (679) --GACCTCGCCTCCAACATCCTCAAGAGGGATGTGCACCTACCCACCTTGTATCAGAAGGGCTCCATCTG
   MirAng_1A6   (680) --GACCTCCCCTCCAATATCCTCAAGAGAGATGTGCACGTACCCACCTTGTATCGGAAGGGCTCCGTTTG
   PhoVit_1A6   (679) --GACCTCACCTCCAATATCCTCAAGAGAGATGTGCACTTACCCACCTTGTATCGGAAGGGCTCCATTTG
   UrsMar_1A6   (579) --GACCTTGCCTCCAACATCCTCAAGAGAGATGTGCACTTACCCACCTTGTATCGGAAGGGCTCCATTTG
   UrsThi_1A6   (578) --GACCTTGCCTCCAACATCCTCAAGAGAGATGTGCACTTACCCACCTTGTATCGGAAGGGCTCCATTTG
   ProLot_1A6   (535) --GACCTCGCCTCCCGTATCCTCAAGAGAGATGTGCACTTACCCACCTTGTATCGGAAGGGCTCCATTTG
   CanFam_1A6   (679) --GACCTTGCATCCAATATCCTCAAGAGAGATGTGCACTTACCCACCTTGTATCGGAACGGCTCCATTTG
   CanRuf_1A6   (578) --GACCTTGCATCCAATATCCTCAAGAGAGATGTGCACTTACCCACCTTGTATCGGAACGGCTCCATTTG
   ChrBra_1A6   (578) --GACCTTGCATCCAATATCCTCAAGAGAGATGTGCACTTACCCACCTTGTATTGGAACGGCTCCATTTG
   VulVul_1A6   (580) --GACCTTGCATCCAATATCCTCAAGAGAGATGTGCACTTACCCACCTTGTATCGGAACGGCTCCATTTG
   AilFul_1A6   (519) --GACCTCGCCTCCAATATCCTCAAGAGAGATGTGCACTTACCCACCTTGTATCGAAAGGGCTCCGTCTG
   ArcBin_1A6   (691) --GACCTCGCATCCAATATGCTCAAGAGAGATGTGCACTTGCCCACTTTGTATCGGAAGGGCTCCCTTTG
   CivCiv_1A6   (501) --GACCTCGCATCCGATATCCTCAAGAGAGATGTGTACTTACCCGCCTTGTATCGGAAGGGCCCCCTTTG
   HerJav_1A6   (691) --GACCTCGCATCCAGCATCCTCAAGAGAGGTGTGGACTTACCCACCTTGTATCGGAAGGGCTCCATTTG
   GalGal_1A6   (700) --GAACTTGCATATGAGATTTTGCAAAAGAAAGTGACAGCTACAGACCTTCTAAGCCATGGATCTGTCTG

                      771                                                                  840
   HomSap_1A6   (759) GCTGTTA-AGATATGACTTTGTGCTTGAATATCCTAGGCCGGTCATGCCCAACATGGTCTTCATTGGAGG
   PanTro_1A6   (759) GCTGTTA-AGATATGACTTTGTGCTTGAATATCCTAGGCCGGTCATGCCCAACATGGTCTTCATTGGAGG
   PonAbe_1A6   (759) GCTGTTA-AGATATGACTTTGTACTTGAGTATCCTAGGCCAGTCATGCCCAACATGGTCTTCATTGGAGG
   PapAnu_1A6   (759) GCTGTTA-AGATATGACTTTGTACTTGAGTATCCTAGGCCAGTCATGCCCAACATGGTCTTCATTGGAGG
   MacFas_1A6   (759) GCTGTTA-AGATATGACTTTGTACTTGAGTATCCTAGGCCAGTCATGCCCAACATGGTCTTCATTGGAGG
   AciJub_1A6   (748) GCTGTTA-CGATACGACTTTGTGTTCGAGTATCTCAGACCAGTCATGCCCAACATGGTTTTTATTGGAGG
   PanTig_1A6   (647) GCTGTTA-CGATACGACTTTGTGTTCGAGTATCCCAGACCAGTCATGCCCAACATGGTTTTCATTGGAGG
   PanOnc_1A6   (648) GCTGTTAACGATACGACTTTGTGTTCGAGTATCCCAGACCAGTCATGCCCAACATGGTTTTCATTGGAGG M9: bp768-769
   PanUnc_1A6   (648) GCTGTTA-TGATATGACTTTGTGTTCGAGTATCCCAGACCAGTCATGCCCAACATGGTTTTCATTGGAGG
   PanLeo_1A6   (648) GCTGTTA-CGATACGACTTTGTGTTCGAGTATCCCAGACCAGTCATGCCCAACATGGTTTTCATTGGAGG
   PanPar_1A6   (648) GCTGTTA-CGATACGACTTTGTGTTCGAGTATCCCAGACCAGTCATGCCCAACATGGTTTTCATTGGAGG
   LeoGeo_1A6   (748) GCTGTTA-CGATACGACTTTGTGTTCAAGTATCCCAGACCAGTCATGCCCAACATGGTTTTCATTGGAGG
   LeoTig_1A6   (748) GCTGTTA-CGATACGACTTTGTGTTCAAGTATCCCAGACCAGTCATGCCCAACATGGTTTTCATTGGAGG
   LeoWie_1A6   (748) GCTGTTA-CGATACGACTTTGTGTTCAAGTATCCCAGACCAGTCATGCCCAACATGGTTTTCATTGGAGG
   LynCan_1A6   (748) GCTGTTA-CGATACGACTTTGTGTTCGAGTATCCCAGACCAGTCATGCCCAACATGGTTTTCATTGGAGG
   LynRuf_1A6   (748) GCTGTTA-CGATACGACTTTGTGTTCGAGTATCCCAGACCAGTCATGCCCAACAGGGTTTTCATTGGAGG
```

```
 ParTem_1A6  (748) GCTGTTA-CGATACGACTTGGTGTTCGAGTATCCCAGACCAGTCATGCCCAACATGGTTTTCATTGGAGG
 PumCon_1A6  (744) GCCGTTA-CGATACGACTTTGTGTTCGAGTATCCCAGACCAGTCATGCCCAACATGGTTTTCATTGGAGG
 PumCo2_1A6  (744) GCTGTTA-CGATACGACTTTGTGTTCGAGTATCCCAGACCAGTCATGCCCAACATGGTTTTCATTGGAGG
 FelCat_1A6  (748) GCTGTTA-CAATACGACTTTGTGTTCGAGTATCCCAGACCAGTCATGCCCAACATGGTTTTCAT-GGAGG M5: bp827
 PriBen_1A6  (748) GCTGTTA-CAATACGACTTTGTGTTCGAGTATCCCAGACCAGTCATGCCCAACATGGTTTTCAT-GGAGG
 CarAur_1A6  (748) GCTGTTA-CGATACGACTTTGTGTTCGAGTATCCCAGACCAGTCATGCCCAACATGGTTTTCATTGGAGG
 CarSer_1A6  (750) GCTGTTA-CGATACGACTTTGTGTTCGAGTACCCCAGACCAGTCATGCCCAACATGGTTTTCATTGGAGG
 CroCro_1A6  (759) GCTGTTA-AGATATGACTTTGTGTTCGAGTATCCCAGACCAGTCATGCCCAACATGGTTTTCATTGGAGG
 ParBru_1A6  (759) GCTGTTA-AGATACGACTTTGTGTTCGAGTATCCCGGACCAGTCATGCCCAACATGGTTTTCATTGGAGG
 ProCri_1A6  (568) GCTGTTG-AGATACGACTTTGTGTTCGAGTATCCCAGACCGGTCATGCCCAACATGGT-TTCATCGGAGA
 HyaHya_1A6  (759) GCTGTTA-AGATACGACTTTGTGTTCGAGTATCCCAGACCAGTCATGCCCAACATGGTTTTCATTGGAGG
 BosTau_1A6  (750) GCTGTTA-AGATACGACTTTGTGTTCGAGTATCCCAGACCAGTCATGCCCAACACGGTCCTCATCGGAGG
 OviAri_1A6  (756) GCTGCTA-AGATACGACTTTGTGTTCGAGTATCCCAGACCAGTGATGCCCAACATGGTCTTCATCGGAGG
 SusScr_1A6  (759) GCTGATG-AGATACGACTTTGTCTTTGAGTTTCCCCGACCTGTCATGCCCAACATGGTCTTCATTGGAGG
MusMus_1A6a  (756) GCTGTTA-CGGTACGATTTTGTGTTCGAATATCCCAGGCCGGTCATGCCCAACATGATCTTCCTAGGAGG
MusMus_1A6b  (756) GCTGTTA-CGGTACGATTTTGTGTTCGAATATCCCAGGCCGGTCATGCCCAACATGATCTTCCTTGGAGG
 RatNor_1A6  (753) GCTGTTA-CGGTATGATTTTGTGTTCGAATACCCCCGGCCAGTCATGCCCAACATGATCTTCATTGGAGG
 OryCun_1A6  (759) GCTGCTA-AGATACGACTTTGTGTTTGAGTACCCCAGGCCGGTGATGCCCAACATGGTGCTCATTGGCGG
EquCab_1A6a  (759) GCTGTTA-AGATACGACTTTGTGTTTGAGTATCCAAGACCAGTCATGCCCAACATGGTCTTCATTGGAGG
EquCab_1A6b  (759) GCTGTTA-AGATATGACTTTGTGTTTGAGTATCCAAAACCAGTGATGCCCAACATGGTCTTCATTGGCGG
EquCab_1A6c  (759) GTTGTTA-AGATACGACTTTGCGTTTGAGTATCCAAGACCAGTCATGCCCAGCATGGTCTTCATTGGAGG
 MusNig_1A6  (747) GCTCTTA-AGATATGACTTTGTGTTTGAATATCCCAGACCGGTCATGCCTAACATGGTTTTCGTTGGAGG
 MusPut_1A6  (747) GCTCTTA-AGATATGACTTTGTGTTTGAATATCCCAGACCCCGTCTGCCTAACATGGTTTTCGTTGGAGG
 MirAng_1A6  (748) GCTCTTA---------------------------------------------------------------
 PhoVit_1A6  (747) GCTCTTA-CAATACGATTT----------------------------------------------------
 UrsMar_1A6  (647) GCTCTTA-CAATACGACTT----------------------------------------------------
 UrsThi_1A6  (646) GCTCTTA-CAATACGACTTT---------------------------------------------------
 ProLot_1A6  (603) GCTCTTA-CAATACAACTTTGTGCTCAAAA----------------------------------------
 CanFam_1A6  (747) GCTGTTA-AGATATGACTTTGTGTTTGAGTATCCCAGACCAGTCATGCCCAACATGGTTTTCATTGGAGG
 CanRuf_1A6  (646) GCTCTTA-CAATACGACTTT---------------------------------------------------
 ChrBra_1A6  (646) GCTCTTA-CAATACGA------------------------------------------------------
 VulVul_1A6  (648) GCTCTTA-CAATACGACTTT---------------------------------------------------
 AilFul_1A6  (587) GTTGCTA-AGATACGACTTTGTGTTTGAGTATCCCAGACCGGTCATGCCCAACATGGTTTTCATTGGAGG
 ArcBin_1A6  (759) GCTCTTA-CAATACGACTTTGTGTTCGAGTATCCCAGACCAGTCATGCCCAACATGGTCTTCATTGGAGG
 CivCiv_1A6  (569) GCTGTTA-AGATATGACTTTGTGCTCGAGTATCCCAGACCAGTCATGCCCAACATGGTTTTCATTGGAGG
 HerJav_1A6  (759) GCTGTTA-AGATATGACTTTGTGTTCGAGTATCCCAGACCAGTCATGCCCAACATGGTTTTC--------
 GalGal_1A6  (768) GCTCATG-AGATATGATTTTGTGTTTGAATTCCCAAGACCAACAATGCCAAACATGGTTTTTATTGGAGG

                   841                              874
 HomSap_1A6  (828) TATCAACTGTAAGAAGAGGAAAGACTTGTCTCAG
 PanTro_1A6  (828) TATCAACTGTAAGAAGAGGAAAGACTTGTCTCAC
 PonAbe_1A6  (828) TACCAACTGTAAGAAGAGGAAAGACTTGTCTCAG
 PapAnu_1A6  (828) TACCAACTGTAAGAAGAGGAAAGACCTGTCTGAG
 MacFas_1A6  (828) TACCAACTGTAAGAAGAGGAAAGACCTGTCTGAG
 AciJub_1A6  (817) GACCAACTGCAAGGAAAAGGGAGTCCTGTCTCAG
 PanTig_1A6  (716) GACCAATTGCAAGGAAAAGGGAGTCCTGTCTCAG
 PanOnc_1A6  (718) GACCAATTGCAAGGAAAAGGGAGTCCTGTCTCAG
 PanUnc_1A6  (717) GATGAATTGCAAGGAAAAGGGAGTCCTGTCTCAG
 PanLeo_1A6  (717) GACCAATTGCAAGGAAAAGGGAGTCCTGTCTCAG
 PanPar_1A6  (717) GACCAATTGCAAGGAAAAGGGAGTCCTGTCTCAG
 LeoGeo_1A6  (817) GACCAACTGCAAGGAAAAGGGAGTCCTGTCTCAG
 LeoTig_1A6  (817) GACCAACTGCAAGGAAAAGGGAGTCCTGTCTCAG
 LeoWie_1A6  (817) GACCAACTGCAAGGAAAAGGGAGTCCTGTCTCAG
 LynCan_1A6  (817) GACCAACTGCAAGGAAAAGGGAGTCCTGTCTCAG
 LynRuf_1A6  (817) GACCAACTACAAGGAAAAGGGAGTCCTGTCTCAG
 ParTem_1A6  (817) GGCCAACTGCAAGGAAAAGGGAGTCCTGTCTCAG
 PumCon_1A6  (813) GACCAACTGCAAGGAAAAGGGAGTCACGTCTCAG
 PumCo2_1A6  (813) GACCAACTGCAAGGAAAAGGGAGTCCTGTCTCAG
 FelCat_1A6  (816) GACCAACCTCAAGGAAAAGGGAGTCCTGTCTCAG
 PriBen_1A6  (816) GACCAACCTCAAGGAAAAGGGAGTCCTGTCTCAG
 CarAur_1A6  (817) GACCAACTGCAAGGAAAAGGGAGTCCTGTCTCAG
 CarSer_1A6  (819) GACCAACTGCAAGGAAAAGGGAGTCCTGTCTCAG
 CroCro_1A6  (828) GACCAACTGCAAGGAAAAGGGAGTCCTGTCTCAG
 ParBru_1A6  (828) GACCAACTGCAAGAAAAAGGGAGTCCTGTCTCAG
 ProCri_1A6  (636) CCA-------------------------------
 HyaHya_1A6  (828) GACCAACTGCAAGAAAAAGGGAGTC---------
 BosTau_1A6  (819) GTCCAGCTGCAAGAAACAGGGCGTCCTGTCTCAG
 OviAri_1A6  (825) GTCAGCTGCCAAGAAACAAGGCATCCTGCCTCGG
 SusScr_1A6  (828) GACCAACTGCAAGAAAGTGGGCATCCTGTCTCAG
MusMus_1A6a  (825) GATCAACTGTAAGAAGAAGGGAAAGCTGACTCAG
MusMus_1A6b  (825) GATCAACTGTAAGAAGAAGGGAAAGCTGACTCAG
 RatNor_1A6  (822) GACCAACTGCAAGAAGAAGGGGAACCTGTCTCAG
 OryCun_1A6  (828) GATCAACTGCAAGAAGCCGGACGTCCTGTCTCAG
EquCab_1A6a  (828) GGTCAACTGCAAGAAGGAGGGAACCCTGCCTCAG
EquCab_1A6b  (828) GGTCAACTGCAGGAAGGAGGGCATCCTGCCTCAG
EquCab_1A6c  (828) GGTCAACTGCAAGAAGGATAGAATCCTATCTCAG
 MusNig_1A6  (816) GAGCAATTGCAAGAAGACGGGGGTCCTGTCTCAG
 MusPut_1A6  (816) GAGCAATTGCAAGAAGATGGGGGTCCTGTCTCAG
```

```
MirAng_1A6  (755) ----------------------------------
PhoVit_1A6  (765) ----------------------------------
UrsMar_1A6  (665) ----------------------------------
UrsThi_1A6  (665) ----------------------------------
ProLot_1A6  (632) ----------------------------------
CanFam_1A6  (816) GACCAACTGCAAGATGAAGGGAGTCCTGCCTCAG
CanRuf_1A6  (665) ----------------------------------
ChrBra_1A6  (661) ----------------------------------
VulVul_1A6  (667) ----------------------------------
AilFul_1A6  (656) GACCAACTGCAAGAAGAAGGGAGTCCTGTCT---
ArcBin_1A6  (828) GACCAACTGCAAGGAAAAGGGAGTCCTG------
CivCiv_1A6  (638) GACCAACTGCAAGAAAAAGGGAGTCCTGTCTCAG
HerJav_1A6  (820) ----------------------------------
GalGal_1A6  (837) GATAAACTGTGCTCAGAAGAACAAGCTGTCTCAG
```
