# Supplementary material for: Evolution of a Major Drug Metabolizing Enzyme Defect in the Domestic Cat and Other Felidae: Phylogenetic Timing and the Role of Hypercarnivory
Source: PLoS One. 2011 Mar 28;6(3):e18046. doi: 10.1371/journal.pone.0018046 (PMC3065456; doi:10.1371/journal.pone.0018046)

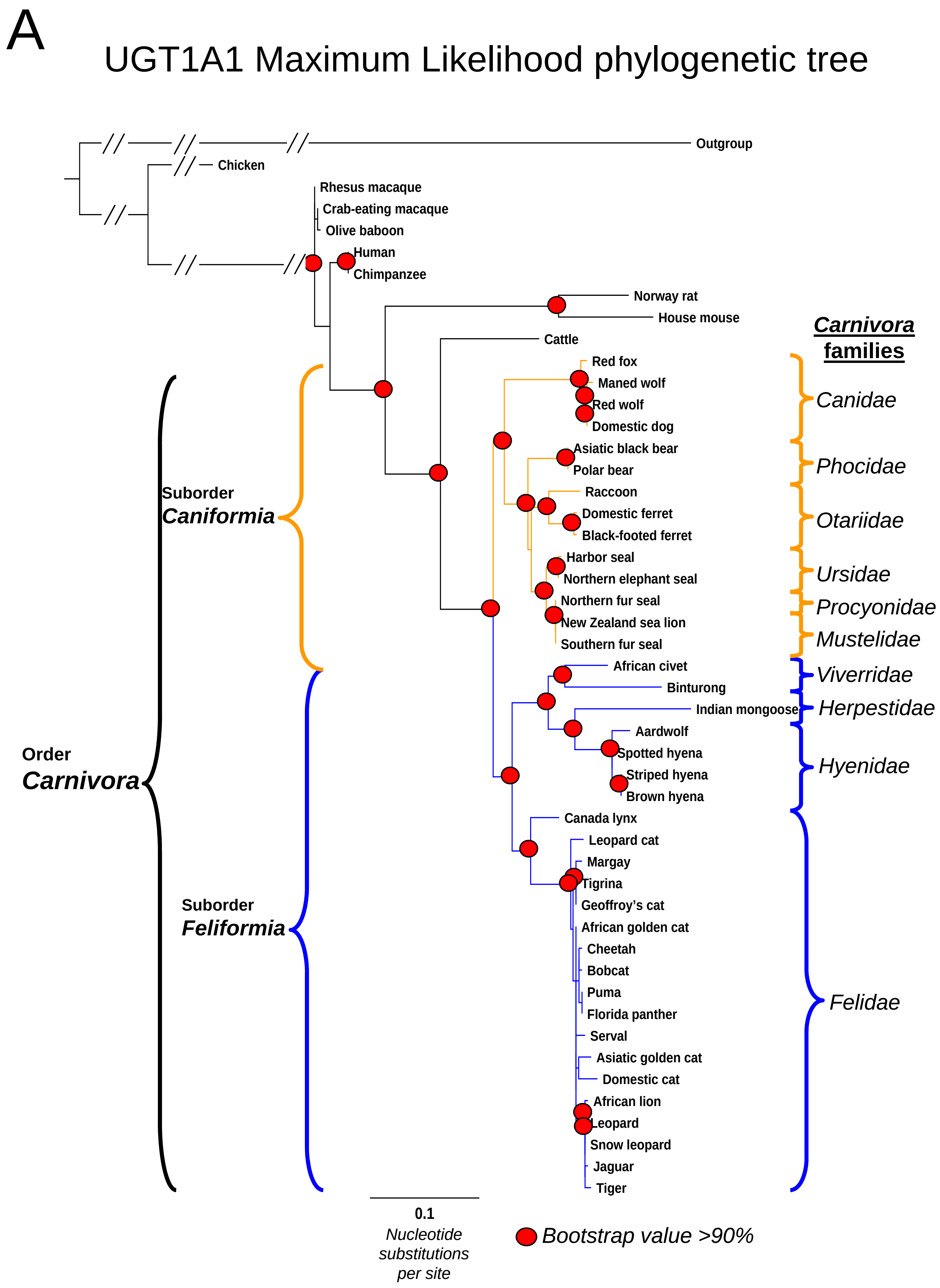
A
UGT1A1 Maximum Likelihood phylogenetic tree
Outgroup
Chicken
Rhesus macaque
Crab-eating macaque
Olive baboon
Human
Chimpanzee
Norway rat
House mouse
Cattle
Red fox
Maned wolf
Red wolf
Domestic dog
Asiatic black bear
Polar bear
Raccoon
Domestic ferret
Black-footed ferret
Harbor seal
Northern elephant seal
Northern fur seal
New Zealand sea lion
Southern fur seal
African civet
Binturong
Indian mongoose
Aardwolf
Spotted hyena
Striped hyena
Brown hyena
Canada lynx
Leopard cat
Margay
Tigrina
Geoffroy's cat
African golden cat
Cheetah
Bobcat
Puma
Florida panther
Serval
Asiatic golden cat
Domestic cat
African lion
Leopard
Snow leopard
Jaguar
Tiger
Carnivora families
Canidae
Phocidae
Otariidae
Ursidae
Procyonidae
Mustelidae
Viverridae
Herpestidae
Hyenidae
Felidae
Suborder Caniformia
Order Carnivora
Suborder Feliformia
0.1
Nucleotide substitutions per site
Bootstrap value >90%

B

UGT1A6 Maximum Likelihood phylogenetic tree

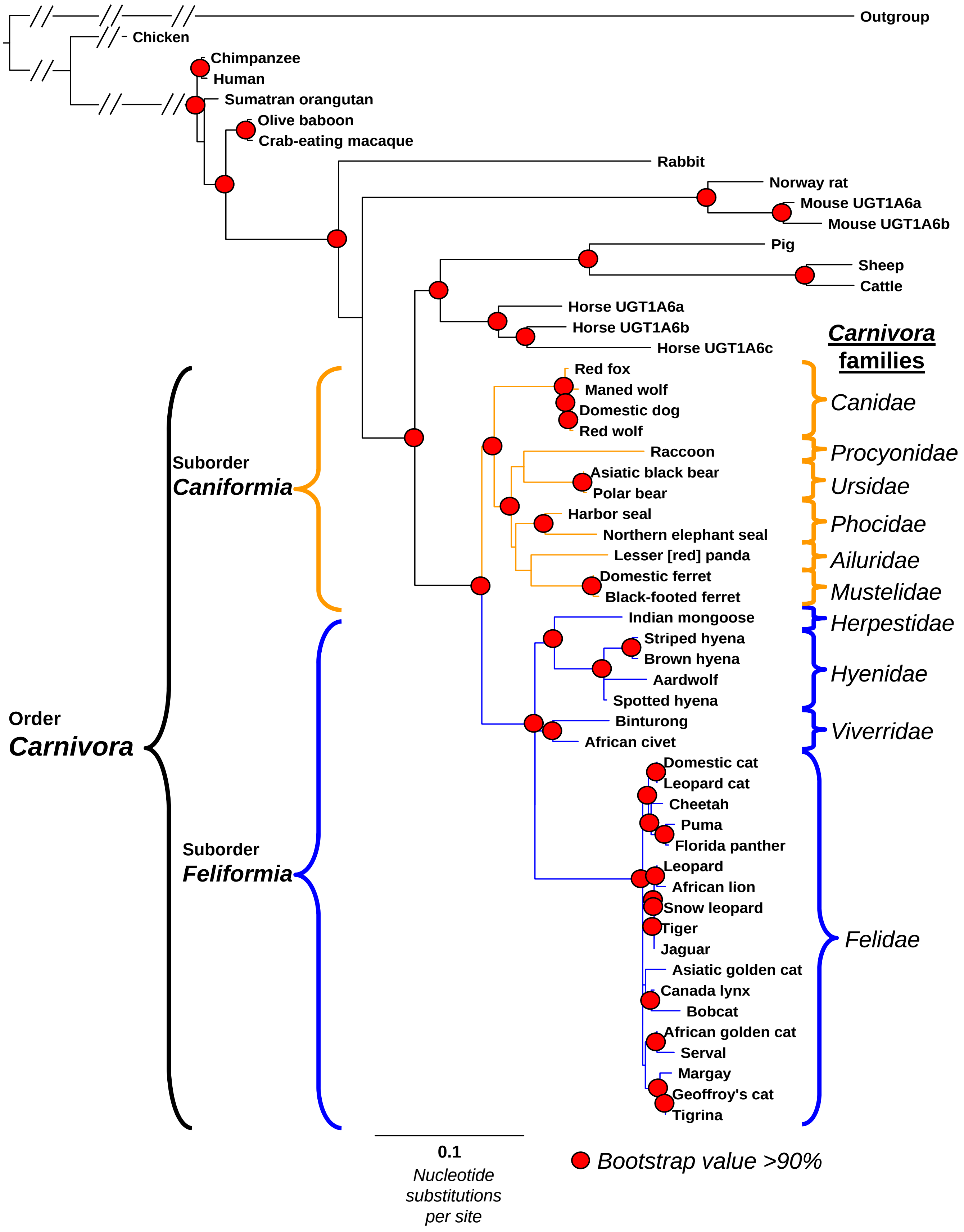

C

UGT1A1 Bayesian phylogenetic tree

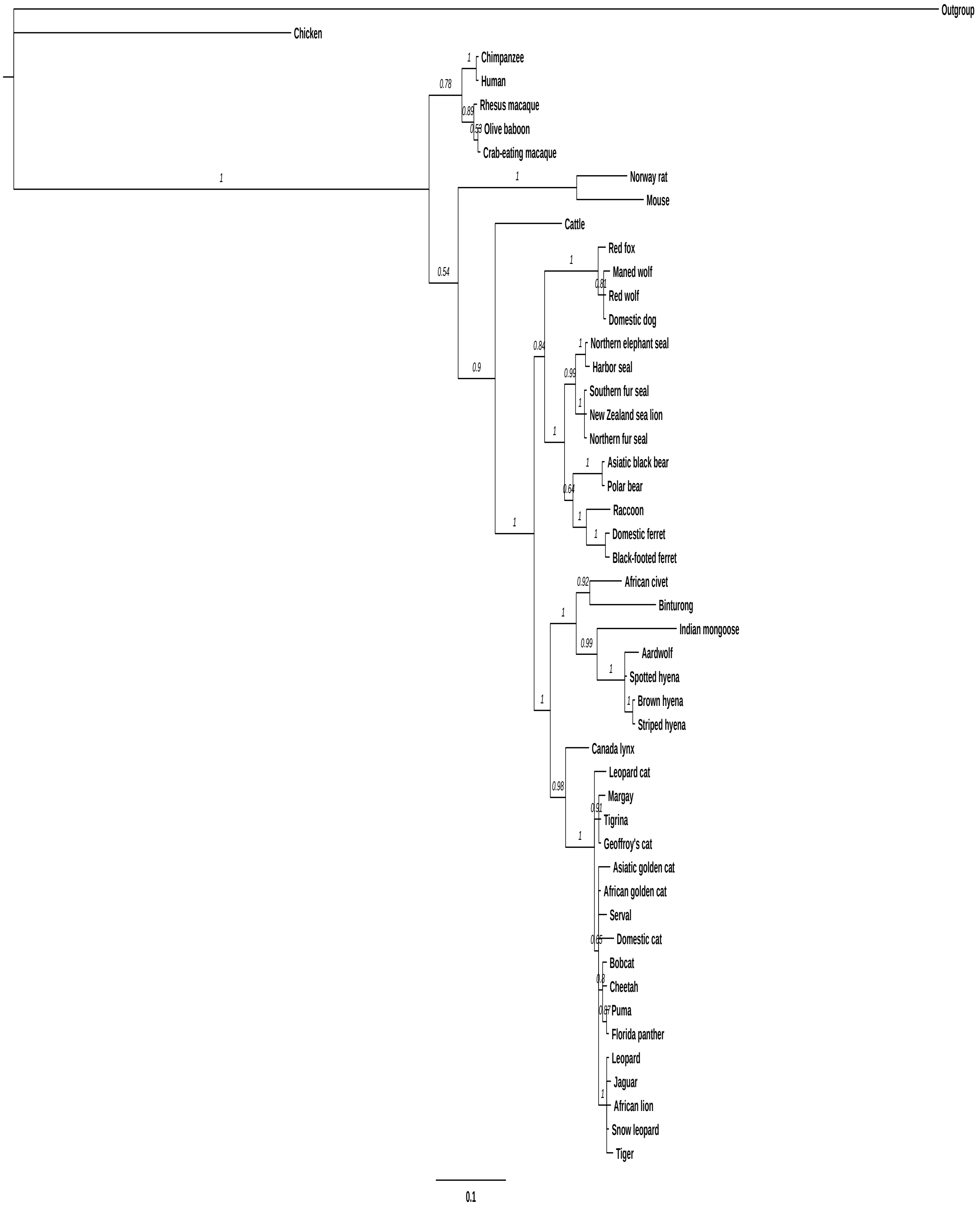

D

UGT1A6 Bayesian phylogenetic tree

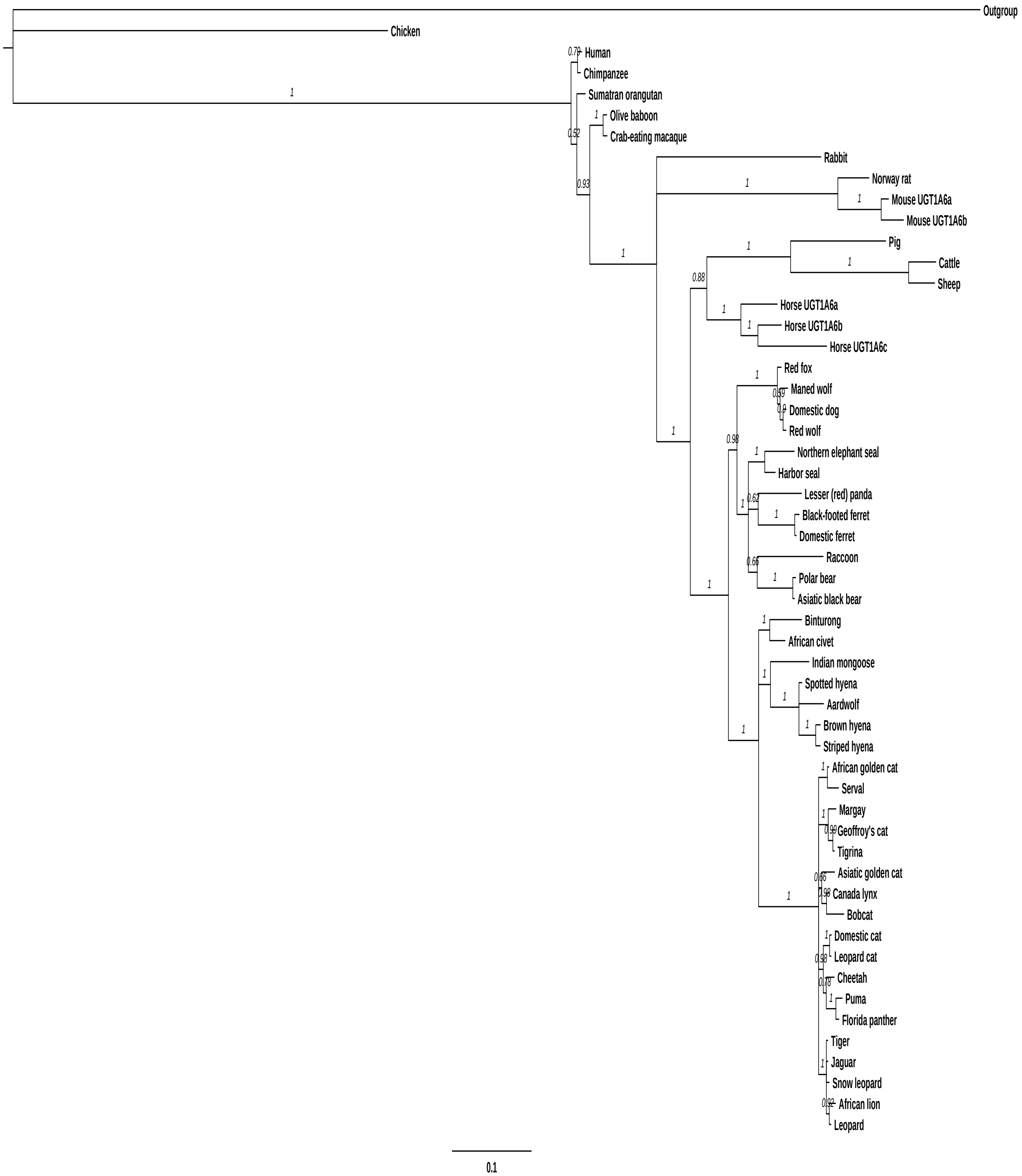

E

UGT1A1 Maximum Parsimony phylogenetic tree

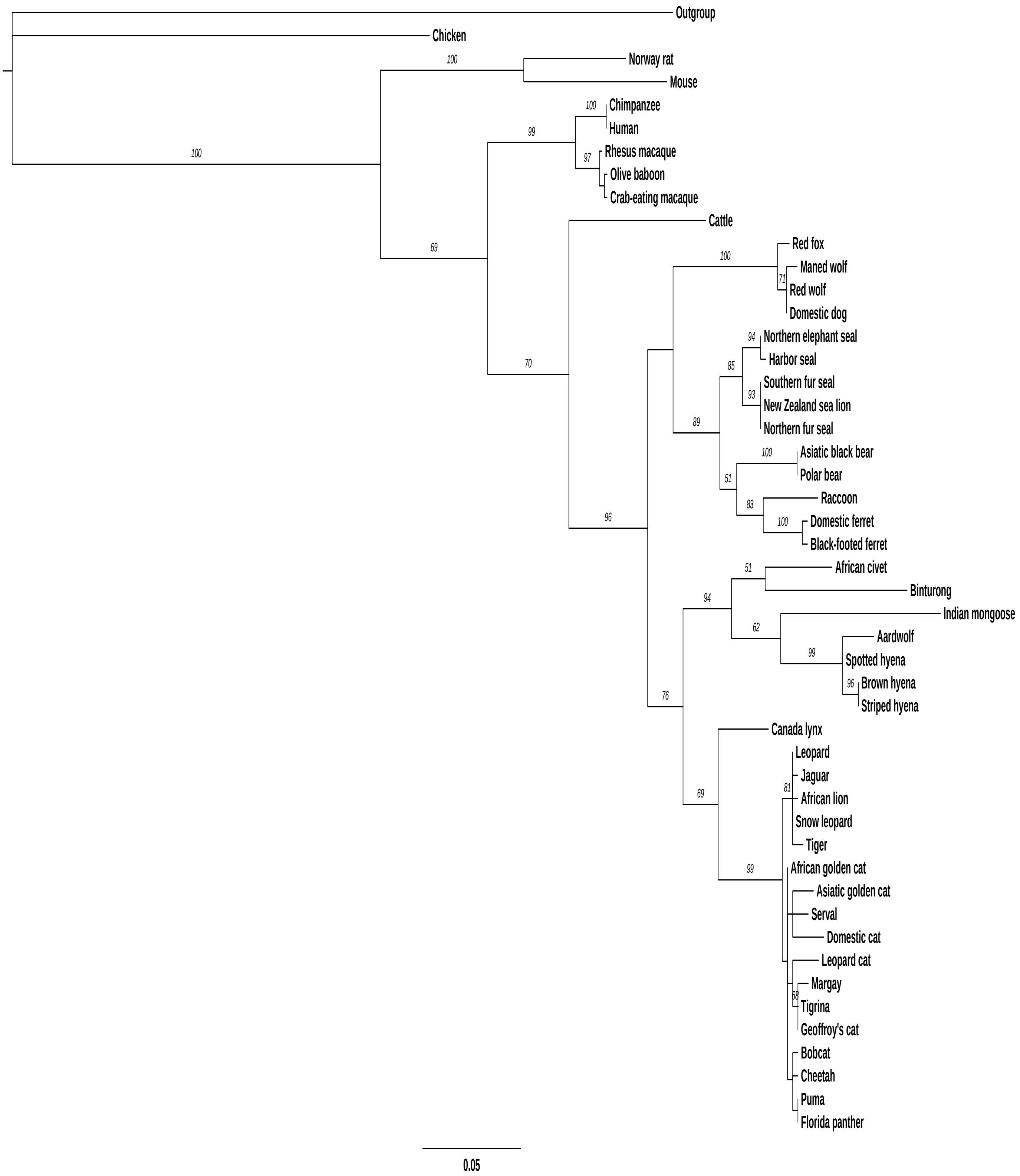

F

UGT1A6 Maximum Parsimony phylogenetic tree

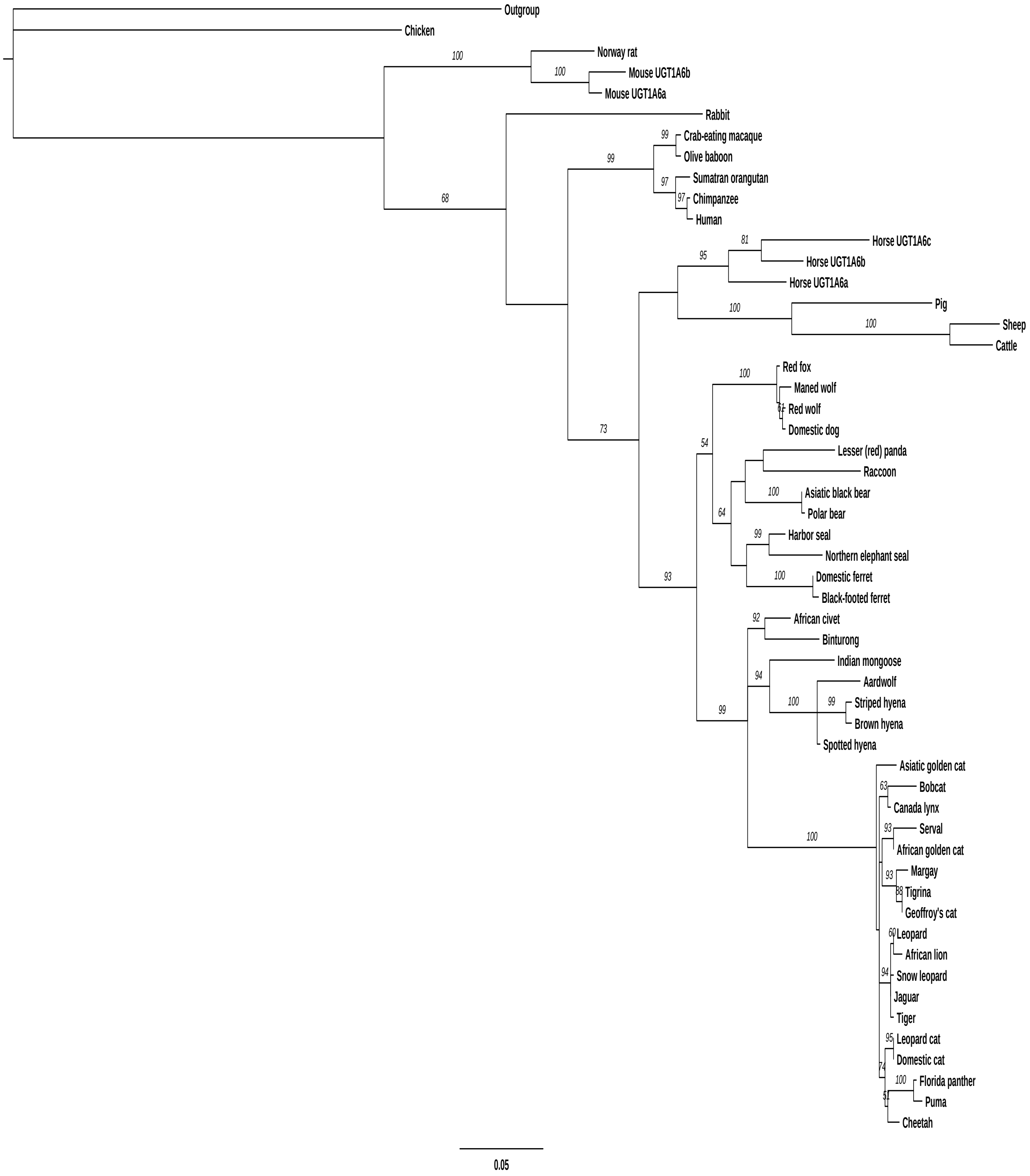

Supplement: Figure S3 — Phylogenetic trees constructed for UGT1A1 and UGT1A6 exon 1 sequences using three different inference methods. A. UGT1A1 maximum likelihood tree (RAxML, Ver. 7.0). B. UGT1A6 maximum likelihood tree (RAxML, Ver. 7.0). C. UGT1A1 Bayesian tree (MrBayes, Ver. 3.1) D. UGT1A6 Bayesian tree (MrBayes, Ver. 3.1) E. UGT1A1 maximum parsimony tree (PHYLIP, Ver. 3.6) F. UGT1A6 maximum parsimony tree (PHYLIP, Ver. 3.6). Bootstrap resampling confidence values as percentages (ML and MP trees) or posterior probabilities as ratios (Bayesian trees) are shown for each node. (PDF) [file pone.0018046.s003.pdf]
