# Supplementary material for: Evolution of a Major Drug Metabolizing Enzyme Defect in the Domestic Cat and Other Felidae: Phylogenetic Timing and the Role of Hypercarnivory
Source: PLoS One. 2011 Mar 28;6(3):e18046. doi: 10.1371/journal.pone.0018046 (PMC3065456; doi:10.1371/journal.pone.0018046)
Supplement: Table S1 — Genbank IDs of novel and existing UGT1A1 and UGT1A6 exon 1 sequences evaluated in this study. (PDF) [file pone.0018046.s004.pdf]

Table S1. Genbank IDs of novel and existing UGT1A1 and UGT1A6 exon 1 sequences evaluated in this study.

Code name	Scientific Name	Common Name	UGT1A1	Genbank	UGT1A6	Genbank
AciJub	*Acinonyx jubatus*	Cheetah	New	EF440364	New	EF440401
AilFul	*Ailurus fulgens*	Lesser (red) panda	-	-	New	EF440400
ArcBin	*Arctictis binturong*	Binturong	New	EF440362	New	EF440399
ArcFor	*Arctocephalus forsteri*	Southern fur seal	New	EF440363	-	-
BosTau	*Bos taurus*	Cattle	Genbank	NM_001105636	Genbank	NM_174762
CalUrs	*Callorhinus ursinus*	Northern fur seal	New	EF440369	-	-
CanFam	*Canis familiaris*	Domestic dog	Genbank	AC093453.40	Genbank	NM_1003078
CanRuf	*Canis rufus*	Red wolf	New	EF440368	New	EF440405
CarAur	*Caracal aurata*[6]	African golden cat	New	EF440370	New	EF440406
CarSer	*Caracal serval*[9]	Serval	New	EF440378	New	EF440414
ChrBra	*Chrysocyon brachyurus*	Maned wolf	New	EF440365	New	EF440402
CivCiv	*Civettictis civetta*	African civet	New	EF440366	New	EF440403
CroCro	*Crocuta crocuta*	Spotted hyena	New	EF440367	New	EF440404
EquCab	*Equus caballus*	Horse	-	-	Genbank	A:XM_001495878<br>B:XM_001495839<br>C:XM_001495919
FelCat	*Felis catus*	Domestic cat	Genbank	AF039137	Genbank	AF064085
GalGal	*Gallus gallus*	Chicken	Genbank	XM_001234327	Genbank	NW_001471728
HerJav	*Herpestes javanicus*	Indian mongoose	New	EF440383	New	EF440418
HomSap	*Homo sapiens*	Human	Genbank	M84125	Genbank	M84130
HyaHya	*Hyaena hyaena*	Striped hyena	New	EF440382	New	EF440417
LeoGeo	*Leopardus geoffroyi*[10]	Geoffroy's cat	New	EF440376	New	EF440412
LeoTig	*Leopardus tigrinus*[1]	Tigrina[2]	New	EF440380	New	EF440416
LeoWie	*Leopardus wiedii*[12]	Margay	New	EF440381	Genbank	AF177922
LynCan	*Lynx canadensis*	Canada lynx	New	EF440374	New	EF440410
LynRuf	*Lynx rufus*	Bobcat	New	EF440377	New	EF440413
MacFas	*Macaca fascicularis*	Crab-eating macaque	Genbank	AF104339	Genbank	AF104337
MacMul	*Macaca mulatta*	Rhesus macaque	Genbank	AF360121.1	-	-
MirAng	*Mirounga angustirostris*	Northern elephant seal	New	EF440384	New	EF440419
MusMus	*Mus musculus*	House mouse	Genbank	AF093878	Genbank	A:U09930<br>B:AY22720
MusNig	*Mustela nigripes*	Black-footed ferret	New	HM070401	New	HM070402
MusPut	*Mustela putorius furo*	Domestic ferret	New	EF440385	Genbank	AF333815
OryCun	*Oryctolagus cuniculus*	Rabbit	-	-	Genbank	U09030
OviAri	*Ovis aries*	Sheep	-	-	Genbank	AB018477
PanLeo	*Panthera leo*	African lion	New	EF440389	New	EF440422
PanOnc	*Panthera onca*	Jaguar	New	EF440391	New	EF440424
PanPar	*Panthera pardus*	Leopard	New	EF440392	New	EF440425
PanTig	*Panthera tigris*	Tiger	New	EF440393	New	EF440426
PanTro	*Pan troglodytes*	Chimpanzee	Genbank	DQ052657	Genbank	BC019861
PanUnc	*Panthera uncia*	Snow leopard	New	EF440394	New	EF440427
PapAnu	*Papio anubis*	Olive baboon	Genbank	NM_001112621	Genbank	NM_001112622
ParBru	*Parahyaena brunnea*	Brown hyena	New	EF440386	New	EF440420
ParTem	*Pardofelis temminckii*[7]	Asiatic golden cat[8]	New	EF440379	New	EF440415
PhoHoo	*Phocarctos hookeri*	New Zealand sea lion	New	EF440388	-	-
PhoVit	*Phoca vitulina*	Harbor seal	New	EF440395	New	EF440428
PonAbe	*Pongo abelii*	Sumatran orangutan	-	-	Genbank	NM_001132169
PriBen	*Prionailurus bengalensis*[11]	Leopard cat	New	EF440371	New	EF440407
ProCri	*Proteles cristatus*	Aardwolf	New	EF440387	New	EF440421
ProLot	*Procyon lotor*	Raccoon	New	EF440390	New	EF440423
PumCo2	*Puma concolor coryi*[5]	Florida panther	New	EF440372	New	EF440408
PumCon	*Puma concolor*[3]	Puma[4]	New	EF440373	New	EF440409
RatNor	*Rattus norvegicus*	Norway rat	Genbank	D38065	Genbank	D38061
SusScr	*Sus scrofa*	Pig	-	-	Genbank	XM_001927691
UrsMar	*Ursus maritimus*	Polar bear	New	EF440396	New	EF440429
UrsThi	*Ursus thibetanus*	Asiatic black bear	New	EF440397	New	EF440430
VulVul	*Vulpes vulpes*	Red fox	New	EF440398	New	EF440431

[1]Alternate scientific names: *Felis tigrinus, Felis tigrina, Oncifelis tigrinus.*
[2]Alternate common names: Little spotted cat, Oncilla, Cunaguaro, Tiger Cat.
[3]Alternate scientific name: *Felis concolor.*
[4]Alternate common names: Mountain lion, Catamount.
[5]Alternate scientific name: *Felis concolor coryi.*
[6]Alternate scientific name: *Felis aurata, Caracal aurata.*
[7]Alternate scientific name: *Catopuma temminckii.*
[8]Alternate common names: Asian Golden Cat and Temminck's Golden Cat
[9]Alternate scientific name: *Leptailurus serval*
[10]Alternate scientific name: Oncifelis geoffroyi
[11]Alternate scientific name: Felis bengalensis
[12]Alternate scientific name: Felis wiedii
