# Supplementary material for: Evolution of a Major Drug Metabolizing Enzyme Defect in the Domestic Cat and Other Felidae: Phylogenetic Timing and the Role of Hypercarnivory
Source: PLoS One. 2011 Mar 28;6(3):e18046. doi: 10.1371/journal.pone.0018046 (PMC3065456; doi:10.1371/journal.pone.0018046)
Supplement: Table S2 — Classification of species based on observed dietary behavior or inferred from the literature. (PDF) [file pone.0018046.s005.pdf]

**Table S2.** Classification of species based on observed dietary behavior or inferred from the literature.

| Scientific name | Common name | Classification according to Van Valkenburgh[1] | Classification used in this study | Basis for classification | References |
|---|---|---|---|---|---|
| **Felidae** | | | | | |
| *Acinonyx jubatus* | Cheetah | >70% meat group | Hypercarnivore | Inferred | 6 |
| *Caracal aurata* | African golden cat | Not studied | Hypercarnivore | Inferred | 3 |
| *Caracal serval* | Serval | >70% meat group | Hypercarnivore | Inferred | 6 |
| *Felis bengalensis* | Leopard cat | Not studied | Hypercarnivore | Inferred | 3, 7 |
| *Felis canadensis* | Canada lynx | >70% meat group | Hypercarnivore | More than 84% of the food matter consumed is animal source | 3, 4 |
| *Felis catus* | Domestic cat | Not studied | Hypercarnivore | More than 90% of the food matter consumed is animal source | 2 |
| *Felis concolor* | Mountain lion | >70% meat group | Hypercarnivore | 96% of biomass consumed is animal source | 13 |
| *Felis concolor coryi* | Florida panther | >70% meat group | Hypercarnivore | Inferred | 13, 14 |
| *Felis geoffroyi* | Geoffroy's cat | >70% meat group | Hypercarnivore | Inferred | 9 |
| *Felis rufus* | Bobcat | >70% meat group | Hypercarnivore | Inferred | 5 |
| *Felis tigrinus* | Little spotted cat | Not studied | Hypercarnivore | Inferred | 10 |
| *Felis wiedii* | Margay | Not studied | Hypercarnivore | Inferred | 11, 12 |
| *Panthera leo* | African lion | >70% meat group | Hypercarnivore | Inferred | 6 |
| *Panthera onca* | Jaguar | >70% meat group | Hypercarnivore | 98% biomass consumed is animal source | 13 |
| *Panthera pardus* | Leopard | >70% meat group | Hypercarnivore | | 15 |
| *Panthera tigris* | Tiger | >70% meat group | Hypercarnivore | More than 93% of diet is big mammals | 15 |
| *Panthera uncia* | Snow leopard | >70% meat group | Hypercarnivore | Inferred | 3 |
| *Pardofelis temminckii* | Asiatic golden cat | Not studied | Hypercarnivore | Inferred | 3 |
| | | | | 96% biomass consumed is animal source | 16 |
| **Viverridae** | | | | | |
| *Arctictis binturong* | Binturong | <50% meat group | Hypocarnivore | Inferred | 6 |
| *Civettictis civetta* | African civet | 50-70% meat group | Mesocarnivore | Inferred | 17 |
| **Hyenidae** | | | | | |
| *Crocuta crocuta* | Spotted hyena | >70% meat and bone | Hypercarnivore | Inferred | 6, 18 |
| *Hyaena hyaena* | Striped hyena | >70% meat and bone | Hypercarnivore | Inferred | 3 |
| *Parahyaena brunnea* | Brown hyena | >70% meat and bone | Hypercarnivore | Inferred | 3, 19, 20, 37 |
| *Proteles cristatus* | Aardwolf | Not studied | Hypercarnivore | Inferred | 21 |
| **Herpestidae** | | | | | |
| *Herpestes javanicus* | Mongoose | Not studied | Hypercarnivore | Inferred | 22, 23 |
| **Mustelidae** | | | | | |
| *Mustela putorius furo* | Domestic ferret | Not studied | Hypercarnivore | Inferred | 24 |
| *Mustela nigra* | Black-footed ferret | Not studied | Hypercarnivore | Inferred | 8 |
| **Ailuridae** | | | | | |
| *Ailurus fulgens* | Lesser (red) panda | Not studied | Hypocarnivore | Inferred | 27 |
| **Procyonidae** | | | | | |
| *Procyon lotor* | Raccoon | <50% meat group | Hypocarnivore | Inferred | 25, 26 |
| **Ursidae** | | | | | |
| *Ursus maritimus* | Polar bear | 50-70% meat group | Mesocarnivore | Inferred | 30, 31 |
| *Ursus thibetanus* | Asiatic black bear | <50% meat group | Hypocarnivore | Inferred | 28, 29 |
| **Canidae** | | | | | |
| *Canis familiaris* | Domestic dog | Not studied | Mesocarnivore | | 38 |
| *Canis rufus* | Red wolf | Not studied | Mesocarnivore | Inferred | 3 |
| *Chrysocyon brachyurus* | Maned wolf | 50-70% meat group | Mesocarnivore | 45.4% of diet is animal matter | 32 |
| *Vulpes vulpes* | Red fox | 50-70% meat group | Mesocarnivore | Inferred | 33 |
| **Otariidae** | | | | | |
| *Arctocephalus forsteri* | Southern fur seal | Not studied | Hypercarnivore | Inferred | 3 |
| *Callorhinus ursinus* | Northern fur seal | Not studied | Hypercarnivore | Inferred | 34 |
| *Phocarctos hookeri* | New Zealand sea lion | Not studied | Hypercarnivore | Inferred | 35 |
| **Phocidae** | | | | | |
| *Mirounga angustirostris* | Northern elephant sea | Not studied | Hypercarnivore | Inferred | 36 |
| *Phoca vitulina* | Harbor seal | Not studied | Hypercarnivore | Inferred | 3 |

**References:**


1. Van Valkenburgh, B. (1989) in Carnivore behavior, ecology and evolution, ed. Gittleman, J. L. (Cornell University Press, New York), Vol. 1, pp. 410-436.
2. Biró, Z. S., Lanszki, J., Szemethy, L., Heltai, M. & Randi, E. (2005) Journal of Zoology 266, 187-196
3. Nowak, R. M. (2005) Walker's carnivores of the world. (The Johns Hopkins University Press, Baltimore, MD)
4. Weber, J. M. & Weissbrodt, M. (2005) Acta Theriologica 44, 333-336.
5. McLean, M. L., McCay, T. S. & Lovallo, M. J. (2005) American Midland Naturalist 153, 450-453
6. http://www.honoluluzoo.org/binturong.htm Last accessed 12/2010
7. Rajaratnam, R., Sunquist, M., Rajaratnam, L. & Ambu, L. (2007) Journal of Tropical Ecology 23, 209-217
8. http://en.wikipedia.org/wiki/Black-footed_Ferret Last accessed 12/2010
9. Manfredi, C., Lucherini, M., Canepuccia, A. D. & Casanave, E. B. (2004) Journal of Mammalogy 85, 1111-1115
10. http://animaldiversity.ummz.umich.edu/site/accounts/information/Leopardus_tigrinus.htm Last accessed 12/2010
11. www.bbc.co.uk/nature/wildfacts/factfiles/44.shtm Last accessed 12/2010
12. Wang, E. (2002) Studies on Neotropical Fauna and Environment 37, 207-212
13. Novack, A. J., Main, M. B., Sunquist, M. E. & Labisky, R. F. (2005) Journal of Zoology 267, 167-178
14. Maehr, D. S., Belden, R. C., Land, E. D. & Wilkins, L. (1990) The Journal of Wildlife Management 54, 420-423

15. Karanth, K. U. & Sunquist, M. E. (1995) Journal of Animal Ecology 64, 439-450
16. Henschel, P., Abernethy, K. A. & White, L. J. T. (2005) African Journal of Ecology 43, 21-28
17. http://animaldiversity.ummz.umich.edu/site/accounts/information/Civettictis_civetta.htm Last accessed 12/2010
18. Di Silvestre, I., Novelli, O. & Bogliani, G. (2000) African Journal of Ecology 38, 102-107
19. http://animaldiversity.ummz.umich.edu/site/accounts/information/Parahyaena brunnea.htm Last accessed 12/2010
20. Siegfried, W. R. (1984) South African Journal of Zoology 19, 61
21. Williams, J. B., Anderson, M. D. & Richardson, P. R. K. (1997) Ecology 78, 2588-2602
22. http://animaldiversity.ummz.umich.edu/site/accounts/information/Herpestes_javanicus.htm Last accessed 12/2010
23. Seaman, G. A. & Randall, J. E. (1962) Journal of Mammalogy, 544-546
24. http://www.oregonzoo.org/Cards/Ed_Program/ferret.htm Last accessed 12/2010
25. Schoonover, L. J. & Marshall, W. H. (1951) Journal of Mammalogy 32, 422-428
26. Smith, R. A., Kennedy, M. L. & Baumgardner, G. D. (1987) Journal of the Tennessee Academy of Science 62, 79-82
27. http://animaldiversity.ummz.umich.edu/site/accounts/information/Ailurus_fulgens.htm Last accessed 12/2010
28. Hwang, M. H., Garshelis, D. L. & Wang, Y. (2002) Ursus 13, 111-125.
29. Huygens, O. C., Miyashita, T., Carr, M., Izumiyama, S., Sugawara, T. & Hayashi, H. (2003) Ursus 14, 236-245
30. http://www.seaworld.org/animal-info/info-books/polar-bear/diet.htm Last accessed 12/2010
31. Derocher, A. E., Wiig, Ø. & Andersen, M. (2002) Polar Biology 25, 448-452
32. Juarez, K. M. & Marinho-Filho, J. (2002) Journal of Mammalogy 83, 925-933
33. Baltrunaite, L. (2002) Acta Zoologica Lituanica 12, 362-368
34. Perez, M. A. & Bigg, M. A. (1986) Fishery Bulletin 84, 957-971
35. Fea, N. I., Harcourt, R. G. & Lalas, C. (1999) Wildlife Research 26, 147-160.
36. Condit, R. & Le Boeuf, B. J. (1984) Journal of Mammalogy 65, 281-290
37. Mills, M. (1982) Mammalian Species 194, 1-5.
38. Vanak, A. & Gompper, M. E. (2009) Mammal Review 39, 265-283.
