# Supplementary material for: Evolution of a Major Drug Metabolizing Enzyme Defect in the Domestic Cat and Other Felidae: Phylogenetic Timing and the Role of Hypercarnivory
Source: PLoS One. 2011 Mar 28;6(3):e18046. doi: 10.1371/journal.pone.0018046 (PMC3065456; doi:10.1371/journal.pone.0018046)
Supplement: Table S3 — Protein content of commercial zoo animal diets formulated for various Carnivora in relation to the dietary classification proposed in this study. (PDF) [file pone.0018046.s006.pdf]

Table S3. Protein content of commercial zoo animal diets formulated for various Carnivora in relation to the dietary classification proposed in this study.

**Species**	**Crude Protein Content (% w/w)**[1]	**Dietary classification**
Ferret	38	Hypercarnivore
Small felines	36	Hypercarnivore
Large felines	35	Hypercarnivore
Polar bear	30.5	Mesocarnivore
Canine	28.5	Mesocarnivore
"Omnivores" including bears and raccoons	25	Hypocarnivore
Herbivores	14	Non-carnivora

[1] Data from Mazuri (PMI Nutrition International, St Louis, Mo, USA); https://www.mazuri.com/indexMazuri.asp (last accessed 12/28/2010) .
