# Supplementary material for: Evolution of a Major Drug Metabolizing Enzyme Defect in the Domestic Cat and Other Felidae: Phylogenetic Timing and the Role of Hypercarnivory
Source: PLoS One. 2011 Mar 28;6(3):e18046. doi: 10.1371/journal.pone.0018046 (PMC3065456; doi:10.1371/journal.pone.0018046)
Supplement: Table S4 — Origin of DNA samples used for sequencing in this study. (PDF) [file pone.0018046.s007.pdf]

Table S4. Origin of DNA samples used for sequencing in this study.

Code[1] name	Lab ID	Source population	Captive[2] location	Collector	Affiliation[2]	Sample[2] provided by
AciJub	CHE-1		White Oak CC	S Citino; C Fiorello	White Oak CC	White Oak CC
AciJub	CHE-2		White Oak CC	S Citino; C Fiorello	White Oak CC	White Oak CC
AciJub	CHE-3		White Oak CC	S Citino; C Fiorello	White Oak CC	White Oak CC
AciJub	CHE-4		White Oak CC	S Citino; C Fiorello	White Oak CC	White Oak CC
AciJub	CHE-5		RWP&Z	J Martin	RWP&Z	RWP&Z
AciJub	CHE-6		RWP&Z	J Martin	RWP&Z	RWP&Z
AilFul	AFU-16		NZP	M Bush	NZP	LGD NCI
AilFul	AFU-1B					LGD NCI
ArcBin	BIN-1		Southwick's Zoo	P Brewer	Southwick's Zoo	Southwick's Zoo
ArcFor	AFO-5	New Zealand		G Lento	Univ. of Aukland	LGD NCI
CalUrs	CUR-2	Alaska		LM Rotterman	LGD NCI	LGD NCI
CalUrs	CUR-3	Alaska		LM Rotterman	LGD NCI	LGD NCI
CanRuf	REW-1		RWP&Z	J Martin	RWP&Z	RWP&Z
CanRuf	REW-2		RWP&Z	J Martin	RWP&Z	RWP&Z
CanRuf	REW-3		RWP&Z	J Martin	RWP&Z	RWP&Z
CarAur	PAU-2B					LGD NCI
CarSer	SER-1		Private	M Court	Tufts Vet. School	Tufts Vet. School
ChrBra	MAW-1		White Oak CC	S Citino; C Fiorello	White Oak CC	White Oak CC
ChrBra	MAW-2		White Oak CC	S Citino; C Fiorello	White Oak CC	White Oak CC
CivCiv	VCI-1		Philadelphia Zoo	KC Hinshaw	Philadelphia Zoo	LGD NCI
CroCro	HYE-1		Private	M Court	Tufts Vet. School	Tufts Vet. School
CroCro	CCR-1		Henry Doorly Zoo	D Armstrong	Henry Doorly Zoo	LGD NCI
CroCro	CCR-21	Masi Mara, Kenya	UC Berkeley	LG Frank	FSBR UCB	LGD NCI
CroCro	CCR-22	Masi Mara, Kenya	UC Berkeley	LG Frank	FSBR UCB	LGD NCI
CroCro	CCR-23	Masi Mara, Kenya	UC Berkeley	LG Frank	FSBR UCB	LGD NCI
CroCro	CCR-28	Masi Mara, Kenya	UC Berkeley	LG Frank	FSBR UCB	LGD NCI
CroCro	CCR-34	Laikipia, Kenya		LG Frank	Laikipia PP	LGD NCI
CroCro	CCR-35	Laikipia, Kenya		LG Frank	Laikipia PP	LGD NCI
CroCro	CCR-78	Laikipia, Kenya		LG Frank	Laikipia PP	LGD NCI
CroCro	CCR-79	Laikipia, Kenya		LG Frank	Laikipia PP	LGD NCI
CroCro	CCR-80	Laikipia, Kenya		LG Frank	Laikipia PP	LGD NCI
CroCro	CCR-81	Laikipia, Kenya		LG Frank	Laikipia PP	LGD NCI
CroCro	CCR-236	Masi Mara, Kenya	UC Berkeley	LG Frank	FSBR UCB	LGD NCI
CroCro	CCR-274	Serengeti NP, Tanz.	UC Berkeley	ME Roelke	Messerli Found.	LGD NCI
HerJav		Hawaii		M Reed	Dept. Biol., Tufts Univ.	M Reed
HyaHya	HHY-8	Laikipia, Kenya		LG Frank	Laikipia PP	LGD NCI
HyaHya	HHY-13	Laikipia, Kenya		LG Frank	Laikipia PP	LGD NCI
HyaHya	HHY-16	Laikipia, Kenya		LG Frank	Laikipia PP	LGD NCI
HyaHya	HHY-17	Laikipia, Kenya		LG Frank	Laikipia PP	LGD NCI
LeoGeo	OGE-3		Blijdorp Zoo	M Bush	NZP	LGD NCI
LeoGeo	OGE-29		Cordoba Zoo	S Alcazar	Cordoba Zoo	LGD NCI
LeoGeo	OGE-70		NFREF	L Davidson	NFREF	LGD NCI
LeoTig	LTI-25		Sorocaba Zoo	A Nunes	Sorocaba Zoo	LGD NCI
LeoTig	LTI-27		Campinas Zoo	WE Johnson	LGD NCI	LGD NCI
LeoTig	LTI-37		Itaipu Zoo	WE Johnson	LGD NCI	LGD NCI
LeoWie	MAR-1		Tufts -Wildlife	G Kaufman	Tufts Vet. School	Tufts -Wildlife
LynCan	LYN-1		Tufts -Wildlife	G Kaufman	Tufts Vet. School	Tufts -Wildlife
LynRuf	BOB-1		Ecotarium	G Kaufman	Tufts Vet. School	Ecotarium

Code[1] name	Lab ID	Source population	Captive[2] location	Collector	Affiliation[2]	Sample[2] provided by
MirAng	Man#1	San Nicholas Island, CA		KP Koepfli	UCLA	EEB, UCLA
MirAng	Man#2	San Nicholas Island, CA		KP Koepfli	UCLA	EEB, UCLA
MirAng	Man#3	San Nicholas Island, CA		KP Koepfli	UCLA	EEB, UCLA
MirAng	MAN-14	Cape San Martin, CA		K Ralls	NZP	LGD NCI
MirAng	MAN-16	Cape San Martin, CA		K Ralls	NZP	LGD NCI
MusNig	BFF-1			S Wisely	Div. Biology, KSU	Div. Biology, KSU
MusNig	BFF-2			S Wisely	Div. Biology, KSU	Div. Biology, KSU
MusNig	BFF-3			S Wisely	Div. Biology, KSU	Div. Biology, KSU
MusNig	BFF-4			S Wisely	Div. Biology, KSU	Div. Biology, KSU
MusNig	BFF-5			S Wisely	Div. Biology, KSU	Div. Biology, KSU
MusNig	BFF-6			S Wisely	Div. Biology, KSU	Div. Biology, KSU
MusNig	BFF-7			S Wisely	Div. Biology, KSU	Div. Biology, KSU
MusNig	BFF-8			S Wisely	Div. Biology, KSU	Div. Biology, KSU
MusNig	BFF-9			S Wisely	Div. Biology, KSU	Div. Biology, KSU
MusNig	BFF-10			S Wisely	Div. Biology, KSU	Div. Biology, KSU
MusNig	BFF-11			S Wisely	Div. Biology, KSU	Div. Biology, KSU
MusNig	BFF-12			S Wisely	Div. Biology, KSU	Div. Biology, KSU
MusNig	BFF-13			S Wisely	Div. Biology, KSU	Div. Biology, KSU
MusNig	BFF-14			S Wisely	Div. Biology, KSU	Div. Biology, KSU
MusNig	BFF-15			S Wisely	Div. Biology, KSU	Div. Biology, KSU
MusNig	BFF-16			S Wisely	Div. Biology, KSU	Div. Biology, KSU
MusNig	BFF-17			S Wisely	Div. Biology, KSU	Div. Biology, KSU
MusNig	BFF-18			S Wisely	Div. Biology, KSU	Div. Biology, KSU
MusNig	BFF-19			S Wisely	Div. Biology, KSU	Div. Biology, KSU
MusNig	BFF-20			S Wisely	Div. Biology, KSU	Div. Biology, KSU
MusPut	FER-1		MIT DCM	R Marini	MIT DCM	MIT DCM
MusPut	FER-2		MIT DCM	R Marini	MIT DCM	MIT DCM
MusPut	FER-3		MIT DCM	R Marini	MIT DCM	MIT DCM
MusPut	FER-4		MIT DCM	R Marini	MIT DCM	MIT DCM
MusPut	FER-5		MIT DCM	R Marini	MIT DCM	MIT DCM
MusPut	FER-6		MIT DCM	R Marini	MIT DCM	MIT DCM
MusPut	FER-7		MIT DCM	R Marini	MIT DCM	MIT DCM
MusPut	FER-8		MIT DCM	R Marini	MIT DCM	MIT DCM
PanLeo	PLE-151	Kruger Park, South Africa		JG Howard	NZP	LGD NCI
PanLeo	PLE-154	Kruger Park, South Africa		JG Howard	NZP	LGD NCI
PanLeo	PLE-156	Kruger Park, South Africa		JG Howard	NZP	LGD NCI
PanOnc	PON-49	Chaco, Praguay	Itaipu Zoo	WE Johnson	LGD NCI	LGD NCI
PanOnc	PON-56	Falcon St, Venez.	Maracay Zoo	WE Johnson	LGD NCI	LGD NCI
PanOnc	PON-71			RN de Morais	DA Leme, Brasil	LGD NCI
PanPar	PPA-172	Laikipia, Kenya		LG Frank	Laikipia PP	LGD NCI
PanPar	PPA-173	Laikipia, Kenya		LG Frank	Laikipia PP	LGD NCI
PanPar	PPA-179	Laikipia, Kenya		LG Frank	Laikipia PP	LGD NCI
PanTig	TIG-1		White Oak CC	S Citino; C Fiorello	White Oak CC	White Oak CC
PanTig	TIG-2		White Oak CC	S Citino; C Fiorello	White Oak CC	White Oak CC
PanUnc	SNL-1		RWP&Z	J Martin	RWP&Z	RWP&Z
PanUnc	SNL-2		RWP&Z	J Martin	RWP&Z	RWP&Z
ParBru	HBR-1	Namibia		L Marker	CCF	LGD NCI
ParBru	HBR-2	Namibia		L Marker	CCF	LGD NCI
ParBru	Pbr#1		San Diego Zoo	KP Poepfli	UCLA	EEB, UCLA

Code[1] name	Lab ID	Source population	Captive[2] location	Collector	Affiliation[2]	Sample[2] provided by
ParBru	Pbr#2		San Diego Zoo	KP Poepfli	UCLA	EEB, UCLA
ParTem	PTE-8		EFBC	J Maynard	EFBC	LGD NCI
PhoHoo	PHO-2	New Zeland		G Lento	Univ. of Aukland	LGD NCI
PhoVit	PVG-1		San Diego Zoo	O Ryder	San Diego Zoo	LGD NCI
PhoVit	PVG-2	Prince Willian Sound, AK		LM Rotterman	LGD NCI	LGD NCI
PriBen	PBE-16		NIHAC	ME Roelke	LGD NCI	LGD NCI
PriBen	PBE-31	Northern Korea	Tallinn Zoo Park	ME Roelke	LGD NCI	LGD NCI
PriBen	PBE-72		Lotsaspots	G & L Kent	Lotsaspots	LGD NCI
ProCri	PCR-1		San Antonio Zoo	J Martenson	LGD NCI	LGD NCI
ProCri	PCR-2	Serengeti Nat. Park, Tanz.		ME Roelke	Messerli Found.	LGD NCI
ProLot	RAC-1	Massachusetts	Tufts -Wildlife	G Kaufman	Tufts Vet. School	Tufts -Wildlife
ProLot	RAC-2	Massachusetts	Tufts -Wildlife	G Kaufman	Tufts Vet. School	Tufts -Wildlife
PumCo2	FLP-1	Southern Florida	White Oak CC	S Citino; C Fiorello	White Oak CC	White Oak CC
PumCo2	FLP-2	Southern Florida	White Oak CC	S Citino; C Fiorello	White Oak CC	White Oak CC
PumCo2	FLP-3	Southern Florida	White Oak CC	S Citino; C Fiorello	White Oak CC	White Oak CC
PumCon	PUM-1		Ecotarium	G Kaufman	Tufts Vet. School	Ecotarium
UrsMar	POL-1		RWP&Z	J Martin	RWP&Z	RWP&Z
UrsMar	POL-2		RWP&Z	J Martin	RWP&Z	RWP&Z
UrsMar	POL-3		RWP&Z	J Martin	RWP&Z	RWP&Z
UrsThi	BEA-1		RWP&Z	J Martin	RWP&Z	RWP&Z
VulVul			Tufts -Wildlife	G Kaufman	Tufts Vet. School	Tufts -Wildlife

[1] Refer to Table S1 for scientific and common names

[2] Facility name abbreviations: Blijdorp Zoo, The Netherlands; Campinas Zoo, Brasil; Cordoba Zoological Park, Argentina; CCF - Cheetah Conservation Fund, Otjiwarongo, Namibia; Division of Biology, Kansas State University, Manhattan, KS; Ecotarium, Worcester, MA; EEB, UCLA - Department of Ecology and Evolutionary Biology, Univ. of Calif., Los Angeles, CA.; EFBC - Exotic Feline Breeding Compound Feline Conservation Center, CA; FSBR UCB - Field Station for Behavioral Research at Univ. of California Berkeley, CA; Itaipu Zoo, Paraguay; Lotsaspots, Stilwell, KS; LGD NCI - Laboratory of Genomic Diversity, National Cancer Institute, Frederick, MD; Laikipia Predator Project, Mpala Research Center, Kenya; Maracay Zoo, Venezuela; Messerli Foundation, Zurich, Switzerland; MIT DCM - Massachusetts Institute of Technology, Department of Comparative Medicine, MA; NZP - National Zoological Park, Washington DC; NIHAC - NIH Animal Center, MD; Northwood Felid Research & Education Foundation , OH; Philadelphia Zoolological Garden, PA; .; RWP&Z - Roger Williams Park and Zoo, RI; San Antonio Zoological Gardens and Aquarium, TX; Sorocaba Zoo, Brasil; Southwick's Zoo, Mendon, MA; Tallinn Zoo Park, Estonia; Tufts University Small Animal Hospital, North Grafton, MA; Tufts University Wildlife Clinic, North Grafton, MA; UC Berkeley - University of California Berkeley, CA; University of Auckland, New Zealand; White Oak CC - White Oak Plantation Conservation Center, Yulee, FL;
