# Supplementary material for: Evolution of a Major Drug Metabolizing Enzyme Defect in the Domestic Cat and Other Felidae: Phylogenetic Timing and the Role of Hypercarnivory
Source: PLoS One. 2011 Mar 28;6(3):e18046. doi: 10.1371/journal.pone.0018046 (PMC3065456; doi:10.1371/journal.pone.0018046)
Supplement: Table S5 — PCR primers that successfully amplified UGT1A1 and UGT1A6 exons 1. (PDF) [file pone.0018046.s008.pdf]

Table S5. PCR primers that successfully amplified UGT1A1 and UGT1A6 exons 1.

Common name	UGT1A1 primer pair[1]	UGT1A6 primer pair[1]
Canada lynx	490-491	447-448; 449-450
Bobcat	467-468 490-491	447-448; 449-450
Serval	467-468; 490-491	447-448
Asiatic golden cat	490-491	473-450
African golden cat	490-491	473-450; 473-448
Leopard cat	490-491	473-450
Geoffroy's cat	490-491	473-450
Tigrina	490-491	473-450
Margay	467-468; 490-491	447-448; 449-450;476-477
Puma	490-491	447-448; 449-450
Florida panther	490-491	447-448; 449-450
Tiger	467-468	449-450
Snow leopard	467-468; 490-491	476-477; 476-450; 476-475; 449-477
Leopard	490-491	449-450
Jaguar	490-491	449-450
African lion	490-491	476-450; 473-450
Cheetah	467-468; 490-491	447-448; 449-450
Domestic ferret	467-468	-[2]
Black-footed ferret	467-468	447-448
Binturong	467-468	447-448; 449-450; 473-474
African civet	490-491	476-474
Spotted hyena	490-491	473-450
Brown hyena	490-491	473-474; 476-474; 447-448
Aardwolf	490-491	476-474; 473-474
Mongoose	490-491	449-450
Raccoon	467-468	447-448; 473-448
Lesser red panda	-[3]	476-474
Asiatic black bear	467-468	447-448
Polar bear	467-468	447-448
Red wolf	467-468	447-448; 449-450
Maned wolf	467-468; 490-491	447-448
Red fox	467-468	447-448
Northern fur seal	490-491	-[3]
Southern fur seal	490-491	-[3]
New Zealand sea lion	490-491	-[3]
Northern elephant seal	490-491	473-450; 447-448; 449-636
Dog	467-468; 490-491	449-450
Harbor seals	490-491	447-448; 449-636
Striped hyena	490-491	449-450; 473-474; 447-448

[1] Primer sequences (5'-3'):

PRI 447 - TRGGKGACARGCTKCTGGTGGTYC
PRI 448 - TYRARCACAAARTCRTAYYKTAASAGCCA
PRI 449 - GGGCAAAATTCAGAGCCAGGAGAGGTAG
PRI 450 - CAAAGAGCCAAATGCACGAGGGA
PRI 467 - CCHRTRGATGGMAGCCACTGGC
PRI 468 - GGGCCTRGRGTAATCYTTBACAAAGTC
PRI 473 - GCAGCCCTGAAGCTGAGAGATCG
PRI 474 - ACTGACCTGAGACAGGACTCC
PRI 475 - ACACACAGAGCGATCAAATGAGAAACC
PRI 476 - GCTGGTGTCAGAGGTCAATCTGCTTC
PRI 477 - CTGGTCTGGGATACTCGAACACAAAGTC
PRI 490 - GARGAVSTWCCCYGTSCCATTCC
PRI 491 - CAAGKGDYBCATABGGGGARTAAACC
PRI 542 - ATTTATTCTTGTTGCTGCCTGTATTCCC
PRI 543 - TTCCTTGCAGTTGGTCCCTCCA
PRI 636 - CGTTCAGGAGCCATCTCTCAGCA

Degenerate nucleotide codes: R= AG, Y= CT, M= AC, K=GT, S=CG, B=CGT, D=AGT, H=ACT

[2] Not evaluated since sequence known (GenBank accession AF333815)

[3] None of the primer pairs were successful
