# Supplementary material for: Evolution of a Major Drug Metabolizing Enzyme Defect in the Domestic Cat and Other Felidae: Phylogenetic Timing and the Role of Hypercarnivory
Source: PLoS One. 2011 Mar 28;6(3):e18046. doi: 10.1371/journal.pone.0018046 (PMC3065456; doi:10.1371/journal.pone.0018046)
Supplement: Table S6 — Nonsynonymous to synonymous nucleotide substitution frequency ratios (dN/dS) for Carnivora UGT genes obtained using 3 different input tree topologies. (PDF) [file pone.0018046.s009.pdf]

**Table S6.** Nonsynonymous to synonymous nucleotide substitution frequency ratios (dN/dS) for Carnivora UGT genes obtained using 3 different input tree topologies.

| | | | UGT1A6 | | | UGT1A1 | | |
|---|---|---|---|---|---|---|---|---|
| **Input tree** | **Order (sub-order)** | **Family** | **Taxa (n)[1]** | **Seq. (n)[1]** | **dN/dS** | **Taxa (n)[1]** | **Seq. (n)[1]** | **dN/dS** |
| **Maximum likelihood** | | All species (average value) | 49 | 50 | 0.3926 | 47 | 38 | 0.3811 |
| | *Carnivora (Feliformia)* | *Felidae* | 18 | 16 | 0.6785 | 18 | 15 | 0.4476 |
| | | *Hyenidae* | 4 | 4 | 0.508 | 4 | 3 | 0.5258 |
| | | *Herpestidae* | 1 | 1 | 0.2533 | 1 | 1 | 0.2171 |
| | | *Viverridae* | 2 | 2 | 0.4815 | 2 | 2 | 0.3556 |
| | *Carnivora (Caniformia)* | *Ursidae* | 2 | 2 | 0.2148 | 2 | 1 | 0.9952 |
| | | *Procyonidae* | 1 | 1 | 0.4983 | 1 | 1 | 0.2866 |
| | | *Ailuridae* | 1 | 1 | 0.2102 | 0 | 0 | - |
| | | *Mustelidae* | 2 | 2 | 0.3215 | 2 | 2 | 0.2832 |
| | | *Otariidae* | 0 | 0 | - | 3 | 1 | >999 |
| | | *Phocidae* | 2 | 2 | 1.1708 | 2 | 2 | 0.7129 |
| | | *Canidae* | 4 | 4 | 0.1826 | 4 | 3 | 0.1211 |
| | *Non-Carnivora* | | | | | | | |
| | | Cattle, sheep, pig, horse | 4 | 6 | 0.3945 | 1 | 1 | 0.4166 |
| | | Mouse, rat, rabbit | 3 | 4 | 0.3659 | 2 | 2 | 0.4112 |
| | | Primates | 5 | 5 | 0.3969 | 5 | 4 | 0.4127 |
| **Bayesian** | | All species (average value) | 49 | 50 | 0.3922 | 47 | 38 | 0.3826 |
| | *Carnivora (Feliformia)* | *Felidae* | 18 | 16 | 0.6332 | 18 | 15 | 0.4325 |
| | | *Hyenidae* | 4 | 4 | 0.5043 | 4 | 3 | 0.5148 |
| | | *Herpestidae* | 1 | 1 | 0.2546 | 1 | 1 | 0.216 |
| | | *Viverridae* | 2 | 2 | 0.4843 | 2 | 2 | 0.3454 |
| | *Carnivora (Caniformia)* | *Ursidae* | 2 | 2 | 0.2056 | 2 | 1 | 1.3389 |
| | | *Procyonidae* | 1 | 1 | 0.5168 | 1 | 1 | 0.2928 |
| | | *Ailuridae* | 1 | 1 | 0.1952 | 0 | 0 | - |
| | | *Mustelidae* | 2 | 2 | 0.328 | 2 | 2 | 0.2761 |
| | | *Otariidae* | 0 | 0 | - | 3 | 1 | >999 |
| | | *Phocidae* | 2 | 2 | 1.1337 | 2 | 2 | 0.6003 |
| | | *Canidae* | 4 | 4 | 0.1778 | 4 | 3 | 0.1215 |
| | *Non-Carnivora* | | | | | | | |
| | | Cattle, sheep, pig, horse | 4 | 6 | 0.3954 | 1 | 1 | 0.4133 |
| | | Mouse, rat, rabbit | 3 | 4 | 0.3719 | 2 | 2 | 0.4137 |
| | | Primates | 5 | 5 | 0.3925 | 5 | 4 | 0.3543 |
| **Maximum parsimony** | | All species (average value) | 49 | 50 | 0.3889 | 47 | 38 | 0.384 |
| | *Carnivora (Feliformia)* | *Felidae* | 18 | 16 | 0.6512 | 18 | 15 | 0.4378 |
| | | *Hyenidae* | 4 | 4 | 0.5064 | 4 | 3 | 0.5142 |
| | | *Herpestidae* | 1 | 1 | 0.2523 | 1 | 1 | 0.2159 |
| | | *Viverridae* | 2 | 2 | 0.4805 | 2 | 2 | 0.3453 |
| | *Carnivora (Caniformia)* | *Ursidae* | 2 | 2 | 0.1992 | 2 | 1 | 1.3439 |
| | | *Procyonidae* | 1 | 1 | 0.6332 | 1 | 1 | 0.2926 |
| | | *Ailuridae* | 1 | 1 | 0.1262 | 0 | 0 | - |
| | | *Mustelidae* | 2 | 2 | 0.1672 | 2 | 2 | 0.276 |
| | | *Otariidae* | 0 | 0 | - | 3 | 1 | >999 |
| | | *Phocidae* | 2 | 2 | 1.1193 | 2 | 2 | 0.6192 |
| | | *Canidae* | 4 | 4 | 0.1819 | 4 | 3 | 0.1214 |
| | *Non-Carnivora* | | | | | | | |
| | | Cattle, sheep, pig, horse | 4 | 6 | 0.3937 | 1 | 1 | 0.4133 |
| | | Mouse, rat, rabbit | 3 | 4 | 0.4412 | 2 | 2 | 0.3294 |
| | | Primates | 5 | 5 | 0.3621 | 5 | 4 | 0.4688 |

[1] Number of sampled taxa and unique translated sequences (seq.) used in each analysis. Differences between the numbers of taxa and sequences within each group arise from the presence of multiple UGT1A6 genes in mouse (2) and horse (3) (see Tables S1 for details), as well as exclusion of any sequences found to be identical to any other sequence after cropping. Sequences that were excluded from the final analysis (and the identical sequence included in the analysis) were as follows: PanTro_A1 (HomSap_A1), CanRuf_A1 (CanFam_A1), HyaHya_A1 (ParBru_A1), PumCo2_A1 (PumCon_A1), LeoGeo_A1 (LeoTig_A1), PanPar_A1 (PanUnc_A1), PhoHoo_A1 (CalUrs_A1), UrsThi_A1 (UrsMar_A1), LeoGeo_A6 (LeoTig_A6), PriBen_A6 (FelCat_A6).
